# Comparative genomics of Lbx loci reveals conservation of identical Lbx ohnologs in bony vertebrates

**DOI:** 10.1186/1471-2148-8-171

**Published:** 2008-06-09

**Authors:** Karl R Wotton, Frida K Weierud, Susanne Dietrich, Katharine E Lewis

**Affiliations:** 1 King's College London, Department of Craniofacial Development, Floor 27 Guy's Tower, Guy's Hospital, London Bridge, London, SE1 9RT, UK; 2 Cambridge University, Physiology Development & Neuroscience Department, Anatomy Building, Downing Street, Cambridge, CB2 3DY, UK

## Abstract

**Background:**

*Lbx/ladybird *genes originated as part of the metazoan cluster of *Nk *homeobox genes. In all animals investigated so far, both the protostome genes and the vertebrate *Lbx1 *genes were found to play crucial roles in neural and muscle development. Recently however, additional *Lbx *genes with divergent expression patterns were discovered in amniotes. Early in the evolution of vertebrates, two rounds of whole genome duplication are thought to have occurred, during which 4 *Lbx *genes were generated. Which of these genes were maintained in extant vertebrates, and how these genes and their functions evolved, is not known.

**Results:**

Here we searched vertebrate genomes for *Lbx *genes and discovered novel members of this gene family. We also identified signature genes linked to particular *Lbx *loci and traced the remnants of 4 *Lbx *paralogons (two of which retain *Lbx *genes) in amniotes. In teleosts, that have undergone an additional genome duplication, 8 *Lbx *paralogons (three of which retain *Lbx *genes) were found. Phylogenetic analyses of *Lbx *and *Lbx*-associated genes show that in extant, bony vertebrates only *Lbx1*- and *Lbx2*-type genes are maintained. Of these, some *Lbx2 *sequences evolved faster and were probably subject to neofunctionalisation, while *Lbx1 *genes may have retained more features of the ancestral *Lbx *gene. Genes at *Lbx1 *and former *Lbx4 *loci are more closely related, as are genes at *Lbx2 *and former *Lbx3 *loci. This suggests that during the second vertebrate genome duplication, *Lbx1/4 *and *Lbx2/3 *paralogons were generated from the duplicated *Lbx *loci created during the first duplication event.

**Conclusion:**

Our study establishes for the first time the evolutionary history of *Lbx *genes in bony vertebrates, including the order of gene duplication events, gene loss and phylogenetic relationships. Moreover, we identified genetic hallmarks for each of the *Lbx *paralogons that can be used to trace *Lbx *genes as other vertebrate genomes become available. Significantly, we show that bony vertebrates only retained copies of *Lbx1 *and *Lbx2 *genes, with some *Lbx2 *genes being highly divergent. Thus, we have established a base on which the evolution of *Lbx *gene function in vertebrate development can be evaluated.

## Background

Ladybird/Lbx proteins are part of a clustered set of Nk transcription factors, which encompass the Nk4 protein tinman/Nkx2, the Nk3 protein bagpipe/Nkx3, ladybird/Lbx, C15/Tlx, and the Nk1 protein slouch/Nkx1 [1-4]. In protostome genomes, *NK *genes are organised into a cluster, which, along with the molecular phylogeny of Nk homeodomains, indicates that they originated via tandem duplications [2]. Indeed, the *NK *gene cluster is believed to be ancient, with aspects predating the divergence of sponges, cnidarians and bilaterians [5]. In deuterostomes, however, tight clustering has been lost with only *Nkx4/Nkx3 *and *Lbx/Tlx *linkages remaining in the cephalochordate amphioxus and in vertebrates [3].

*Lbx *genes have essential roles in development, including crucial functions in neural and mesodermal cell specification. In mammals and amphibians, *Lbx1 *controls the emigration of specialised, motile muscle precursors that give rise to limb and hypoglossal/tongue muscles, respectively [6-9]. In mouse embryos, *Lbx1 *is also required for the specification of several populations of dorsal spinal cord interneurons, which receive and process somatosensory information from the body [10-13]. Moreover, *Lbx *genes with a similar spinal cord expression pattern to mouse have been described in chick, zebrafish and the spotted dogfish and, in the case of zebrafish and chick, these genes are also expressed in muscle [14-16]. Interestingly, in *Drosophila melanogaster*, a protostome whose ancestors split from the deuterostome lineage over 500 million years ago, two tandem-duplicated *ladybird/Lbx *family members exist (*ladybird early *and *ladybird late*) that are also expressed in developing muscle and neural tissue and are required for the specification of subpopulations of myoblasts and neural cells [1,17]. Thus, *Lbx *genes may have evolutionarily ancient functions in muscle and neural development.

Despite the intriguing similarities of some *ladybird/Lbx *genes, significant differences become apparent when considering all of the members of the *Lbx *family in different vertebrates. For example, mammals, including humans, have two *Lbx *genes [2] as does the chicken [15,18], whereas *Danio rerio *has three *Lbx *genes (this report), and the genome sequencing project for *Xenopus tropicalis *has so far only revealed one *Lbx *gene (this report). In addition, the expression pattern of mammalian *Lbx2 *is distinct from that of *Lbx1 *(*Lbx2 *is expressed in the urogenital system, eye and brain) [19], whereas the two chicken *Lbx *genes and all zebrafish genes are co-expressed in migratory muscle precursors (K. R. Wotton and S. Dietrich, unpublished data). Yet the second chicken *Lbx *gene is not expressed in neural tissues [15,18] while all three of the zebrafish genes are, albeit in distinct regions (K. E. Lewis, unpublished data). Consequently, the evolutionary and functional relationship of *ladybird/Lbx *genes is not clear.

It is generally believed that at the base of the vertebrate lineage, the entire genome was duplicated twice (2R hypothesis; [20]). This was followed by a further genome duplication in the ray-finned fish lineage at the base of the teleost radiation [21-23]. In all chordate relatives of vertebrates investigated so far, such as the urochordates *Oikopleura dioica *and *Ciona intestinalis *and the cephalochordate *Branchiostoma floridae *(amphioxus), only a single *lbx *gene has been found [3]. It is, therefore, safe to assume that prior to the two rounds of vertebrate genome duplication, only a single *Lbx *gene existed.

One prediction of the 2R hypothesis is that a 4:1 ratio of genes should have been present in an ancestral vertebrate when compared to their invertebrate relatives. However, with gene duplicates carrying redundant functions, many of these four copies (called ohnologs to indicate that they are paralogs that have originated by a process of whole-genome duplication – see [24,25]) were lost. Yet, despite considerable interest in *Lbx *gene function in extant vertebrates, orthologies between *Lbx *genes have not been established. Therefore, it is not known whether different vertebrate lineages have lost the same, or different, *Lbx *ohnologs. This raises two alternative hypotheses for the differences that have been observed between vertebrate *Lbx *genes.

1. Different vertebrate lineages have retained different *Lbx *ohnologs with distinct expression patterns and/or functions

2. The same *Lbx *ohnologs have been maintained in all vertebrates with differences in expression or function being due to more recent neo- and/or sub- functionalisation.

To distinguish between these possibilities, we have used a number of bioinformatics approaches to characterise the organisation of *Lbx *loci in extant osteichthyan vertebrates and to determine the relationship of *Lbx *genes and genes associated with *Lbx *loci. Our studies identified non-*NK *genes that were acquired by *Lbx/Tlx *region(s) prior to, or during, the two rounds of vertebrate genome duplication and that hence, serve as signature genes for these loci. With the help of these signature genes, the remnants of all four *Lbx/Tlx *paralogons (eight in teleosts) were identified. Phylogenetic analyses of *Lbx*, *Tlx *and the co-localising non-*Nk *genes revealed that the first round of whole genome duplication in vertebrates created the ancestor of the *Lbx1/Tlx1 *and *Lbx4/Tlx4 *clusters plus the ancestor of the *Lbx2/Tlx2 *and *Lbx3/Tlx3 *clusters. After the second genome duplication, *Lbx *and *Tlx *genes were lost, such that before the split of the ray-finned and lobe-finned fish lineages only *Lbx1/Tlx1*, *Lbx2/Tlx2 *and *Tlx3 *genes were maintained. Gene loss also occurred after the additional genome duplication in the ray-finned fish lineage, leaving teleosts (including zebrafish and pufferfish) with two *Lbx1 *and *Tlx3 *genes but only one *Lbx2, Tlx1 *and *Tlx2 *gene. Since the amniote *Lbx2 *genes diverge much more in their coding sequences than the *Lbx1 *genes and the expression pattern of mouse *Lbx2 *is distinct from non-mammalian *Lbx2 *genes, we propose that amniote (or possibly sarcopterygian) *Lbx2 *genes have evolved at a faster rate and were subject to neofunctionalisation. *Lbx1 *genes on the other hand may have retained more features of the original chordate *lbx *gene.

## Results

### Identification of *Lbx *genes in extant Osteichthyes

To reconstruct the phylogeny of vertebrate *Lbx *genes we first attempted to identify the complete set of these genes in representatives of extant Osteichthyes. For this, BLAST-searches of sequence databases were carried out, using the known human and mouse Lbx1 and Lbx2, chicken Lbx1 and Lbx3, *Xenopus laevis *Lbx1 and zebrafish Lbx1 sequences as query sequences [1,2,4,9,14,15,18,19]. To obtain outgroups for these phylogenetic analyses, we also searched the databases for lbx/ladybird sequences in invertebrate deuterostomes, including the cephalochordate *Branchiostoma floridae *(amphioxus), the urorchordates *Oikopleura dioica *and *Ciona intestinalis*, and the echinoderm *Strongylocentrotus purpuratus *(purple sea urchin); moreover we included sequences from various protostomes. Our search confirmed the presence of two distinct *Lbx *genes in placental mammals and marsupials (human, mouse, dog, cattle, opossum), one *Lbx *gene in the still incompletely sequenced platypus and Anole lizard genomes, and two *Lbx *genes in the chicken. Only one *Lbx *gene was found for the frog *Xenopus tropicalis*. On the other hand, besides the gene so far known as zebrafish *lbx1 *[14], two novel *lbx *genes were identified in this organism. Three *Lbx *genes were also identified in the teleosts *Takifugu rubripes *(fugu), *Tetraodon nigroviridis*, *Gasterosteus aculeatus *(stickleback), and two genes in *Oryzias latipes *(Medaka), while only one *lbx/ladybird *gene was retrieved for the invertebrate deuterostomes.

### Phylogenetic analysis of osteichthyan *Lbx *protein sequences

**Figure 1:**
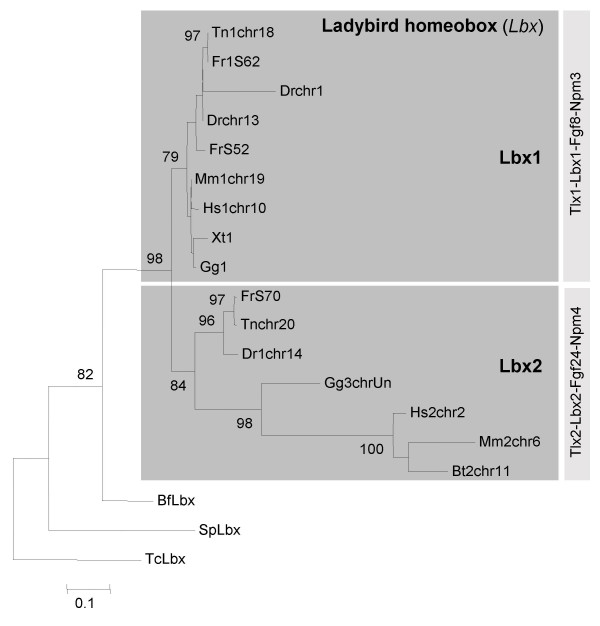
**Molecular phylogenetic analysis of *Lbx *sequences**. The tree shows a maximum likelihood analysis of Lbx protein sequences. Dark grey boxes indicate orthologous groups while light grey boxes indicate gene linkage. Bootstrap values below 70 have been removed. Only two *Tetraodon *Lbx proteins are included as the third sequence (found on scaffold 8483) is incomplete. Note that the Lbx sequences separate into two clear groups, Lbx1 and Lbx2, which are supported by bootstrap values of 79 and 83 (boxed), respectively. The chicken protein previously denoted as Lbx3 [18], the zebrafish protein previously denoted as Lbx1 [14] and one of the fugu, *Tetraodon*, stickleback and medaka sequences group with mammalian Lbx2 sequences. The two novel zebrafish Lbx sequences and the remaining teleost sequences group with mammalian, chicken and frog Lbx1. The tree shows significantly longer branch lengths for the Lbx2 proteins indicating that these sequences may be evolving at a quicker rate than the Lbx1 proteins. For common names of species see additional file 2.

To determine the evolutionary relationship between osteichthyan *Lbx *genes, we first determined the phylogenetic relationship of Lbx proteins (Fig. 1). For this purpose, amino acid sequences were aligned and analysed, using maximum likelihood methods. We found that the vertebrate Lbx sequences were assigned to two distinct groups. The first group encompassed the known human, mouse, chicken and frog Lbx1 proteins. In addition, this group contained the two novel zebrafish Lbx proteins, encoded by the genes located on chromosomes 13 and 1, the fugu Lbx proteins whose genes are on scaffolds 52 and 62, the *Tetraodon *protein encoded by the *Lbx *gene on chromosome 18, the stickleback proteins with genes on groups VI and IX and the medaka protein encoded by the gene on chromosome 1.

The second group contained all of the mammalian Lbx2 proteins, the chicken protein currently known as Lbx3, the zebrafish protein so far named Lbx1 and encoded by the gene on chromosome 14, together with the Lbx sequences encoded by genes on fugu scaffold 70, *Tetraodon *chromosome 20, stickleback group IV and medaka scaffold 1066. The division into two distinct groups of Lbx proteins was supported by high bootstrap values. Our phylogenetic analysis shows significantly longer branch lengths for the amniote Lbx2 proteins, indicating that these *Lbx2 *genes have probably evolved at a quicker rate than the *Lbx1 *genes. No evidence was found for distinct Lbx3/4 proteins.

### Comparison of genomic *Lbx *loci in Osteichthyes

It is generally held that in the lineage leading to jawed vertebrates, two rounds of whole genome duplication occurred, followed by a further genome duplication in the lineage leading to teleosts [20-23,26]. Thus, theoretically, if no gene loss has occurred, four *Lbx *genes should be detectable in extant tetrapods and eight in teleost fish. However, our phylogenetic analyses suggest that only two *Lbx *genes were retained after the second vertebrate genome duplication and before the genome duplication in ray-finned fish. To confirm these results and to further analyse the orthology of the various vertebrate *Lbx *genes, we compared the organisation of genes associated with the vertebrate *Lbx *loci, reasoning that orthologous *Lbx *genes would share a similar chromosomal environment, while paralogous genes would exhibit a distinct arrangement of the locus (see [27-29] for examples).

**Figure 2:**
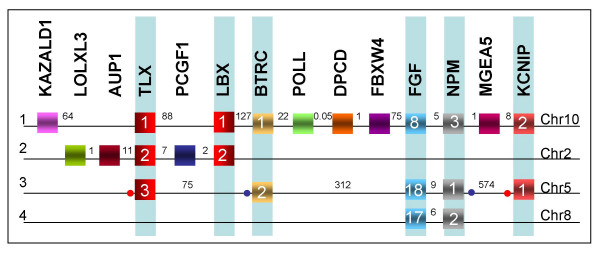
**Human paralogons of the *LBX/TLX *cluster loci**. Schematic representation of the four human *LBX/TLX *paralogons plus paralogous genes occurring at the *NKX3.2 *locus, deduced from analysis of the human genome using NCBI map viewer *Homo sapiens *Build 36.2 (September 2006). Genes are represented by boxes with gene names appearing above and gene subfamilies indicated by numbers inside. Letters on the far left indicate paralogon designation, while chromosomal locations are indicated on the far right. Numbers between genes are approximate intergenic distances in Kb. Background shading indicates paralogous genes. Blue and red dots indicate sites of inversions. Note that *LOXL1, AUP1 *and *PCGF1 *genes appear only at the *LBX2 *locus and *KAZALD1, POLL, DPCD, FBXW4 *and *MGEA5 *appear only at the *LBX1 *locus. *BTRC, FGF, NPM *and *KCNIP *genes are linked with *LBX *and *TLX *genes at more than one locus, suggesting that these genes were acquired by the *LBX/TLX *cluster during or before the two rounds of vertebrate genome duplication. Also note that a *KCNIP4 *and a *SLIT2 *gene are associated with *NKX3.2 *on chromosome 4. A *KCNIP3 *gene is located on the long arm of chromosome2, however at a distance to *LBX2*, suggesting that it is not an original component of the *LBX2 *paralogon.

**Figure 3:**
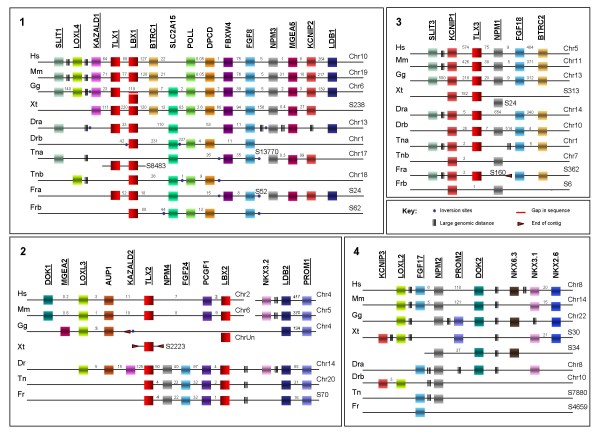
**Genomic organisation of human LBX/TLX cluster paralogons and putative orthologous counterparts**. Genomic organisation of human (Hs) *Lbx/Tlx *paralogons and the putative orthologous counterparts identified in *Mus musculus *(Mm), *Xenopus tropicalis *(Xt), *Gallus gallus *(Gg), *Danio rerio *(Dr), *Tetraodon nigroviridis *(Tn) and *Takifugu rubripes *(Fr). For simplicity, only three teleosts are included in this figure: additional data for *Oryzias latipes *(medaka) and *Gasterosteus aculeatus *(stickleback) can be found in additional file 1. The main genes characteristic of each paralogon are included in the figure; additional genes and linked genes found further away can be found in additional file 1. Schematic representations of the orthologous genomic regions are depicted in panels 1–4, which correspond to human paralogons 1–4 in figure 2. Gene orthology is indicated by colour code and was inferred from molecular phylogenetics (see additional files). Genes appearing in more than one orthologous region are underlined. Numbers at the ends of each line indicate chromosome (chr) or scaffold (s) numbers. A parallel red line marks gaps in the sequence, while triple black lines indicate large intergenic distances. Blue dots mark sites of inversions, red arrows the end of a contig. Boxes off-line represent genes for which genomic sequences are incomplete; where no chromosome or scaffold number is given, no linkage data is available. In some cases, "missing" genes can be found on different scaffolds or chromosomes. This data is shown in additional file 1. In addition, we do not show the dispersed 2^nd ^teleost Lbx2 paralogon that no longer contains an Lbx or Tlx gene, but this information is included in additional file 1. The genes our phylogenetic analysis identified as *Lbx1 *are found in a similar genomic environment, linked to orthologs of the genes present at the human *LBX1/TLX1 *locus. In addition, non-mammalian *Lbx1 *genes are linked to the *Slc2a15 *gene not found at any other *Lbx *locus. *Lbx2 *genes are linked to *Loxl3, Aup1 *and *Pcgf1 *genes (genomic information incomplete for chicken and frog), while *Tlx3 *genes are linked to the orthologs of human *KCNIP1, NMP1, FGF18 *and *BTRC2*. In teleosts, *Fgf24 *and *Npm4 *are part of the *Lbx2 *locus, while in all species examined, *Fgf17 *and *Npm2 *are no longer linked to *Lbx/Tlx *genes.

Since the human genome information is the most accurate and complete, we began by recording the genes that according to the NCBI Map Viewer database are located in the environment of human *LBX1 *and *2 *genes (Fig. 2). Subsequently, we looked for remnants of the *LBX3 *and *4 *loci, initially by searching the human genome for paralogues of the genes co-localising with *LBX1 *and *2*. We then identified and determined the arrangement of genes characteristic for each of these four paralogons in the genomes of other mammals, the chicken, the frog *Xenopus tropicalis *and the five teleosts (Fig. 3 and additional file 1).

### *Lbx *loci in humans

Human *LBX1 *has previously been mapped to chromosome 10, 1.7 Mb distant from *NKX2.3 *[2,4]. *LBX1 *is tightly linked to the related Nk-type homeobox gene *TLX1 *(Fig. 2 and Additional file 1). This is followed by the Kazal-type serine peptidase inhibitor domain 1 gene *KAZALD1*, the *LOXL4 *gene encoding a Lysyl oxidase like protein and the SLIT1 gene encoding an axon guidance molecule. Moving in the opposite direction, *LBX1 *is linked to a Beta-transducin repeat containing gene (*BTRC1*, a F-box and WD repeat domain 11 type gene), the DNA polymerase lambda gene (*POLL*), the Deleted in a mouse model of Primary Ciliary Dyskinesia gene (*DPCD*), the F-box and WD repeat domain containing 4 gene (*FBXW4*), the Fibroblast growth factor 8 gene (*FGF8*), the Nucleophosmin 3 gene (*NPM3*), the Meningioma expressed antigen 5 gene (*MGEA5*), the gene encoding a Kv channel interacting protein 2 (*KCNIP2*), and further away, the gene encoding the Lim domain binding protein LDB1 and the *NKX1.2 *gene (Figs 2 &3).

Human *LBX2 *has been mapped to chromosome 2, and is linked to *TLX2 *([2,4]; Fig. 2). Neither of these genes are associated with other *NK *genes, suggesting that they have translocated from their original cluster [2,3]. *LBX2 *and *TLX2 *are separated by the *PCGF1 *gene, which encodes a polycomb group ring finger. Facing away from *PCGF1 *and *TLX2*, *LBX2 *is linked to genes that encode Dynactin subunit 1 (DCTN1) and the Tetratricopeptide repeat protein 31 (TTC31). *TLX2 *on the other side is flanked by the *DEAQ box 1 *(*DQX1*) gene which encodes for an ATP-dependent RNA helicase, the gene encoding Ancient ubiquitous protein 1 (AUP1), the *HTRA1 *gene which encodes for a high temperature requiring serine protease, the *LOXL3 *gene, *DOK1 *which encodes for the Docking downstream of Tyrosine kinase 1 protein, and a gene encoding the uncharacterised protein NP620159.2. Thus, the human *LBX1 *and *LBX2 *loci are quite distinct (Fig. 2). However, they both harbour *TLX *genes, in line with the idea that *Lbx *and *Tlx *genes were linked in the original bilaterian cluster of Nk-type homebox genes [2,3]. Moreover, both *LBX *loci include *LOXL *genes.

In humans, a further *TLX *gene exists 1.4 Mb distant from *NKX2.5 *on chromosome 5, thought to be a remnant of the third cluster of *NK *genes, which, after the second vertebrate genome duplication, lost its cognate *LBX *gene ([3]; Fig. 2, Additional file 1). Investigating the organisation of this former *LBX3/TLX3 *locus, we found that *TLX3 *is on one side linked to the gene that encodes for Ran-binding protein 17 (RANBP17, a member of the Exportin protein family), the gene encoding for the gamma-aminobutyric acid receptor (GABRP), a further *KCNIP *gene, *KCNIP1*, the Lymphocyte cytosolic protein gene *LCP2*, the Forkhead transcription factor gene *FOXI1*, the Dedicator of cytokinesis gene *DOCK2*, the coil-coil domain encoding *CCDC99 *gene, followed by *SLIT3*. On the other side, *TLX3 *is linked to *NMP1*, *FGF18, FBXW11/BTRC2*, and further away from the *TLX3 *gene, *NKX2.5*, *MSX2 *and *DOK3*. The order of *NMP1*, *FGF18 *and *FBXW11/BTRC2 *is reversed compared to the paralogous genes at the *LBX1*/*TLX1 *locus. However, it is remarkable that for both the *LBX1/TLX1 *and *LBX3/TLX3 *paralogons, the *NPM *and *FGF *genes are closely linked, separated by sequences of only 5 and 9 kb, respectively. Taken together, our data suggests that *Kcnip*, *Fbxw11/Btrc2*, *Slit, Loxl, Dok *and the closely associated *Fgf-Npm *genes are hallmarks of *Lbx/Tlx *loci.

To identify remnants of the fourth *LBX *locus, we searched the human genome for additional, linked *KCNIP*, *BTRC/FBXW11*, *SLIT*, *LOXL*, *DOK *and *FGF*-*NPM *sequences. No further homologues of *BTRC/FBXW11 *genes were identified. However, we found:

- *FGF17 *and *NPM2 *closely linked to *DOK2 *and about 1.8 Mb distant from *LOXL2, NKX2.6 *and *NKX3.1 *on chromosome 8, which also carries NKX6.3

- Linked *KCNIP4-SLIT2 *genes 7.7 Mb distant from *NKX3.2 *on chromosome 4

- *KCNIP3 *on the long arm of chromosome 2, however 20.9 Mb distant from and hence probably not a genuine part of the *LBX2-TLX2 *region.

A previous report suggested that *LBX2/TLX2 *may once have belonged to the *NKX2.6-NKX3.1 *region on chromosome 8 [3]. However, the presence of *LOXL *and *DOK *genes at both the *LBX2 *locus and the *NKX2.6-NKX3 *locus is not consistent with this idea. *KCNIP4-SLIT2 *were found linked to the *Prominin *gene *PROM1*, so far not associated with *LBX/TLX *loci. However, as *KCNIP3 *is also linked to *PROM2*, this suggests that *Prom *genes may once have belonged to *Lbx/Tlx *containing regions. Taken together, these observations suggest that the *LBX2/TLX2 *region may have originated from the *NKX3.2 *containing cluster on chromosome 4, while *FGF17*-*NPM2 *linked to *LOXL2 *and *DOK2 *represent the remnants of the fourth *LBX/TLX *region associated with *NKX2.6 *and *3.1 *on chromosome 8.

### *Lbx *loci in other mammals

Investigating the arrangement of genes at *Lbx *loci in additional placental mammals (mouse, dog and cattle), a marsupial (opossum) and a monotreme (platypus), we found that the loci are arranged in the same fashion as in humans (Fig. 3, Additional file 1 and data not shown). The exception is the platypus *Lbx2/Tlx2 *locus, whose existence could not be confirmed as genes linked to this paralogon in other mammals were found on short, unlinked and poorly characterised DNA fragments. Moreover, in mouse and *Monodelphis*, *Prom2-Kcnip3 *are not on the same chromosome as the *Lbx2 *locus, supporting the idea that these genes secondarily intercalated into the *Lbx2 *carrying chromosome in the lineage leading to humans.

### *Lbx *loci in the chicken

As the information on the chicken genome is still fragmentary, the localisation of chicken *Lbx *genes could not be determined with certainty. However, chicken chromosome 6 contains a region that is syntenic with the *LBX1 *region on Human chromosome 10, encompassing *Kazald1, Tlx1, Btrc, Poll, Dpcd, Fbxw4, Fgf8, Nmp3, Mgea5 *and *Kcnip2 *in the same order as the human genes (Fig. 3). *Loxl4, Slit1 *and further genes located in the wider environment of the mammalian *Lbx1 *locus were also identified, with gene groups displaying a similar arrangement in the chicken and in mammals (Additional file 1). Furthermore, we identified sequences 300 bp upstream and 2.2 kb downstream of the two *Lbx1 *exons, which are conserved between the mammalian *Lbx1 *loci and the putative chicken *Lbx1 *site; in the chicken these regions flank a region that has not been fully sequenced yet (data not shown). Thus, it is likely that this area on chromosome 6 indeed harbours the *Lbx1 *gene. As will become relevant below, in the chicken the *Btrc1 *gene is separated from the *Poll *gene by the *Slc2a15 *gene (our name) encoding a solute carrier (Fig. 3).

The *Lbx2*-type gene so far named *Lbx3 *[18] is situated on a short contig not assigned to a chromosome (Fig. 3). However, *Aup1, Htra2, Loxl3*, the gene encoding NP620159.2, together with a further *Mgea *gene, were found on the so far poorly-characterised contig 242 assigned to chromosome 4; all genes are arranged in the same order as the corresponding genes at the *Lbx2/Tlx2 *containing region of mammalian chromosomes (Fig. 3; Additional file 1). Moreover, separated by sequence gaps, these genes are linked to the *Dctn1 *gene, which is downstream of *Lbx2 *in mammals. Significantly, this chromosome also carries genes associated with the *NKX3.2 *locus in humans, adding weight to the hypothesis that *Lbx2 *genes were once associated with this particular *NK *cluster.

*Tlx3 *was found on chicken chromosome 13 in a region syntenic with the *TLX3 *containing region of human chromosome 5 and grouped with *Fbxw11/Btrc2, Fgf18, Npm1, Ranbp17, Gabrp, Kcnip1, Foxi1, Lcp2, Dock2, Ccdc99 and Slit3 *in the same fashion as mammalian *Tlx3*. In agreement with this being the third *Lbx/Tlx *paralogon, other members of the *NK *cluster associated with this paralogon are also found on chick chromosome 13 (Additional file 1).

Finally, a representative of *Fgf17 *could not be identified, but *Npm2*, *Dok2 *and *Loxl2 *were found on chromosome 22, in a region syntenic to the *FGF17-NPM2 *region on human chromosome 8 and linked to *Nkx2*.8 and *6.3 *(Fig. 3 and Additional file 1). Notably, this region is also linked to *Prom2*, a gene in mammals associated with *Kcnip3 *(*Kcnip3 *was not found in the chicken). This finding supports the idea that *Kcnip3-Prom2 *are not an original component of the *Lbx2/Tlx2 *paralagon but instead probably belong to the fourth *Lbx/Tlx *paralagon.

### *Lbx *loci in *Xenopus tropicalis*

In the frog, both *Lbx1 *and the *Kazald1, Tlx1, Btrc, Poll, Dpcd, Fbxw4, Fgf8, Nmp3, Mgea5 *and *Kcnip2 *genes typical for the amniote *Lbx1 *locus were found on scaffold 238 (Fig. 3 and Additional file 1). As in the chicken, the *Slc2a15 *gene was located between *Btrc *and *Poll*, suggesting that the solute carrier gene was present in the original *Lbx1 *locus and was lost from this location in the lineage leading to mammals.

A *Tlx2 *gene was found on the scaffold 2223, but this scaffold does not contain any further genes associated with *Lbx/Tlx *paralagons (Fig. 3 and Additional files). Similarly, an isolated *Pcgf1 *gene was found on scaffold 47. Searches for *Loxl3 *and *Aup *genes were unsuccessful. Thus, the existence of a *Lbx2/Tlx2 *locus could not be determined. However, linked *Tlx3, Gabrp, Kcnip1, Foxi, Dock2 and Ccdc99 *genes were found on scaffold 313, suggesting that a *Lbx3/Tlx3 *locus exists in frogs (Fig. 3).

As in chicken, we did not find a *Xenopus tropicalis *ortholog of *Fgf17*, but we detected *Npm2 *and *Dok2 *on scaffold 34, linked to each other and arranged in the same order as the corresponding genes surrounding human and chicken *Npm2*. Notably, as in amniotes, these genes are also linked to *Nkx6.3 *(Additional file 1). Moreover, scaffold 30 contains linked *Loxl2*, *Nkx3.1*, *Nkx2.6*, *Prom2 *and *Kcnip3 *genes, which in amniotes, with the exception of *Prom2-Kcnip3 *that have translocated to a different chromosome in placental mammals, are linked to *Fgf17, Npm2, Dok2 *and *Nkx6.3 *(Additional file 1). Taken together, this suggests that all of these genes belong to the same *Nkx3.1, 2.6 *and *6.3 *containing cluster.

### *Lbx *loci in teleost fish

For teleosts, we expected to find chromosomal arrangements corresponding to that of tetrapods, if, in line with our analysis of *Lbx *proteins, only two *Lbx *bearing loci were present prior to the genome duplication in the ray-finned fish lineage. Alternatively, if in teleosts *Lbx3 *and *Lbx4 *genes were maintained, they should be embedded in distinct loci, with *Lbx3 *next to *Tlx3*, *Kcnip1*, *Npm1*, *Fgf18 *and *Btrc2 *and/or *Lbx4 *near to *Fgf17 *and *Npm2*.

We found for the two novel *Lbx1*-type teleost genes that the organisation of their gene loci fell into two classes. The genes on zebrafish chromosome 13, *Tetraodon *scaffolds 8483 and 13770, fugu scaffold 52 and stickleback group VI are surrounded by *Tlx1 *(sequence incomplete for *Tetraodon*), *Slc2a15*, *Fbxw4 *and *Fgf8*, thus bearing the hallmarks of the tetrapod *Lbx1/Tlx1 *locus (Fig. 3 and additional files). The same arrangement of genes was found on medaka chromosome 15, with a sequence gap at the place where the *lbx *gene is expected to be. Significantly, in all of these teleosts the order of genes is identical to that of the equivalent tetrapod genes, with the exception of an inversion between the *Tlx1 *and *Fgf8 *region (Figure 3). The *Lbx *genes on zebrafish chromosome 1, *Tetraodon *chromosome 18 (sequence incomplete), fugu scaffold 62, stickleback group IX and medaka chromosome 1 are linked to a second *Slc2a15 *gene, and to *Poll*, *Dpcd *and a second *Fgf8*-type gene (there is a sequence gap at the position of the *Fgf8 *gene in *Tetraodon*), thus also bearing hallmarks of the tetrapod *Lbx1/Tlx1 *locus (Fig. 3 and additional files). Both stickleback and medaka loci are accompanied by *Kcnip2 *and *Mgea5 *genes; for stickleback group VI and medaka chromosome 15, these genes are also associated with *Npm3 *and *Slit1*, while linked *Npm3-Mgea5 *genes were found on zebrafish chromosome 13. Moreover, all of the teleost *Lbx1*-type loci are associated with gene groups found in the wider environment of *Lbx1 *loci in amniotes (Additional file 1). Taken together, these findings support the idea that all of these fish have two *Lbx1/Tlx1 *paralogons, although in many cases there has been reciprocal gene loss following the teleost-specific whole genome duplication

For the third of the teleost *Lbx *genes, namely the gene currently known as zebrafish *Lbx1 *on chromosome 14 and the *Lbx *genes on *Tetraodon *chromosome 20, fugu scaffold 70, stickleback group IV and medaka scaffold 1066, we found an environment reminiscent of the mammalian *Lbx2 *locus, with the *Lbx *genes being separated from a *Tlx *gene by *Pcgf1 *(Fig. 3). The exception is the medaka scaffold 1066, which is a short fragment that only contains the *Lbx *and *Pcgf *genes. Different from that of mammals, an *Fgf24*-*Npm4 *gene set was found between the *Lbx *and *Tlx *genes. In the wider environment of the *Lbx-Pcgf1-Fgf24-Npm4-Tlx *gene set, we found two types of arrangement (Additional file 1). On zebrafish chromosome 14, the genes are associated with *Aup1 *and *Loxl3*, reminiscent of the mammalian *Lbx2/Tlx2 *paralagon. The genes on fugu scaffold 70, *Tetraodon *chromosome 20 and stickleback group IV, are associated with *Nanos1, Limch1, Phox2b, Tmem33, Bbs7, Anxa5, Fgfbp, Prom1, Tapt1, Ldb2 *(*Lbx1 *side) and *Adad1, Spata5, Ankrd50, Leprot1, Srp72, Arl9, Hop, Sec24B *(*Tlx *side); the ultracontigs 115 and 117 and chromosome 10 of medaka harbour the same genes in the same order. Notably, the genes surrounding the *Lbx-Pcgf-Fgf-Npm-Tlx *group in fugu, *Tetraodon*, stickleback and medaka are also found on zebrafish chromsome 14, while *Aup1 *is found on stickleback group IV and medaka chromosome 10 (Additional file 1), i.e. all of these genes were located in the wider environment of the *Lbx-Pcgf-Fgf-Npm-Tlx *locus. Taken together, our data suggest that, despite substantial gene rearrangements, these chromosomal regions in teleosts are *Lbx2/Tlx2 *paralogons. Interestingly, the genes closely associated with fugu, *Tetraodon*, stickleback and medaka *Lbx2*, including the *Fgfbp-Prom1-Tapt1-Ldb2-Anxa5 *genes, are linked with the *Nkx3.2 *locus on human chromosome 4 and chicken chromosome 4, consistent with our proposal that the mammalian *Lbx2 *genes were originally part of the *Nkx3.2 *cluster (additional file 1).

Searching for remnants of the second teleost *Lbx2 *paralagon, we found *Loxl3*, a further *Nanos1 *gene and *Ttc31 *on *Tetraodon *chromosome 10, fugu scaffold 338, stickleback group XV and medaka chromosome 22. *Dok1-NP620159.2-Sema4f *genes were found on *Tetraodon *scaffold 7074, fugu scaffold 186, stickleback group VII and medaka chromosome 18; in stickleback and medaka these genes were also linked to *Kcnip4-Gba3-Gpr125*, which in tetrapods co-localise with *Nkx3.2 *(Additional file 1; the scaffolds were too short to determine whether this linkage also exists for fugu and *Tetraodon*). Finally, at a distance from the second *Lbx1 *locus on zebrafish chromosome 1, *Tetraodon *chromosome 18, fugu scaffold 17, stickleback group IX and medaka chromosome 1, we found *Slit2 *and a second group of *Fgfbp-Prom1-Tapt1-Anxa5 *genes linked to *Fbxw7*, which in humans are all associated with the *LBX2 *locus. Thus, it seems that the second teleost *Lbx2 *paralagon has dispersed, with parts having translocated to one of the *Lbx1 *carrying chromosomes.

For *Tlx3 *genes, two types of arrangements were identified (Additional file 1). On zebrafish chromosome 14, fugu scaffold 160, *Tetraodon *chromosome 1, stickleback group IV and medaka chromosome 10, i.e. inserted into the *Lbx2 *containing chromosome, *Tlx3 *is on one side linked to *Kcnip1*, and on the other linked to the *Hrh2 *gene encoding a Histamine receptor, *Dock2 *and, slightly more distant, *Fgf18 *and *Fbxw11*. On zebrafish chromosome 10, fugu scaffold 6, *Tetraodon *chromosome 7, stickleback group VII and medaka chromosome 14, *Hrh2 *is linked with *Kncip1*, *Npm1 *and in the case of zebrafish, *Tlx3b*, *Fgf18 *and *Fbxw11*, suggesting that remnants of the two *Tlx3 *loci created by the teleost-specific genome duplication still exist.

Finally, searching for additional *Btrc, Prom, Loxl, Slit, Dok, Kcnip*, and *Fgf-Npm *genes as indicators of other possible *Lbx/Tlx *paralogons, we identified linked *Npm2-Dok2*-*Fgf17 *genes on zebrafish chromosome 8, linked *Fgf17-Loxl2-Kcnip3 *genes on stickleback group XIII, and linked *Kcnip3-Loxl2-Dok2 *genes on *Tetraodon *chromosome 12 and medaka chromosome 9. A second set of linked *Kcnip3-Loxl2-Dok2 *genes was identified on zebrafish chromosome 10 and a second set of *Loxl2 *and *Kcnip3 *genes in a conserved environment but split between two chromosomes were also identified for the other four fish species. Notably, additional genes were also identified that co-localise with *Loxl2, Dok2, Kcnip3*, *Fgf17 *and *Npm2 *genes both in teleosts and tetrapods, including orthologs of *Nkx2.6 *(Additional file 1 and data not shown), supporting the idea that all these genes once belonged to the same locus, which in tetrapods still encompasses *Nkx 2.6, 3.1 *and *6.3*.

### Phylogenetic relationship of signature genes for *Lbx/Tlx *paralogons

Characterising *Lbx/Tlx *loci in tetrapods, we found evidence for three distinct paralogons, with the possibility of *Fgf17-Npm2 *representing the remnant of the fourth. In teleosts, the arrangement of *Lbx1/Tlx1 *and *Tlx3 *genes closely follows the pattern observed in tetrapods. However, the putative teleost *Lbx2/Tlx2 *paralogon (genes currently named *Lbx1 *and *Tlx3a *in zebrafish) includes additional *Fgf-Npm *genes. Moreover, all of the teleosts except zebrafish show a somewhat divergent organisation of genes in the chromosomal regions surrounding the *Lbx2/Tlx2 *loci. Furthermore, the duplicate *Lbx2 *paralogon and the two *Fgf17-Npm2 *paralogons are poorly preserved in teleosts. Therefore, to further confirm the evolutionary relationship of the *Lbx *loci and our assignment of genes to specific paralogons, we carried out a comprehensive phylogenetic analysis of protein sequences encoded by the genes in the various *Lbx/Tlx *regions. For this, we BLAST-searched sequence databases for related sequences and built phylogenetic trees which included various invertebrate outgroups (Additional file 2).

### Phylogenetic relationship of genes co-localising with several *Lbx *paralogons

*Btrc/Fbxw11, Mgea, Kazald*, *Ldb *and *Prom *genes occurred at two, *Tlx, Loxl, Slit *and *Dok *genes at three and *Kcnip*, and the linked *Fgf-Npm *genes at four putative *Lbx *loci in tetrapods. If our assignment of *Lbx/Tlx *paralogons is correct, then the genes that we have assigned to particular paralogons should group together. In addition, if the fundamental lay-out of *Lbx/Tlx *paralogons was established in vertebrates before the divergence of lobe-finned and ray-finned fish and the additional genome duplication in the ray-finned fish lineage, then the teleost protein sequences should group with the tetrapod proteins. If, however, distinct *Lbx/Tlx *loci were maintained in the lineage leading to lobe-finned fish/tetrapods and ray-finned fish/teleosts, then our phylogenetic analysis should reveal additional groups of genes.

With the exception of Dok2 sequences, which seem to have become highly diverged, all of our analyses supported our assignment of the different *Lbx/Tlx *paralogons (discussed in detail in Additional file 2). Notably, our analyses of *Ldb *and *Prom1 *genes also support our model that the tetrapod *Lbx2 *loci was originally located in the *Nkx3.2 *containing cluster: the *Ldb2 *sequences, which are associated with *Lbx2 *in teleosts and with the now *Lbx*-less locus carrying *Nkx3.2 *in tetrapods group together, as do the *Prom1 *genes which are linked to *Ldb2-Tapt-Anxa5 *genes and hence, to the current plus the dispersed *Lbx2 *and *Nkx3.2 *loci (Fig 3). In addition, our analyses of *Prom2*, *Tlx*, *Kcnip*, *Fgf *and *Npn *genes shows that orthologues associated with the *Lbx1 *and *Lbx4 *paralogons are more closely related to each other than to orthologues associated with the *Lbx2 *and *Lbx3 *paralogons and vice versa. This is most informative for the genes where four paralogues are still linked to potential Lbx loci in extant vertebrates (*Fgf*, *Npm *and *Kcnip *genes). In these cases, we found that *Kcnip1 *and *Kcnip4 *genes are more closely related to each other than to *Kcnip2 *and *3*. Similarly, Fgf8 sequences are closely related to Fgf17 sequences, and Fgf24 sequences are closely related to Fgf18. Finally, the Npm3-Npm2 and Npm4-Npm1 sequences are more closely related to each other, respectively (Additional file 2). Taken together, this suggests that the *Lbx1/4 *paralogons and the *Lbx2/3 *paralagons arose from different ancestral *Lbx *loci generated during the first vertebrate genome duplication.

## Discussion

*Ladybird/Lbx *genes are crucial regulators of metazoan neural and muscle cell specification [6-9]. Yet before this study, it was difficult to evaluate the basic role of *Lbx *genes in vertebrate development and evolution, as it was unclear exactly how many *Lbx *genes exist in different vertebrates and how these genes are phylogenetically related to each other. The 2R hypothesis predicts that early in the vertebrate lineage, four ohnologs were generated for each gene in the genome; for teleosts, descendants of ray-finned fish, a further genome duplication should have produced eight gene copies [3,20-23,26,30-38]. However four ohnologs are seldom found in vertebrate genomes, suggesting that gene loss has played an important part in genome evolution. In recent years, two *Lbx *genes were identified in amniotes, one in frogs and one in the teleost *Danio rerio *[1,2,9,14,15,18,19]. One of the amniote *Lbx *genes and the *Lbx *genes identified in frog and zebrafish had been reported to be *Lbx1 *genes, while the second of the amniote *Lbx *genes had been suggested to represent distinct *Lbx2 *(mammals) and *Lbx3 *genes (birds) with rather divergent expression and function [18,19]. This suggested that *Lbx1, 2 *and *3 *genes were retained in amniotes up to the split of mammalian and avian lineages, while despite their additional genome duplication ray-finned fish seemed to have experienced a near-complete extinction of *Lbx *genes. However, previous *Lbx *gene assignments were sometimes based on limited sequence comparisons, and neither a comprehensive phylogenetic analysis nor a comparative genomic analysis of vertebrate *Lbx *genes had been carried out. Therefore, it remained unclear whether more *Lbx *genes had yet to be found and whether these genes were survivors of *Lbx1, 2, 3 *or *4 *genes.

In this study, we searched vertebrate sequence databases for *Lbx *genes and determined their phylogenetic relationships. Moreover, we investigated the organisation of *Lbx *gene loci, identified signature genes linked to these loci and established the phylogenetic relationships of these genes. Our study confirms the existence of two *Lbx *genes in amniotes, while only one *Lbx *gene was found, thus far, in *Xenopus tropicalis*. However, we were able to detect novel *Lbx *genes in teleosts. Yet, we found no species with the predicted set of four (lobe-finned fish lineage) or eight (ray-finned fish lineage) *Lbx *genes. Thus, in all bony vertebrates, some *Lbx *genes have been lost. Nevertheless, tracing the signature genes linked to different *Lbx *loci, we were able to identify remnants of all four *Lbx *paralogons in tetrapods and all eight *Lbx *paralogons in teleosts. However, only *Lbx1 *and *Lbx2*-type loci retained *Lbx *genes; no evidence for surviving *Lbx3 *or *Lbx4 *genes was found. With cognate tetrapod and fish paralogons displaying a high degree of similarity, this suggests that *Lbx3 *and *4 *genes were lost before the split of the lobe-finned and ray-finned fish lineages. Finally, our phylogenetic and synteny analyses revealed that *Lbx*-associated genes in *Lbx1-Lbx4 *paralogons and in *Lbx2-Lbx3 *paralogons, respectively, are more closely related. This suggests that *Lbx1 *and *Lbx4 *paralogons originated from one, and *Lbx2 *and *Lbx3 *paralogons from the other, parental paralogon generated during the first round of vertebrate genome duplication.

### Two novel *Lbx *genes were identified in teleosts

Our searches of genomic databases identified two novel *Lbx *genes in zebrafish, and three *Lbx *genes in *Takifugu rubripes *(fugu), *Tetraodon nigroviridis *and *Gasterosteus aculeatus *(stickleback). *Oryzias latipes *(medaka) carries two *Lbx *genes, with a sequence gap at the expected position of the third. Taken together, this suggests that teleosts share the same set of 3 *Lbx *genes.

Phylogenetic analyses grouped the two novel zebrafish Lbx proteins, two of the three fugu, *Tetraodon *and stickleback proteins and one of the medaka proteins with the tetrapod Lbx1 sequences. Conversely, the zebrafish protein currently known as Lbx1, the remaining fugu, *Tetraodon*, stickleback and medaka sequences and the chicken protein so far called Lbx3 all grouped with the mammalian Lbx2 sequences. The bipartite separation of Lbx sequences was supported by high bootstrap values, suggesting that in all extant Osteichthyes, only *Lbx1 *and *Lbx2 *type genes were maintained, with teleosts harbouring duplicates of *Lbx1*.

### Traces of four *Lbx *loci were identified in tetrapods

Prior to the two rounds of vertebrate genome duplication, the *NK *clusters broke up and non-*NK *genes were acquired by these loci ([3] and this study). Our analysis shows that tetrapod *Lbx1 *is invariantly linked to *Tlx1, Kazald1, Btrc, Slc2a15, Poll, Dpcd, Fbxw4, Fgf8, Npm3, Mgea5, Kcnip2, Ldb, Loxl4 *and *Slit1 *with *Slc2a15 *being lost in mammals. The assignment of *Lbx2*-type genes was more problematic, due to incomplete sequence information for chicken and frog. For mammals however, *Lbx2 *was found linked to *Tlx2*, *Pcgf1*, *Aup1, Loxl3 *and *Dok1*, and linked *Loxl *and *Aup *genes were also identified for chicken (distribution of signature genes for Lbx loci shown in Table 1).

**Table 1:** Distribution of paralogous genes at the four Lbx-Tlx loci.

	Lbx1/Tlx1 locus	Former Lbx4/Tlx4 locus	(Former Lbx3)/Tlx3 locus	Lbx2/Tlx2 locus	Lbx-less Nkx3.2 locus	Lbx2/Tlx2 loci
				amniotes	tetrapods	teleosts
Msx			Msx2			
Nkx2/tin	Nkx2.3	Nkx2.6	Nkx2.5			
Nkx3/bap		Nkx3.1			Nkx3.2	Nkx3.2
		(chicken: Nkx2.8 only)				
Nkx6		Nkx6.3				
Lbx	Lbx1			Lbx2		Lbx2
Tlx	Tlx1		**Tlx3**	**Tlx2**		Tlx2
Pol	Poll	Polb				
Dpcd	Dpcd					
Fbxw4	Fbxw4					
Fbxw7					Fbxw7	Fbxw7
Slc2a15	Slc2a15				Slc2a9 (tetrapods)	
Aup				Aup1		Aup1
Pcgf1				Pcgf1		Pcgf1
Fbxw11/Btrc	Btrc		Fbxw11/Btrc2			
Mgea	Mgea5			Mgea2 (chicken)		Mgea2
Kazald	Kazald1 (tetrapods)	Kazald3 (fish)		Kazald2 (zebrafish)		
Ldb	Ldb1				Ldb2	Ldb2
Prom	Prom3 (fish)	Prom2 (tetrapods)*			Prom1	Prom1
Loxl	Loxl4	Loxl2		Loxl3		Loxl3
Slit	Slit1		Slit3		Slit2	Slit2
Dok		Dok2	Dok3	Dok1		Dok1
Fgf	**Fgf8**	**Fgf17**	Fgf18			Fgf24
Npm	Npm3	Npm2	**Npm1**			**Npm4**
Kcnip	Kcnip2	Kcnip3*	**Kcnip1**		**Kcnip4**	**Kcnip4**

* in human, cattle, dog, Prom2-Kcnip3 are on the same chromosome but at a distance to *Lbx2.*

Note, genes occurring at single *Lbx/Tlx *loci form monophyletic trees, i.e. the loci in fish and tetrapods are related (see additional file 2). Genes at more than one locus: gene phylogeny matches genomic localisation and environment (exception: *Dok2 *genes which are distinct from *Dok1 *but do not group well; see additional file 2). Genes at the putative *Lbx4-Tlx4 *locus, characterised amongst others by *Fgf17-Npm2*, match between fish and tetrapods. Genes found associated with *Lbx2 *loci in fish match genes in amniotes distributed between the *Lbx2 *locus and the *Lbx*-free *Nkx3.2 *locus, suggesting that the amniote *Lbx2 *locus once belonged to the *Nkx3.2 *containing *Nk *cluster. Genes in bold: genes that supported by high bootstrap values are more related to each other than to other paralogues, suggesting that the *Lbx1 *and *Lbx4 *loci evolved from one and the *Lbx2 *and *Lbx3 *from the other of the paralogons generated during the first round of vertebrate genome duplication.

The linking of *Lbx *with *Tlx *genes was not unexpected, given its maintenance in the otherwise fragmented amphioxus *nk *cluster [3]. Yet tetrapods harbour a third *Tlx *gene, suggesting that a third, previously *Lbx3*-containing paralogon still exists [39-41]. Indeed, tetrapod Tlx3 sequences form a phylogenetic group distinct from Tlx1 and Tlx2 sequences, supporting the idea that this gene did not arise from a single gene duplication event. Moreover, *Tlx3 *loci share the same organisation amongst tetrapods and encompass *Btrc2, Fgf18, Npm1, Kcnip1*, and *Slit3*, which according to our phylogenetic analysis are paralogues of the genes found at the *Lbx1 *site. Thus, while *Lbx3 *itself was lost from the *Tlx3 *locus, a number of genes that must have been acquired by the *Nk *cluster prior to the two rounds of vertebrate genome duplication are still present.

Using the previously identified signature genes linked to the *Lbx/Tlx *loci as query sequences, we also identified remnants of the fourth *Lbx *paralogon. Interestingly, both the *Lbx1/Tlx1 *and the *Tlx3 *locus contain closely linked *Fgf-Npm *genes, and an additional pair of tightly linked *Fgf-Npm *genes, namely *Fgf17 *and *Npm2*, was found in mammals, linked to *Dok2*, *Loxl2*, *Nkx3.1 *and *2.6*. Linked *Loxl2-Dok2-Npm2 *sequences were also found in the chicken genome, and in the frog, linked *Dok2-Npm2 *genes and *Loxl2-Nkx3.1-Nkx2.6 *genes were found on two, possibly neighbouring scaffolds. In some tetrapods the *Loxl2-Nkx3.1-Nkx2.6 *genes were also linked to *Prom2 *and *Kcnip3*. This suggests that *Prom2-Kcnip3 *initially belonged to the fourth *Lbx *paralogon that contains *Fgf17-Npm2 *as well as *Dok2-Loxl2-Nkx3.1-Nkx2.6 *genes, while the placement of *Prom2-Kcnip3 *on the same chromosome but at a distance to *Lbx2 *in humans, cattle and dog (but not mouse) is probably a result of a more recent transposition event. In summary, presence of a number of signature genes for *Lbx *loci linked to *Nkx2.6 *and *Nkx3.1 *suggests that this region is the ancestral home of the lost *Lbx4-Tlx4 *genes.

### Duplicates of the four tetrapod-type *Lbx *loci were identified in teleosts, with two *Lbx1 *genes and one *Lbx2 *gene still present

In teleosts, the two novel, putative *Lbx1 *genes were found in two types of genomic settings, both of which contained signature genes for the tetrapod *Lbx1 *locus. Genes that occurred at only one of the novel teleost *Lbx *sites formed monophyletic groups with their tetrapod counterparts. Importantly, *Slc2a15 *and *Fgf8 *genes, which were found at both of the teleost loci as well as the tetrapod *Lbx1 *sites, also formed single groups, indicating that the two novel *Lbx *loci in teleosts arose from a common *Lbx1*-containing ancestor. Consequently, tetrapod *Lbx1 *genes and the two novel teleost *Lbx *genes are orthologs.

Similar to the *Lbx1 *paralogons, the relationship of the now *Lbx*-less *Tlx3 *loci could readily be established. Teleosts harbour one locus containing *Slit3, Kcnip1, Tlx3, Fgf18 *and *Btrc2 *genes in the same order as found at the tetrapod *Tlx3 *site, with the exception of *Tetraodon *whose *Slit3 *gene intercalated between *Kcnip1 *and *Fgf18*. Notably, this *Tlx3 *locus is found on the same chromosome, but with different integration sites, as the gene we propose to rename *Lbx2*, suggesting extensive transposition between *Lbx/Tlx *loci in teleosts. The second *Tlx3 *locus contains *Kcnip1*-*Npm1 *and in zebrafish also *Tlx3b*, plus a second copy of *Fgf18 *and *Btrc2*. Further shared genes were found in the environment of the tetrapod and teleost *Tlx3 *loci; genes at these sites, including the *Tlx *genes themselves, grouped together in our phylogenetic analyses. This suggests that teleost *Tlx3 *and *Tlx3b *are orthologs of the tetrapod *Tlx3 *genes. The high degree of locus conservation also suggests that the basic lay out of this *Tlx3 *paralogon, including the elimination of *Lbx3*, was established prior to the teleost-specific genome duplication.

In the zebrafish, the gene currently known as *lbx1 *but placed with mammalian *Lbx2 *in our phylogenetic analyses was found in a genomic environment bearing the hallmarks of the mammalian *Lbx2 *locus, being linked with *Pcgf1*, a *Tlx *gene currently named *Tlx3a, Aup1 *and *Loxl3*. These genes formed monophyletic groups with the cognate genes at the mammalian *Lbx2 *locus, with teleost *Tlx3a *grouping with mammalian *Tlx2*. This suggests that this locus is indeed a *Lbx2 *paralogon. In fugu, *Tetraodon*, stickleback and medaka, the third *Lbx *gene was also linked to *Pcgf1 *and a *Tlx2*-type gene, although *Aup *was located at a distance or in case of *Tetraodon*, on a different chromosome. The latter seems to be a result of a secondary rearrangement of the locus, as *aup *is linked to *lbx *in amphioxus and hence, was part of *lbx *loci before the split of cephalochordate-vertebrate lineages (*aup *and *lbx *are both found on Scaffold 294 of the Branchiostoma floridae v1.0 genome at the JGI. See Availability and requirements section for URL). Nevertheless, all of the teleost *Lbx2 *loci encompassed a *Fgf24-Npm4 *set between *Pcgf1 *and *Tlx2*. Moreover, genes associated with *Prom1 *and directly linked to fugu, *Tetraodon*, stickleback and medaka *Lbx2 *were found in the same order in the wider environment of the zebrafish *Lbx-Pcgf1-Tlx *set. Phylogenetic analyses indicated that these genes are orthologs, suggesting that the teleost *Lbx2 *loci are variations on the same theme.

In fugu, *Tetraodon*, stickleback and medaka, the *Lbx2 *gene was directly adjacent to *Nanos1*, which is not linked to the mammalian *Lbx2 *gene. In mammals on the other side, the *Lbx2*-*Pcgf1-Tlx2 *set was flanked by *Ttc31 *and *Loxl3-Dok1-Np620159.2*-*Sema4f*. Yet in these four teleosts, a second *Nanos1 *gene was identified, linked to *Loxl3, Dok1, Np620159.2, Sema4f *and *Ttc31*. Moreover, a duplicate of the *Prom1*-linked gene set was found at the same site. A similar arrangement of genes was also detected in the zebrafish, distributed however between two chromosomes and with individual genes missing from the set. Phylogenetic analyses grouped the genes with the cognate genes at the mammalian and teleost *Lbx2*-bearing sites, suggesting that this location is the second, now *Lbx2/Tlx2*-less *Lbx2 *paralogon in teleosts. As will become relevant below, this locus encompassed *Slit2*, *Kcnip4 *and *Fbxw7*, associated with the *Lbx*-free *Nkx3.2 *locus in tetrapods.

Establishing whether teleosts carry duplicates of the possible fourth *Lbx *paralogon was more problematic as unfortunately, some of the genes suggestive of this paralogon (namely *Fgf17, Npm2, Dok2, Loxl2, Prom2, Kcnip3) *reside on rather small scaffolds and hence, linkage could not always be established. However, all five teleosts harboured two sets of *Loxl2-Kcnip3 *genes. One of the sets was associated with *Dok2 *and in stickleback, also with *Fgf17*. Moreover, *Npm2*-*Dok2*-*Fgf17 *were found together on zebrafish chromosome 8. Phylogenetic analyses grouped *Fgf17, Npm2, Loxl2, Kcnip3 *genes with their namesakes from tetrapod genomes. This suggests that, although less well preserved, two copies of the putative fourth *Lbx *paralogon still exist in extant teleosts.

### The mammalian *Lbx2 *locus has translocated from the *NK *cluster containing *Nkx3.2*

Previous studies of human *Nk *genes showed that remnants of two *Nk *clusters have been preserved, with chromosome 10 carrying *LBX1 *and *TLX1 *linked to *NKX2.3 *and *NKX1.2*, and chromosome 5 carrying *TLX3 *linked to *NKX2.5 *[3]. More partial remnants of two further *Nk *clusters have also been identified, one on chromosome 8 with *NKX2.6 *and *NKX3.1 *and one on chromosome 4 with *NKX3.2 *and *NKX1.1*. However, no *LBX *or *TLX *genes are linked to these regions.

A previous study, based primarily on the human genome, proposed that the *Nkx2.6, 3.1 *and *6.3 *paralogon (located on human chromosome 8) might be the ancestral location of *Lbx2 *and *Tlx2 *(now located on human chromosome 2) [3]. By a process of elimination, this would further suggest that *Lbx4 *and *Tlx4 *were lost from the *NKX3.2 *paralogon (currently located on human chromosome 4). Consistent with this hypothesis, paralogs of a few genes (*Kcnip*, *Prom*, *Add*, *Adra2*) are represented at both the *Lbx2 *and the *Nk3.2 *loci in mammals, suggesting that these two loci have derived from separate *Lbx/Tlx *paralogons. However, phylogenetic analysis of these gene families shows that the mammalian genes linked to the *Lbx2 *loci group with frog, chicken and/or teleost genes associated with *Fgf17 *and *Npm2 *loci (see phylogenetic analysis and discussion in Additional file 2). This suggests that these genes may have translocated to the mammalian *Lbx2 *loci from the putative 4^th ^*Lbx/Tlx *paralogon (e.g. the genes now on human chromosome 2 may have originally been linked to the *Tlx/Lbx *paralogon now on human chromosome 8).

Consistent with this second interpretation our data suggests that the ancestral location of the *LBX2/TLX2 *paralogon now located on human chromosome 2 was the *NKX3.2 *paralogon on human chromosome 4 (as shown in Fig. 3). The evidence for this is several fold. Firstly, we have identified four distinct *Fgf *and *Npm *genes, with *Fgf8-Npm3 *demarcating *Lbx1/Tlx1 *loci, *Fgf18-Npm1 *the *Tlx3 *loci, *Fgf24-Npm4 *the preserved teleost *Lbx2/Tlx2 *locus and *Fgf17-Npm2 *genes associated with tetrapod and teleost *Nkx2.6-3.1*-*6.3 *genes. Secondly, paralogous *Loxl *and *Dok *genes are present at both the *Lbx2 *locus and the *Nkx2.6-3.1-6.3 *locus, suggesting that these two loci have evolved from different paralogons. Thirdly, amniote orthologs of genes associated with the two teleost *Lbx2 *loci such as *Fbxw7, Ldb2, Prom1, Slit1 *and *Kcnip4 *co-localise with *Nkx3.2*, and these amniote and teleost genes fall into the same phylogenetic groups. Fourthly, the *Lbx2 *locus is still associated with *Nkx3.2 *genes in teleosts, and possibly, also in the chicken (the signature genes linked to the *Lbx2 *locus are assigned to chromosome 4, but their position is not determined). All together, this strongly suggests that the mammalian *Lbx2-Tlx2 *locus was once located in the *Nk *cluster containing *Nkx3.2 *and that *Lbx4 *and *Tlx4 *were lost from the *Nkx2.6-3.1-6.3 *paralogon that contains *fgf17 *and *npm2*.

### *Lbx4*-*Tlx4 *were lost prior to the third genome duplication in ray-finned fish

If the assignment of *Lbx2-Tlx2 *to the *Nkx3.2 *carrying *Nk *cluster is correct then, as discussed above, the *Nk *cluster containing *Nkx2.6, 3.1 *and *6.3 *(and *fgf17 *and *npm2*) is the former location of the *Lbx4-Tlx4 *paralogon. This idea is supported by the presence of a number of signature genes for *Lbx *loci at this site. Moreover, genes at this locus do not group with paralogues accompanying other *Lbx *loci; most notably, the *Fgf-Npm *and *Kcnip *genes located here constitute distinct phylogenetic groups. Taken together, this suggests that different to *Lbx2-Tlx2 *genes, *Lbx4-Tlx4 *genes did not translocate to another chromosome. Rather, they were lost from this fourth *NK *cluster. Significantly, both teleosts and tetrapods retained a similar set of genes from this locus. This indicates that the basic lay-out of the paralogon, including the elimination of its *Lbx *and *Tlx *genes, was probably established in an ancestor of the Osteichthyes (i.e. prior to the split of the lobe-finned and ray-finned fish lineages).

### *Lbx *loci in extant vertebrates arose from *Lbx1/4 *and *Lbx2/3 *precursors

Our study shows that of the genes associated with *Lbx-Tlx *loci, *Fgf*, *Npm *and *Kcnip *genes still exist in four copies, inferring that indeed, the original chordate locus was duplicated twice in the lineage leading to vertebrates. Phylogenetic analyses indicated that *Fgf8 *genes found in the *Lbx1/Tlx1 *paralogon and *Fgf17*, found in the *Lbx4/Tlx4 *paralogon, are more closely related than *Fgf24 *found in the intact teleost *Lbx2/Tlx2 *paralogon and *Fgf18 *found in the *Lbx3/Tlx3 *paralogon. Similarly, *Npm4 *(teleost *Lbx2/Tlx2 *paralogon) and *Npm1 *(*Lbx3/Tlx3 *paralogon) are closely related to each other, distinguished from *Npm1 *(Tlx3 paralogon) and *Npm4 *(teleost *Lbx2 *paralogon), A similar grouping was found for *Kcnip *genes, with *Kcnip1 *(Tlx3 paralogon) and *Kcnip4 *(teleost Lbx2 and tetrapod Nkx3.2 loci) being the most closely related. This pair-wise grouping is supported by high bootstrap values and suggests that during the second vertebrate genome duplication, *Lbx1 *and *Lbx4 *paralogons arose from one of the *Lbx-Tlx *loci generated by the initial duplication event and *Lbx2 *and *Lbx3 *paralogons arose from the other duplicated locus. Consistent with this model, *Tlx2 *and *Tlx3 *genes are rather similar and are easily distinguished from the *Tlx1 *genes associated with *Lbx1*.

### Basic roles of *Lbx1 *genes and neofunctionalisation of mammalian *Lbx2 *genes

Our analysis shows that the organisation of orthologous *Lbx/Tlx *paralogons is very similar in extant bony vertebrates, suggesting that the main compositions of the paralogons were established before the divergence of lobe-finned and ray-finned fish. This infers that Osteichthyes had only two types of *Lbx *genes. Expression analyses and functional studies on *Lbx1 *type genes suggest that they all play a role in dorsal spinal cord and muscle development [6-16]. Interestingly, the zebrafish and chicken *Lbx2 *genes are still expressed in muscle precursors, and zebrafish *lbx2 *is also expressed in the spinal cord [14,18]. In contrast, mouse *Lbx2 *is not expressed in these regions, rather labelling the urogenital system, eye and brain [19]. Amniote Lbx2 proteins also show the most divergent sequences. This suggests that amniote (or potentially sarcopterygian) *Lbx2 *genes have evolved at a faster rate than their anamniote (or non-sarcopterygian) orthologs, and undergone neofunctionalisation. Other *Lbx2 *genes, however, may have retained aspects of ancestral *Lbx *expression and function, which seems to be tied to the formation of specific neural and muscle cell types.

## Conclusion

**Figure 4:**
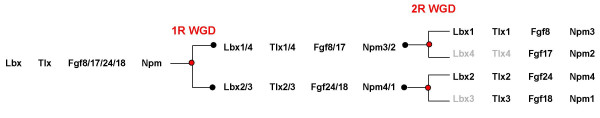
**A model of the evolution of the vertebrate *Lbx/Tlx *loci**. *Lbx *loci in extant vertebrates arose from a region containing an *Lbx*, *Tlx*, *Fgf8/17/24/18 *and an *Npm *gene. After one round of whole genome duplication (1R WGD) *Lbx1/4 *and *Lbx2/3 *precursors were produced linked to *Fgf8/17 – Npm2/3 *and *Fgf24/18 – Npm4/1 *precursors, respectively. During the second round of whole genome duplication (2R WGD), four loci where produced. By the time of the divergence of the lobed-fined and ray-finned fish, the *Lbx4*, *Tlx4 *and *Lbx3 *genes (shown in grey) had been lost.

We have identified remnants of all four *Lbx/Tlx *paralogons in extant bony vertebrates. Phylogenetic analyses of *Lbx*, *Tlx *and non-*NK *genes at these loci revealed that the first round of whole genome duplication in vertebrates created the ancestor of the *Lbx1/Tlx1 *and *Lbx4/Tlx4 *paralogons and the ancestor of the *Lbx2/Tlx2 *and *Lbx3/Tlx3 *paralogons (Fig. 4). After the second genome duplication, *Lbx *and *Tlx *genes were lost, such that before the split of the ray-finned fish/teleost and lobe-finned fish/tetrapod lineages only *Lbx1/Tlx1*, *Lbx2/Tlx2 *and *Tlx3 *genes were maintained. In the ray-finned fish lineage, genome duplication and subsequent gene loss left teleosts with two *Lbx1*, one *Lbx2 *and two *Tlx3 *loci. Since the amniote *Lbx2 *genes show divergent amino acid sequences and expression patterns, we propose that these *Lbx2 *genes have been evolving at a faster rate and were subject to neofunctionalisation. *Lbx1 *genes on the other hand may have retained more features of the original chordate *lbx *gene.

## Methods

Regions surrounding the human *LBX/TLX *cluster loci were searched using NCBI Map Viewer build 36.2. To identify further paralagous genes, BLAST searches with putative protein sequences were conducted against the human genome, and putative positive targets were further characterised by molecular phylogenetics to resolve orthologous and paralagous relationships. Each of the genes from the putative human paralogons was cross-referenced by BLAST against the following NCBI genome assemblies; *Mus musculus *(build 37.1), *Gallus gallus *(build 2.1) and *Danio rerio *(Zv6) on NCBI map viewer. The *Xenopus tropicalis *(v4.1) and fugu *rubripes *(v4.0) genomes were searched at the Joint Genome Institute genome portal, and the *Tetraodon nigroviridis *(v8), *Oryzias latipes *(*MEDAKA1*) and *Gasterosteus aculeatus *(BROAD S1) genomes were searched via the Ensembl server. Putative target sequences were further analysed by molecular phylogenetics, to establish orthologous and paralogous relationships and to reaffirm our original classification of human sequences. For molecular phylogenetic analyses, protein sequences were first aligned, (with invertebrate outgroup sequences included where possible) using ClustalX and edited by eye [42]. Phylogenetic analyses were then carried out using maximum likelihood implemented by ClustalX [42], and by PHYML [43,44].

## Availability and requirements

JGI: http://genome.jgi-psf.org/Brafl1/Brafl1.home.html

## Authors' contributions

SD and KL conceived of the study, KW and FW performed the phylogenetic analyses, KW, FW, KL and SD equally contributed to the genomic searches, identification and characterisation of Lbx loci, and to the design of the manuscript. All authors read and approved the final manuscript.

## Supplementary Material

Additional file 1 Genomic arrangement of the Lbx paralogons. Click here for file

Additional file 2 **Description of gene families co-localising with *Lbx/Tlx *loci, and their phylogenetic analysis**. Also included in this file are a list of abbreviations, the accession numbers of each sequence and a suggested new nomenclature for some of the genes. Click here for file
