# Supplementary material for: Comparative genomics of Lbx loci reveals conservation of identical Lbx ohnologs in bony vertebrates
Source: BMC Evol Biol. 2008 Jun 9;8:171. doi: 10.1186/1471-2148-8-171 (PMC2446394; doi:10.1186/1471-2148-8-171)
Supplement: Additional file 1 — Genomic arrangement of the Lbx paralogons. [file 1471-2148-8-171-S1.ppt]

## Slide 1
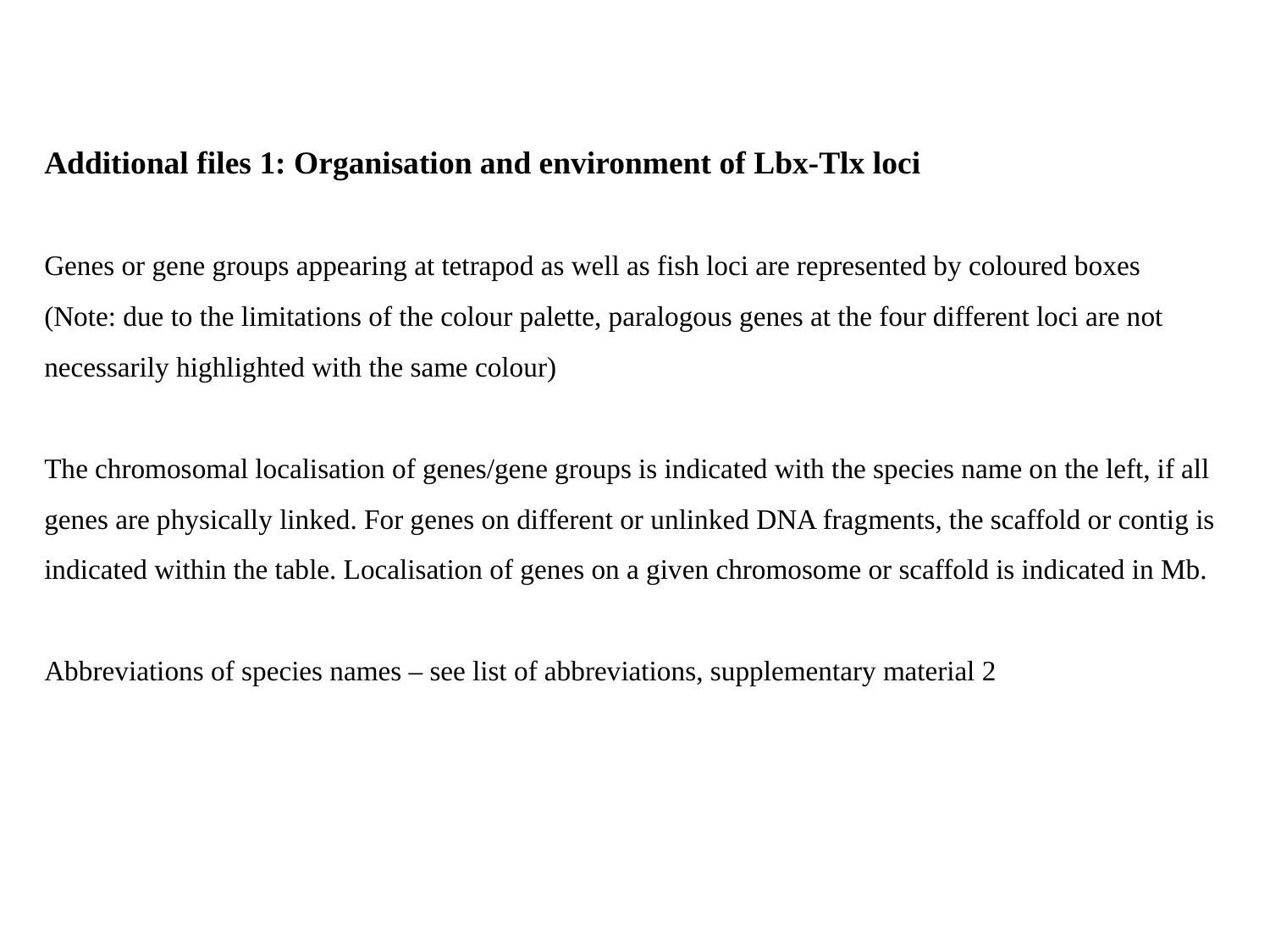

Additional files 1: Organisation and environment of Lbx-Tlx loci
Genes or gene groups appearing at tetrapod as well as fish loci are represented by coloured boxes
(Note: due to the limitations of the colour palette, paralogous genes at the four different loci are not
necessarily highlighted with the same colour)
The chromosomal localisation of genes/gene groups is indicated with the species name on the left, if all
genes are physically linked. For genes on different or unlinked DNA fragments, the scaffold or contig is
indicated within the table. Localisation of genes on a given chromosome or scaffold is indicated in Mb.
Abbreviations of species names – see list of abbreviations, supplementary material 2

## Slide 2
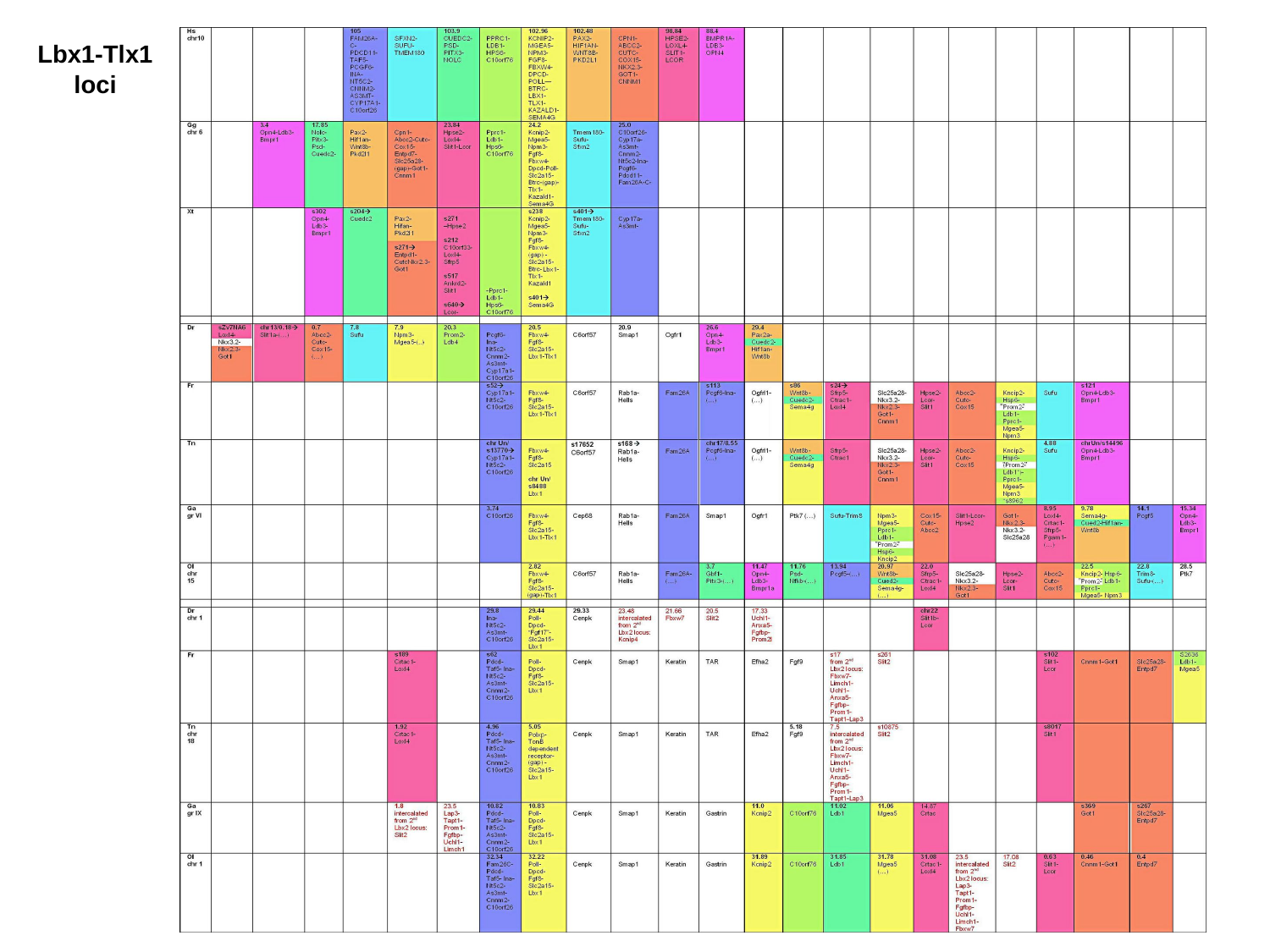

Lbx1-Tlx1
loci

## Slide 3
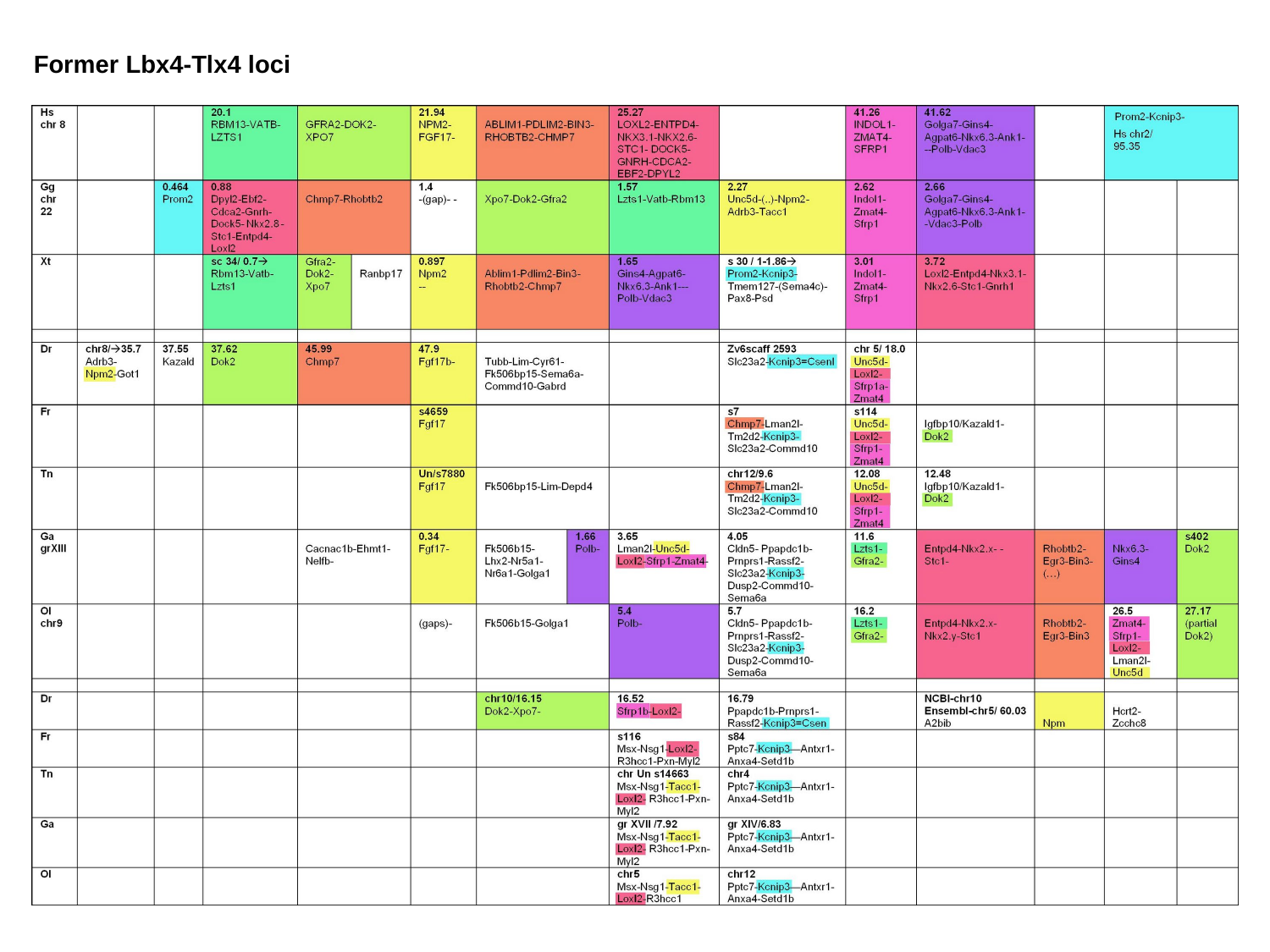

Former Lbx4-Tlx4 loci

## Slide 4
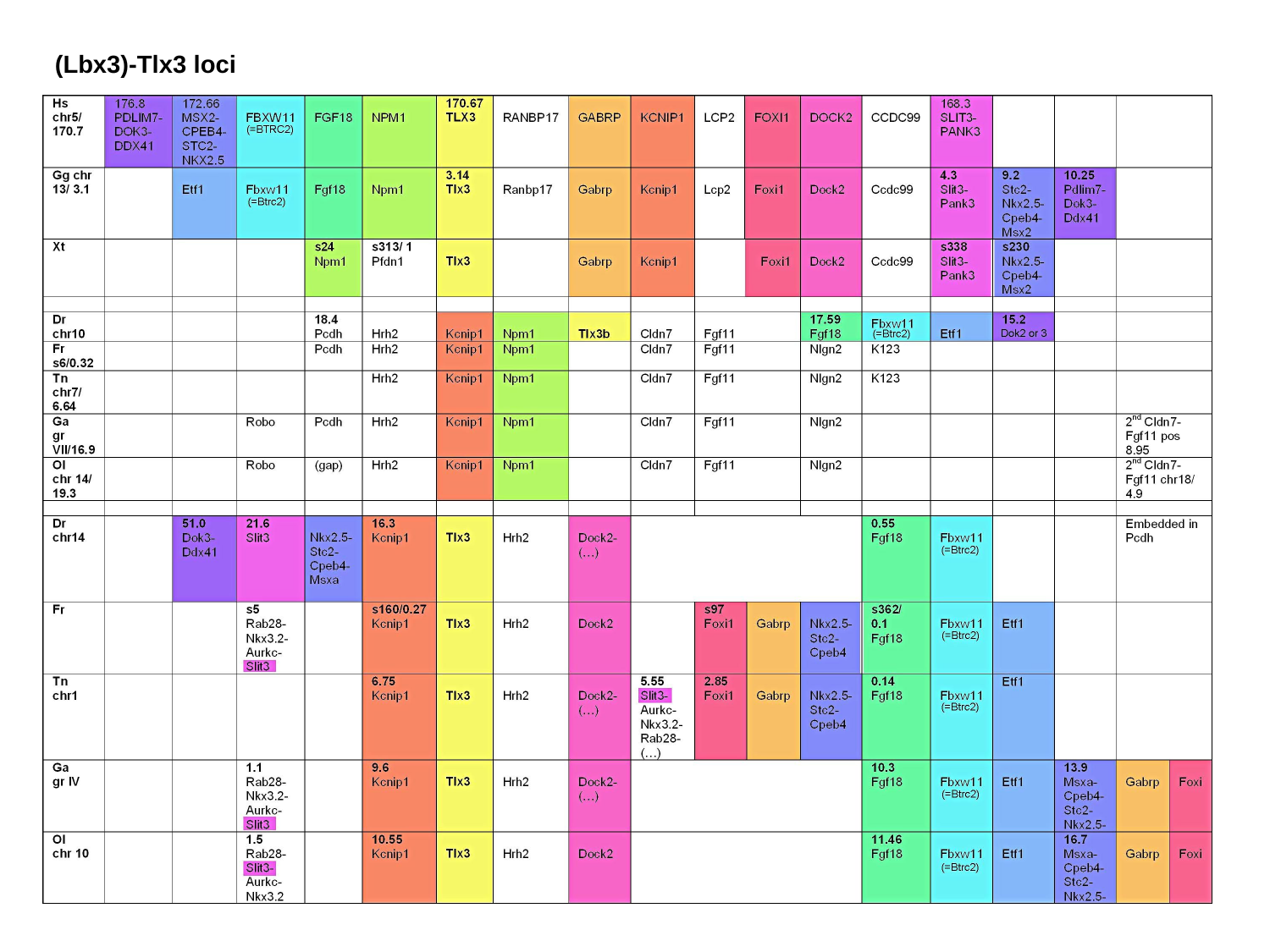

(Lbx3)-Tlx3 loci

## Slide 5
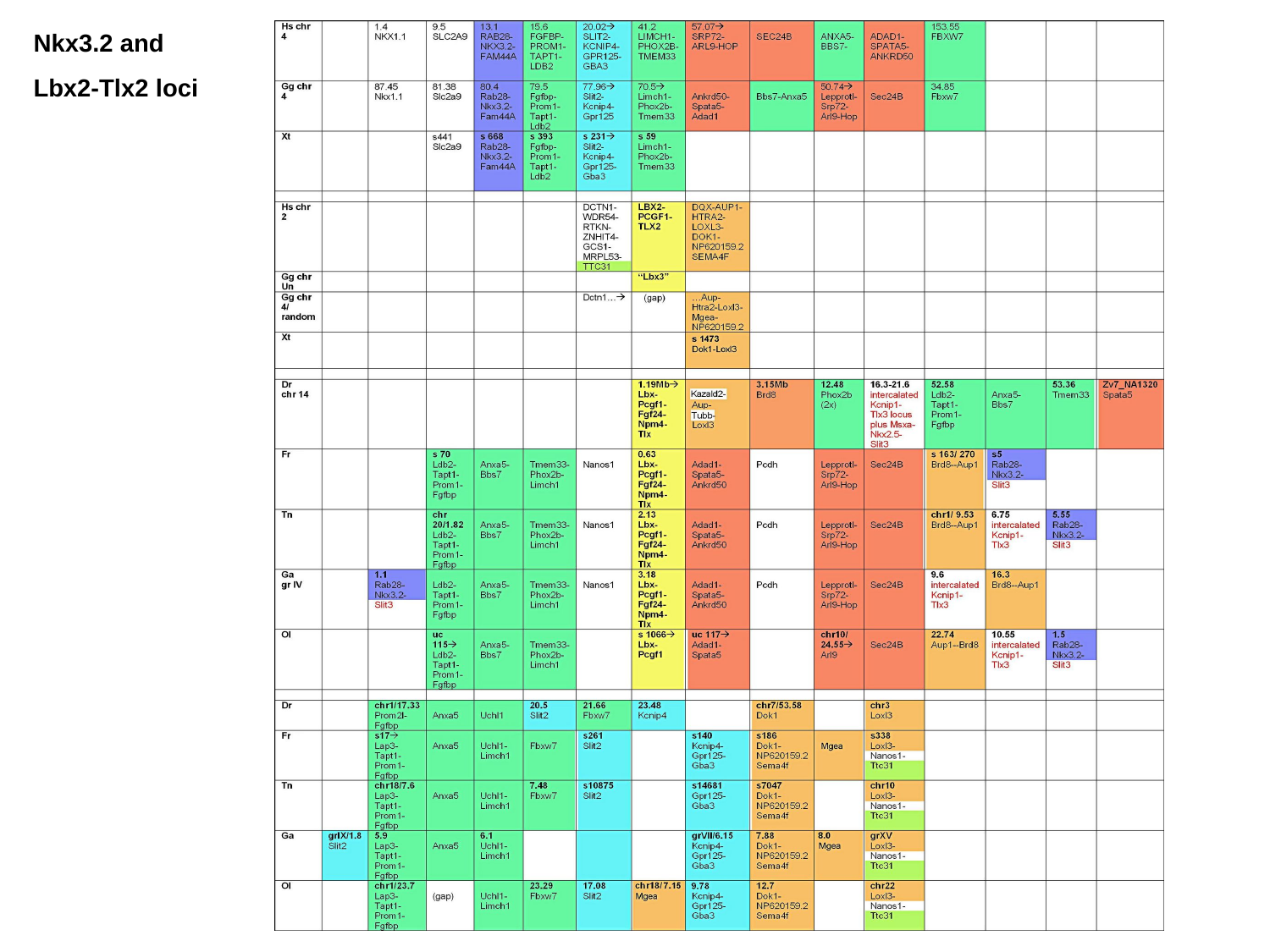

Nkx3.2 and
Lbx2-Tlx2 loci
