# Supplementary material for: Comparative genomics of Lbx loci reveals conservation of identical Lbx ohnologs in bony vertebrates
Source: BMC Evol Biol. 2008 Jun 9;8:171. doi: 10.1186/1471-2148-8-171 (PMC2446394; doi:10.1186/1471-2148-8-171)
Supplement: Additional file 2 — Description of gene families co-localising with Lbx/Tlx loci, and their phylogenetic analysis. Also included in this file are a list of abbreviations, the accession numbers of each sequence and a suggested new nomenclature for some of the genes. [file 1471-2148-8-171-S2.pdf]

Additional files 2 – Gene family introduction, sequence identification and phylogenetic analyses

<u>List of abbreviations</u>

Ae Aedes aegypti - **Yellow fever mosquito**
Ag Anopheles gambiae - **Mosquito**
Am Apis mellifera - **Honey Bee**
Bf Branchiostoma floridae - **Amphioxus**
Bt Bos taurus - **Cow**
Ci Ciona intestinali -. **Sea squirt**
Cf Canis familiaris - **Dog**
Dm Drosophila melanogaster - **Fruit fly**
Dr Danio rerio -**Zebrafish**
Fr Takifugu rubripes - **Japanese pufferfish, Fugu**
Ga Gasterosteus aculeatus - **Stickleback**
Gg Gallus gallus - **Chicken**
Hs Homo sapiens - **Human**
Le Leucoraja erinacea - **Little ray**
Md Monodelphis domestica - **Opossum**
Mm Mus musculus - **Mouse**
Nv Nematostella vectensis - **Sea anemone**
Oa Ornithorhynchus anatinus - **Platypus**
Ol Oryzias latipes - **Japanese killifish, Medaka**
Sp Strongylocentrotus purpuratus - **Purple sea urchin**
Tc Tribolium castaneum - **Red flour beetle**
Tn Tetraodon nigroviridis - **Green spotted pufferfish**
Xl Xenopus laevis - **African clawed frog**
Xt Xenopus tropicalis - **Pipid frog**

Below we give a brief introduction to each gene family discussed in the paper, followed by a summary of the molecular phylogenetic analysis and its interpretation. This is followed by a table giving accession numbers or other identifiers for the sequences used in these analyses.

Genes linked to one *Lbx* locus: *Poll, Dpcd, Fbxw4, Fbxw7, Slc2a15, Aup, Pcgf1*

Poll: DNA polymerase Lambda

(*Homo sapiens* chr10: POLL)

DNA polymerases are classified into four families A, B, X and Y [1]. DNA polymerase λ (POLL) is found on human chromosome 10 and is linked to *Lbx1* in the genomes of bony fish. It is a member of family X also including *Pol* β *and Pol μ,* each of which have orthologs in yeast and in the case of Poll, also in plants [1]. Members of this family are repair polymerases that have a 5'-deoxyribose-5-phosphate lyase activity. Poll is involved in base excision repair responsible for the repair of lesions that give rise to abasic sites in DNA. Poll has both DNA polymerase and terminal transferase activities [2]. Genome searches found only one copy of *Poll* in vertebrate genomes, associated with *Lbx1* genes. Maximum likelihood analysis grouped these genes together, distinguishing them from POLB linked to human NKX2.6 and 3.1 genes on chromosome 8.

Polymerase (DNA directed) lambda (Poll)

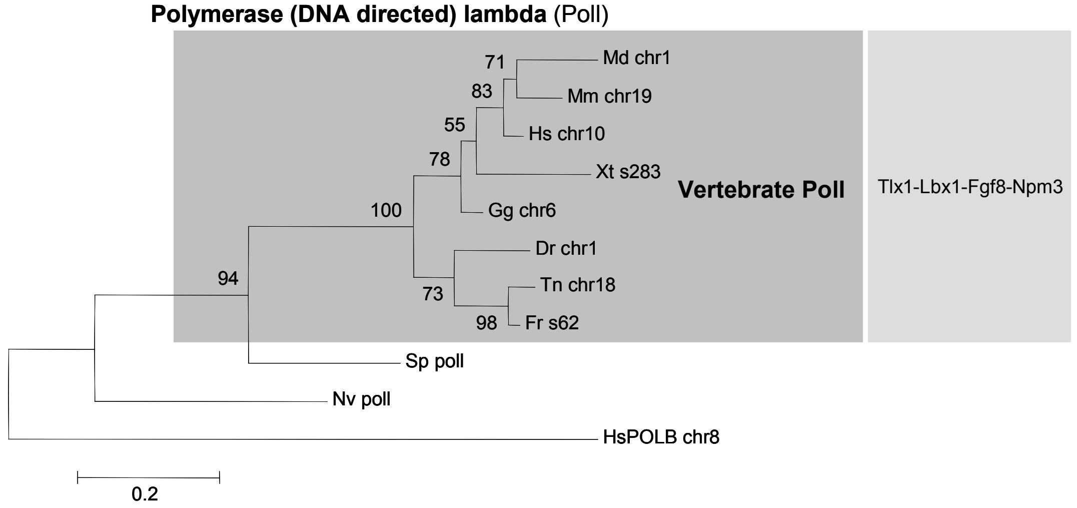

DPCD: Deleted in a mouse model of Primary Ciliary Dyskinesia

(*Homo sapiens* chr10: DPCD)

DPCD was found upon analysis of the genomic region surrounding the *POLL* gene, in a study of primary ciliary dyskinesia, an autosomal recessive disease caused by mutations that affect the function of cilia. *Dpcd* has a similar transcriptional start site as *Poll*, but is transcribed in the opposite direction and from the opposite strand [3]. The protein sequence of Dpcd encompasses approximately 203 amino acids and it is highly conserved in metazoans. Genome searches and maximum likelihood analysis identified invertebrate *dpcd* genes and a single vertebrate ortholog.

Deleted in a mouse model of primary ciliary dyskinesia (Dpcd)

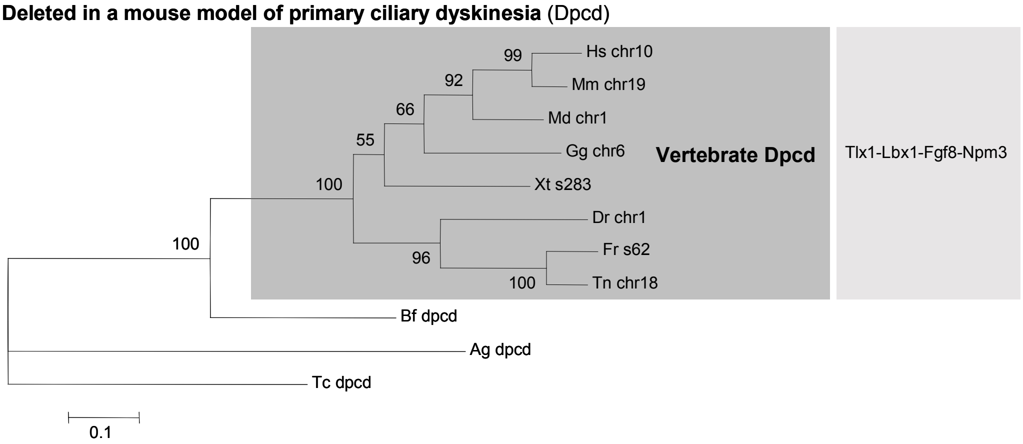

Fbxw4: F-box/WD repeat protein 4

(*Homo sapiens* chr10: FBXW4)

Fbxw proteins are thought to recognise and bind to selected phosphorylated proteins and promote their ubiquitination and degradation. Specifically, Fbxw4 proteins are thought to be involved in signalling pathways regulating limb development and they may participate in Wnt signalling [4]. Fbxw4 is expressed in brain, kidney, lung and liver, and defects in FBXW4 were shown to be a cause of split-hand/foot malformation type 3, an autosomal dominant disorder characterised by hypoplasia/aplasia of the central digits with fusion of the remaining digits. Genome searches and maximum likelihood analysis identified invertebrate *fbxw4* genes and a single vertebrate ortholog linked to *Lbx1* genes. Two further groups of *Fbxw* genes, namely *Fbxw7* and *Btrc/Fbxw11* genes, were investigated during this study. However, these genes and *Fbxw4* did not descend from a common ancestral bilaterian gene; the closest relative to FBXW4 in humans is FBXW9 (this work and [5]).

F-box and WD repeat domain containing 4 (Fbxw4/Dactylin)

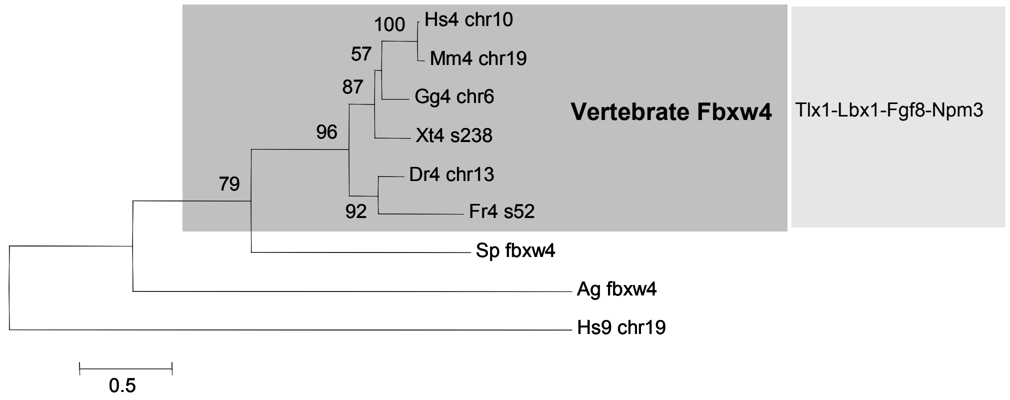

Fbxw7: F-box/WD repeat protein 7

(*Homo sapiens* chr4: FBXW7)

Genome searches identified a further F-box/WD repeat gene, Fbxw7. Maximum likelihood analysis identified invertebrate *fbxw7* genes and a single vertebrate ortholog, associated with the *Lbx*-less, *Nkx3.2* carrying loci in amniotes and the second, now dispersed *Lbx2* locus in teleosts. Fbxw7 sequences formed a monophyletic group.

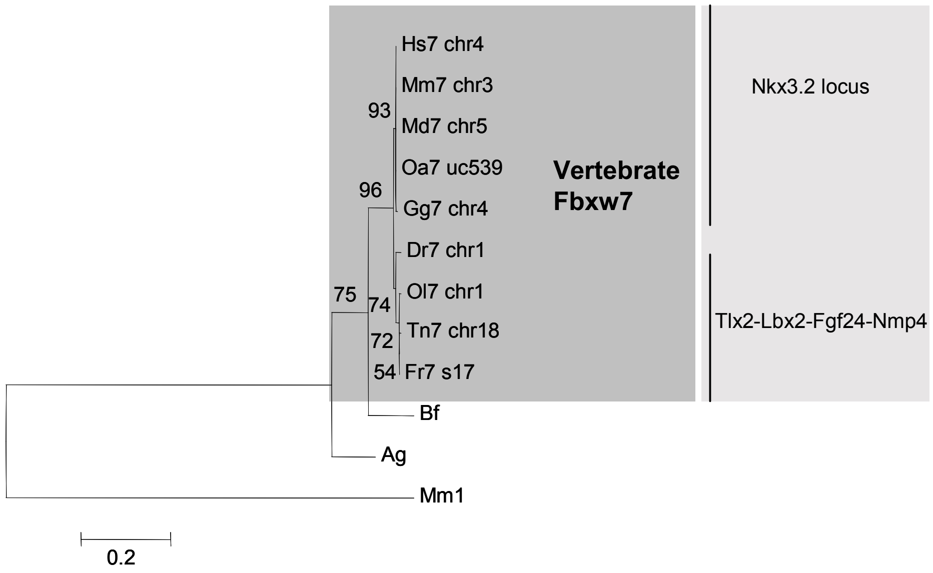

Slc2a: Solute carrier family 2a

(*Gallus gallus* chr6: Slc2a15)

The solute carriers currently comprise 43 protein families with 298 genes [6], of which *Slc2a15* (our name) was found linked with chicken, frog and teleost *Lbx1* genes. Slc2a15 belongs to the Slc2 family of glucose and polyol transporters with 14 known members, the glucose transporters Slc2A1-14 [7]. They are involved in the facilitated diffusion of glucose and related hexoses across biological membranes, and they all contain 12 transmembrane domains [7]. Previous studies have shown a close relationship between *Slc2a5/7/9* and *11* genes [7]. Here we extend the analysis by identifying a fifth member of this particular Slc2a subgroup named by us as *Slc2a15*, which has been lost in the lineage leading to mammals. Furthermore, we identified single invertebrate *slc2a5/7/9/11/15* genes from Amphioxus and insect genomes.

Maximum likelihood analysis confirms that there are five *Slc2a* orthologues in vertebrate genomes with *Slc2a15* being linked to the non-mammalian *Lbx1* loci. *Slc2a9* was found linked to amniote *Nkx3.2* loci, and *Slc2a11* and *Slc2a5/7* were not linked to *Lbx* or *Nk* loci. *Slc2a5/7* are next to each other in the genomes of humans and mouse, which along with the tree topology suggest they are tandem-duplicates. *Slc2a15* and *9* may form a subgroup within the *Slc2a5/7/6/11/15* genes. However, this is supported by mediocre bootstrap values.

Solute carrier family 2 (Slc2a)

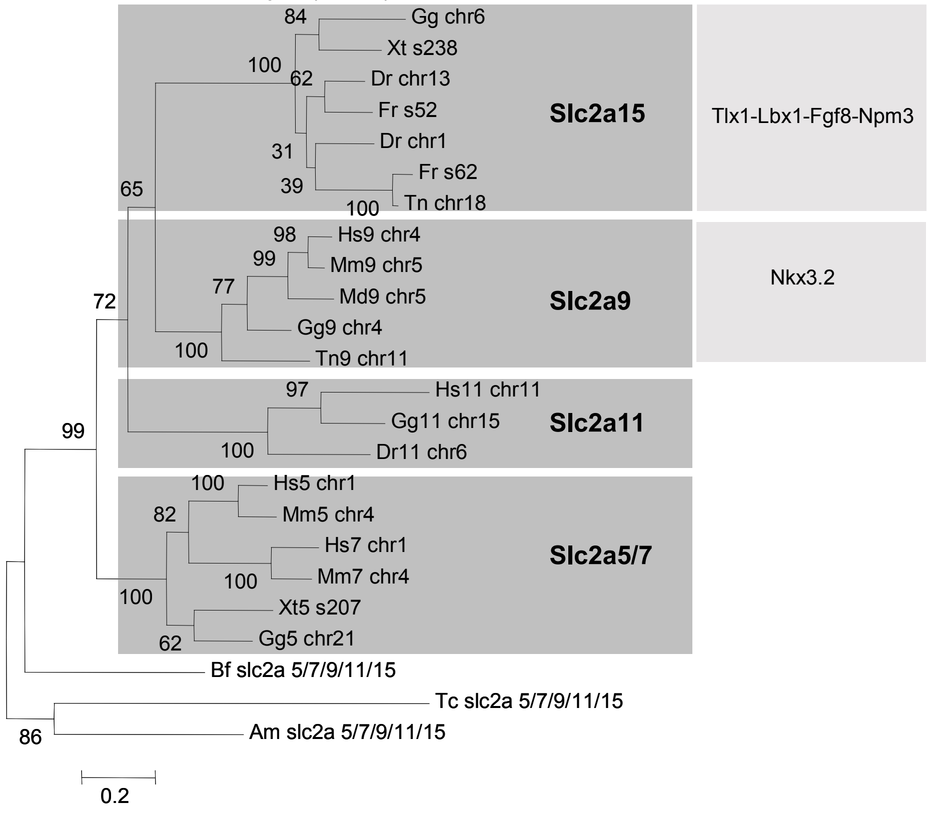

Aup1: Ancient ubiquitous protein

(*Homo sapiens* chr2: AUP1)

Human AUP1 is ubiquitously expressed, contains domains implicating a role in signal transduction and may be involved in Integrin signalling [8]. Searches of databases and phylogenetic analysis found only one vertebrate Aup1 linked to *Lbx2*. Various invertebrate metazoan orthologues were identified, including an ortholog from the slime mould *Dictyostelium discoideum*. Interestingly, *aup1* is located close to *lbx* in the genome of amphioxus. Phylogenetic analysis indicated that Aup sequences are highly related with vertebrate sequences grouping together.

Ancient ubiquitous protein (Aup)

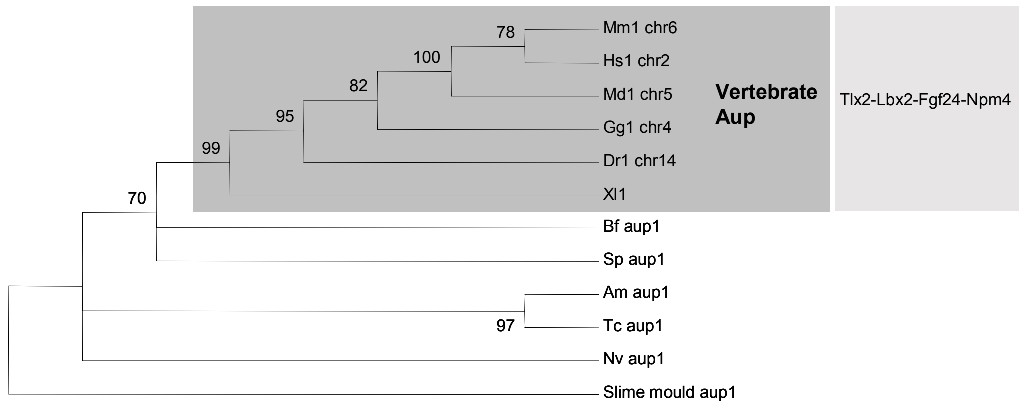

Pcgf: Polycomb group ring finger

(*Homo sapiens* chr2: PCGF1)

Members of the Pcgf1 family all contain one ring-type zinc finger and are components of polycomb group multiprotein complexes. These complexes are required to maintain the transcriptionally repressed state of certain genes. For example, Pcgf1 is a component of the Bcor multiprotein complex that maintains the transcriptionally repressed state of genes such as Bcl6 and Cdkn1A, the latter encoding a cyclin-dependent kinase inhibitor [9]. Genome searches and maximum likelihood analysis identified an invertebrate *pcgf1* gene from *Strongylocentrotus purpuratus* and a single vertebrate ortholog linked to *Lbx2* genes. Pcgf1 sequences grouped together, separated from representatives of other *Pcgf* genes families such as human PCGF3, 5 and 6.

Polycomb group ring finger 1 (Pcgf1)

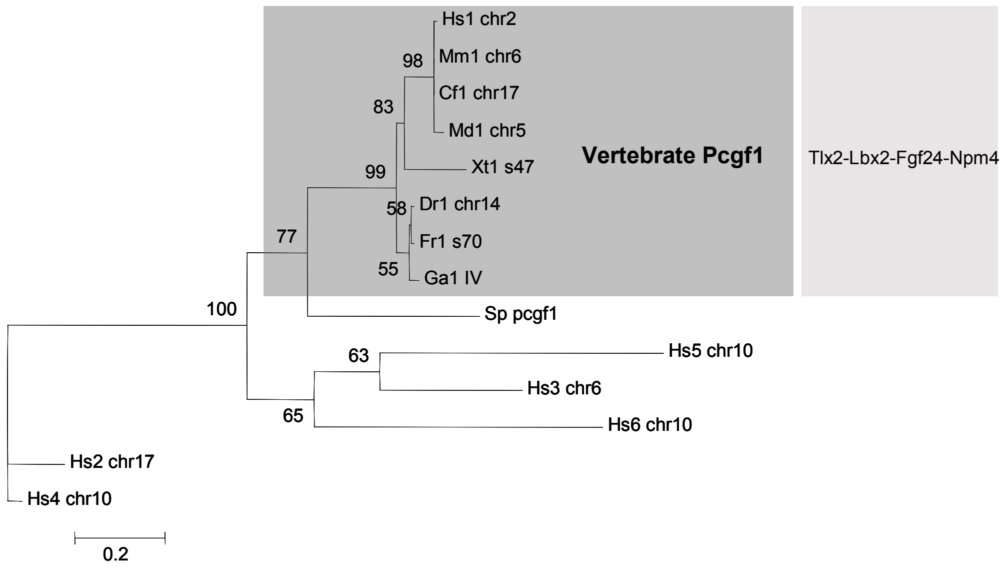

<u>Genes linked to two *Lbx* loci: *Lbx, Btrc/Fbxw11, Mgea, KazalD, Ldb, Prom*</u>

Lbx: Ladybird homeobox protein

(*Homo sapiens* chr10: LBX1, chr2: LBX2)

Lbx proteins are Nk-type homeodomain transcription factors; the genes are thought to have arisen from tandem duplications of an ancestral NK gene early in animal evolution [10]. *Lbx1* is required for the development of GABAergic interneurons in the dorsal horn of the spinal cord [11-14]. It is also implicated in the migration and further development of hypaxial muscle precursors for limb and tongue muscles [15-20]. In contrast, no function for *Lbx2* has been established [21]. The phylogenetic analyses of these sequences are presented in the main paper.

Btrc/Fbxw11: F-box/WD repeat proteins 1a and 11

(*Homo sapiens* chr10: BTRC, chr5: BTRC2/FBXW11)

Btrc/Fbxw11 proteins are a separate group within the F-box and WD repeat domain family as judged by the existence of closely related invertebrate orthologues (this study and [5]. Btrc/Fbxw1a is a substrate-recognition component of a ubiquitin ligase complex, which mediates the ubiquitination of proteins involved in cell cycle progression, signal transduction and transcription [22]. The protein contains one F-box and seven WD repeats. Genome searches and maximum likelihood analysis identified two *Btrc* orthologues in vertebrate genomes. *Btcr/Fbxw1a* genes are linked to the *Lbx1* loci and *Btrc2* genes, also called *Fbxw11*, are linked to the *Tlx3* loci. Our analysis placed the vertebrate proteins into two groups, in line with their genomic localisation.

Beta-transducin repeat containing (Btrc/Fbxw11)

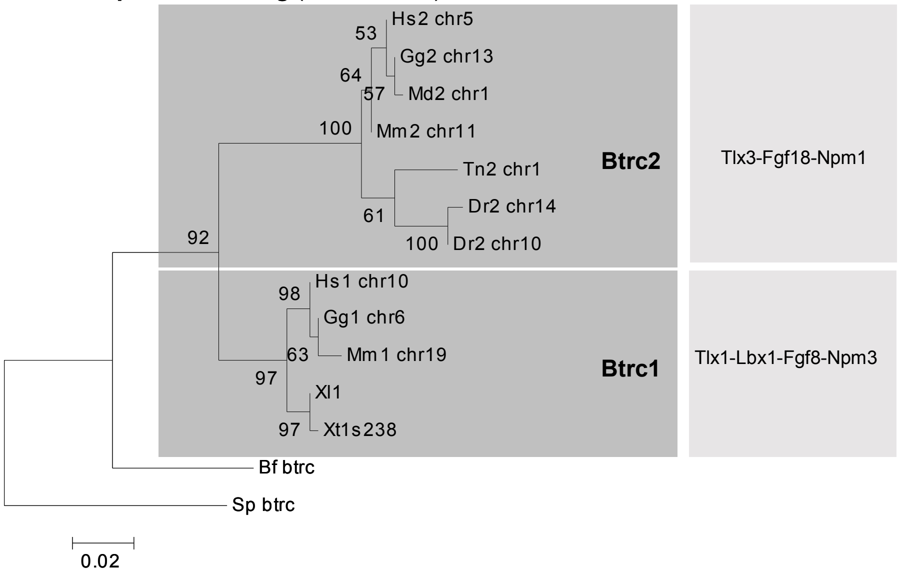

Mgea: Meningioma-expressed antigen 5

(*Homo sapiens* chr10: MGEA5, *Gallus gallus* chr4:MGEA2)

MGEA5 has been proposed as a candidate gene for type 2 diabetes mellitus in Mexican Americans [23]. The protein, which shows highest expression in the brain, placenta and pancreas, is a hyaluronidase, cleaving GlcNAc but not GalNAc from glycopeptides [24]. Genome searches and maximum likelihood analysis identified invertebrate *mgea* genes and two vertebrate orthologues, with *Mgea5* linked to the *Lbx1* loci, which includes teleost specific duplications, and *Mega2* (our name) linked to the putative *Lbx2* locus chicken. Phylogenetic analysis placed all Mgea5 sequences into one group, separate from Mgea2 sequences.

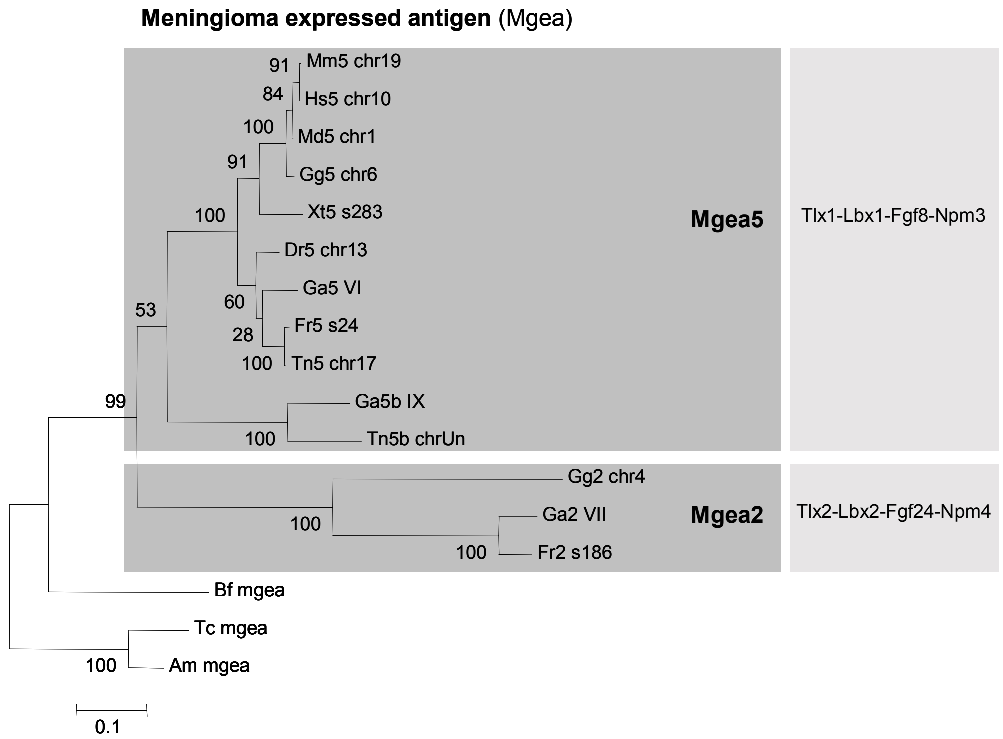

Kazal-like domain protein family

(*Homo sapiens* chr10: KAZALD1, *Danio rerio* chr14: KazalD2)

Kazald1, the kazal-type serine protease inhibitor domain-containing protein 1, is thought to be a secreted protein and has been implicated in the control of osteoblast proliferation during bone formation and bone regeneration [25]. Genome searches and maximum likelihood analysis identified an amphioxus kazald gene and three *Kazald* orthologues in vertebrate genomes, *Kazald1*, linked to the *Lbx1* loci, *Kazald2* (our name) linked to the *Lbx2* locus in the zebrafish genome and *Kazald3* on the same chromosome as *Fgf17* and *Npm3* in the zebrafish genome. Notably, the Kazald1 sequences grouped together, separate from Kazald2.

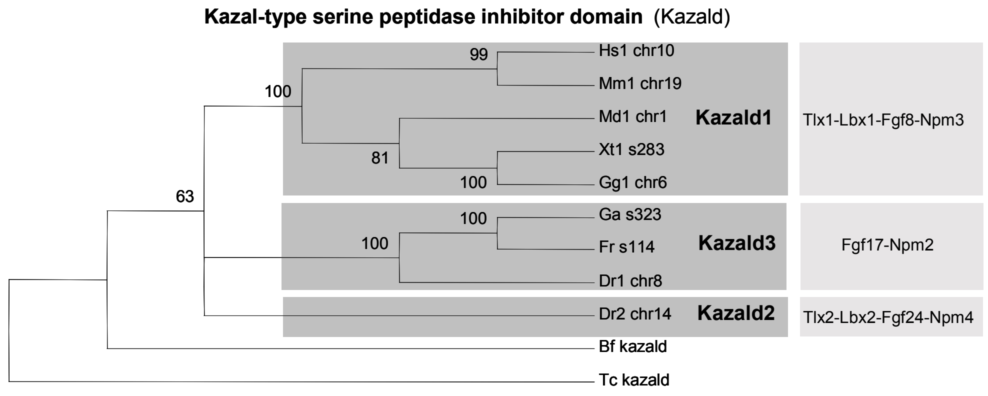

Ldb: Lim domain-binding protein

(*Homo sapiens* chr10: LDB1, chr4: LDB2)

Ldb1 and *Ldb2* genes contain the LIM binding domain and were discovered by their ability to bind LIM-homeodomain and LIM-only proteins and carry out a range of cellular functions via these protein interactions [26]. Genome searches and maximum likelihood analysis identified two *Ldb* orthologues in tetrapods, three in teleosts and a single representative in invertebrates. *Ldb1* sequences including the currently named *Ldb4* gene located on zebrafish chromosome 13 always coincided with *Lbx1* loci and constituted one group. *Ldb2* sequences constituted the second group. Notably, in teleosts, *Ldb2* genes are associated with *Lbx2* and in tetrapods they are associated with the now *Lbx*-less locus carrying *Nkx3.2*, consistent with our model that the tetrapod *Lbx2* locus once was associated with *Nkx3.2*.

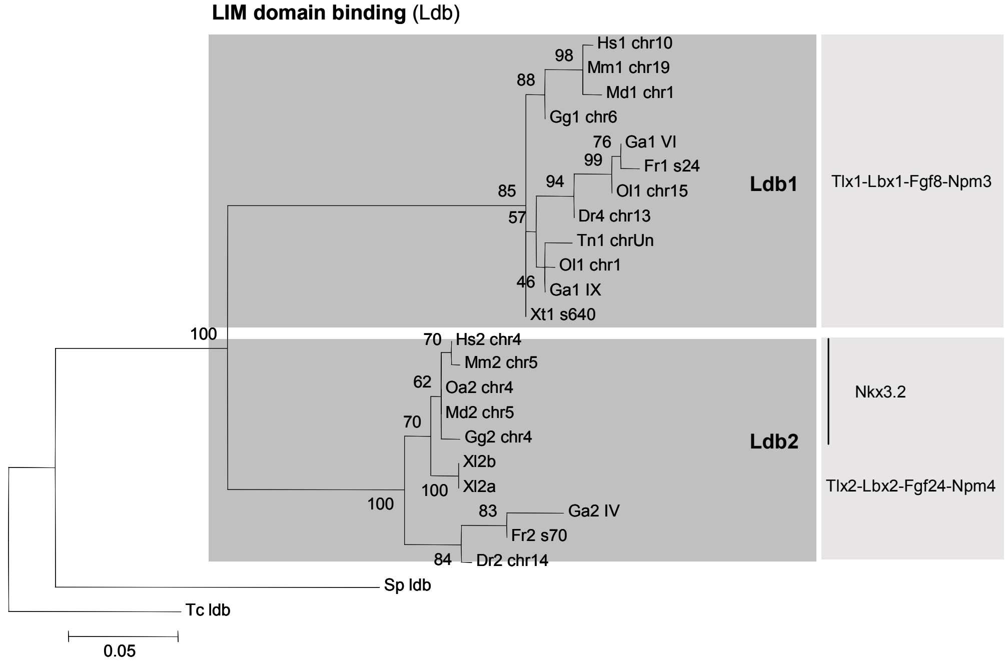

Prom: Prominin

(*Homo sapiens* chr4: PROM1, chr2: PROM2)

PROM1 and PROM2 are cholesterol binding multipass membrane proteins [27, 28]. Defects in PROM1 are the cause of an autosomal recessive form of retinal degeneration [29]. PROM2 appears to be restricted to epithelial cells [28]. Genome searches and maximum likelihood analysis identified two *Prom* orthologues in tetrapods, three in teleosts and one in invertebrates.

In tetrapods, *Prom1* was associated with *Fgfbp, Tapt1, Ldb2* and located on the now *Lbx*-less chromosomes carrying *Nkx3.2*. In the teleosts, one *Prom1-Fgfbp-Tapt1-Ldb2* set was associated with *Lbx2* loci. The second, more limited *Prom1-Fgfbp-Tapt1* set was on the chromosomes carrying the second of the *Lbx1* loci. However, it is inserted at varying sites and at a distance to *Lbx1*, suggesting that it originated from another locus, possibly the second, now dispersed *Lbx2* locus.

Tetrapod *Prom2* was associated with *Kcnip3* and in human, cattle and dog, it resides on the same chromosome as, but at a distance from, *Lbx2*. By contrast, in *Monodelphis*, chicken and *Xenopus*, *Prom2* was associated with *Loxl2, Nkx2.6 and Nkx3.1*, which in amniotes are linked to *Fgf17-Npm2* (*Xenopus* scaffolds were too short to determine linkage). The gene currently named *Prom2* in teleosts was associated with *Ldb1/4*, and in stickleback and Medaka, also with *Sufu* and *Kcnip2*. These genes were invariantly associated with *Lbx1* in all vertebrates investigated.

Phylogenetic analyses placed all *Prom1* genes into one group, separated from the *Prom2* genes, supporting the idea that they all once belonged to *Lbx2* carrying loci. This again supports our model that the tetrapod *Lbx2* loci was originally located in the *Nkx3.2* containing cluster. The *Prom2* genes however formed two clearly distinguished subgroups, with tetrapod sequences belonging to one, teleost sequences to the other subgroup. Given the distinct genomic environment of tetrapod and teleost *Prom2* genes, it is likely that teleost *Prom2* represents the *Prom* gene once linked to *Lbx1* and lost in tetrapods, while the tetrapod *Prom2* gene is a remnant of the fourth *Lbx* locus and was lost in teleosts. Hence we

rename the teleost *prom2* gene *prom3*. This data suggests a close relationship of *Lbx1* and former *Lbx4* loci, with different *Prom* orthologs being lost in lobe-finned and ray-finned fish lineages.

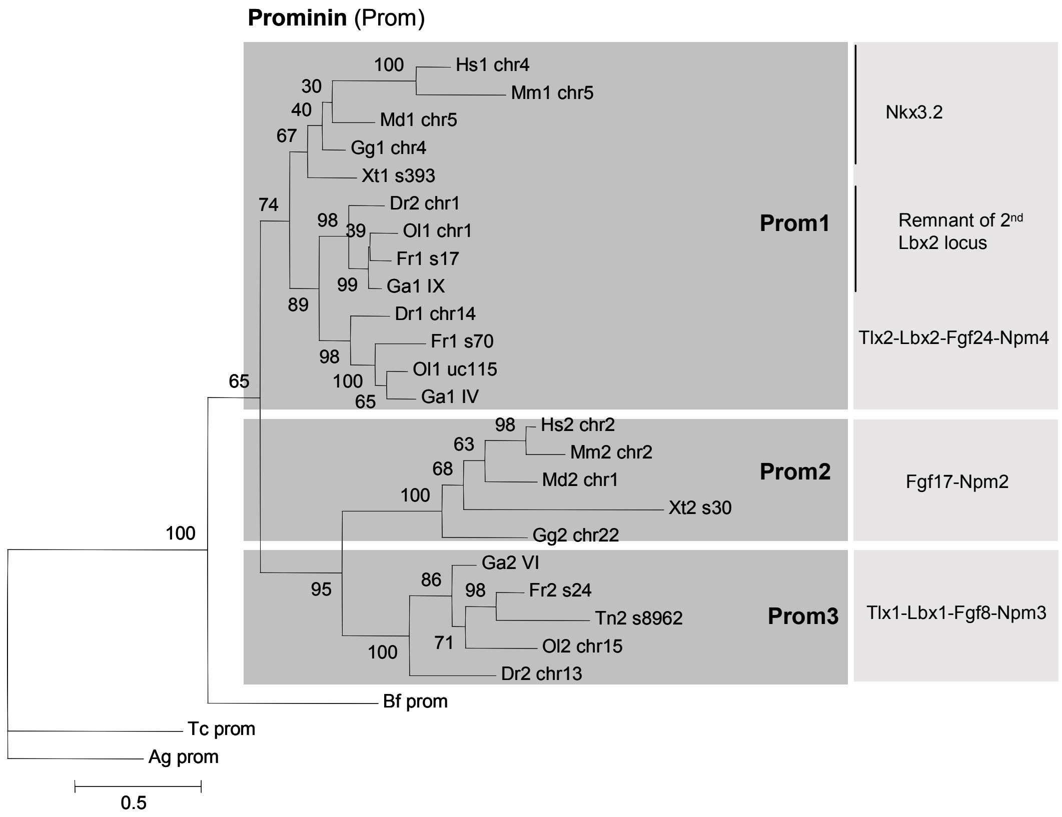

<u>Genes linked to three *Lbx* loci: *Tlx, Loxl, Slit, Dok, Add, Adra2*</u>

Tlx: T cell leukemia homeobox

(*Homo sapiens* chr10: TLX1, chr2: TLX2, chr5: TLX3)

Tlx proteins are Nk-type homeo domain containing transcription factors; the genes are thought to have arisen from tandem duplications of an ancestral NK gene early in animal evolution [10]. A chromosomal aberration involving *TLX1* is believed to cause of a form of T-cell acute lymphoblastic leukemia [30]. The function of *Tlx2* and *Tlx3* is less clear. However, it has been shown that *Tlx3*, together with *Tlx1*, are post-mitotic selector genes determining glutamatergic over GABAergic neuronal cell fates [31].

Phylogenetic analysis of the *Tlx* genes assigned the sequences to 3 distinct groups, with *Tlx* genes co-localising with *Lbx1* genes placed into the *Tlx1* group, *Tlx2* genes linked to *Lbx2* genes placed into the *Tlx2* group and *Tlx* genes not linked to any *Lbx* gene but instead to *Kcnip1* placed into the *Tlx3* group. The separation of the *Tlx1* group from the *Tlx2* and *3* groups is supported by high bootstrap values. The segregation of the *Tlx2* and *Tlx3* groups is less well supported, indicating that *Tlx2* and *3* sequences are more closely related.

Our analysis suggests that the previous assignment of zebrafish *Tlx2* (chr14) and *Tlx3* (chr10) as *Tlx3a* and *Tlx3b* [32], respectively, is incorrect.

T-cell leukaemia homeobox (*Tlx*)

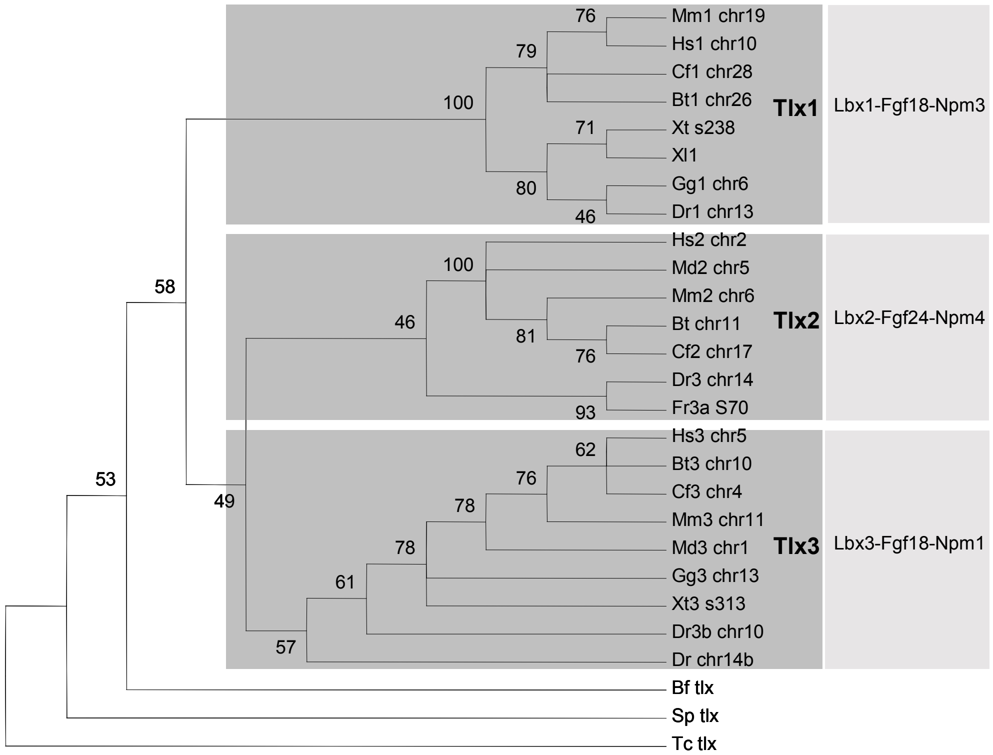

Loxl: Lysyl oxidase like

(*Homo sapiens* chr10: LOXL4, chr2: LOXL3, chr8: LOXL2)

Lysyl oxidase like proteins are copper-dependent amine oxidases. These secreted proteins are expressed in many tissues, highest expression being in reproductive tissues. Lysyl oxidases have been implicated in the cross-linking of extracellular matrix substrates, catalysing the covalent cross linking of fibrillar collagen types and the formation of cross-links in elastin. A lysine tyrosylquinone cross-link is generated by condensation of the ε-amino group of a lysine with a topaquinone produced by oxidation of tyrosine [33]. Genome searches and maximum likelihood analysis identified three *Loxl* orthologues in vertebrate genomes and a single *loxl* in *Ciona intestinalis*, to which a fourth vertebrate *Loxl* gene called *Loxl1* is more distantly related. Our analysis placed all the *Lbx1*-linked *Loxl4* genes into one group, the *Loxl2* genes associated with *Fgf17-Npm2* loci into the second, and the genes associated *Lbx2* loci into the third.

Lysyl oxidase-like (Loxl)

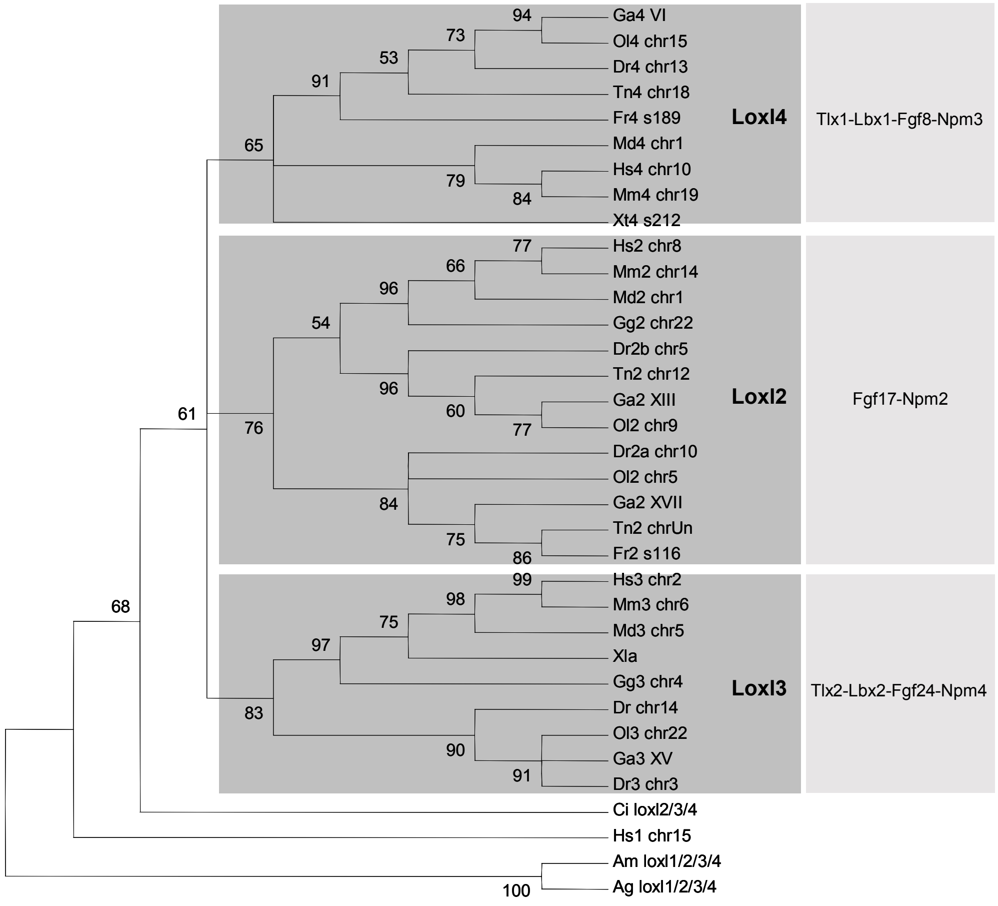

Slit

(*Homo sapiens* chr10: SLIT1, chr4: SLIT2, chr5: SLIT3)

SLIT1 is believed to act as a molecular guidance cue in cellular migration [34, 35]. SLIT1 and SLIT2 together seem to be essential for midline guidance in the forebrain. They act as repulsive signals preventing inappropriate midline crossing by axons projecting from the olfactory bulb [36].

Slit proteins are a family of large, secreted glycoproteins that act via their transmebrane receptor Robo to control axon guidance in both protostomes and deuterostomes. More recent evidence suggested roles of Slit molecules in the vasculature and immune system [37]. Genome searches and maximum likelihood analysis identified three *Slit* orthologues in tetrapods, four orthologues in teleosts, and one slit gene in invertebrates. Phylogenetic analyses placed the *Lbx1*-associated *Slit1* genes into one group. The second group consisted of the *Prom1*-associated *Slit2* genes, which in tetrapods are linked to the preserved *Lbx2* loci, and in teleosts to the remnant of the second *Lbx2* locus, now transferred to the chromosome carrying the second *Lbx1* locus. The third group consisted of *Slit3* genes associated with *Tlx3* loci.

Slit homolog (Slit)

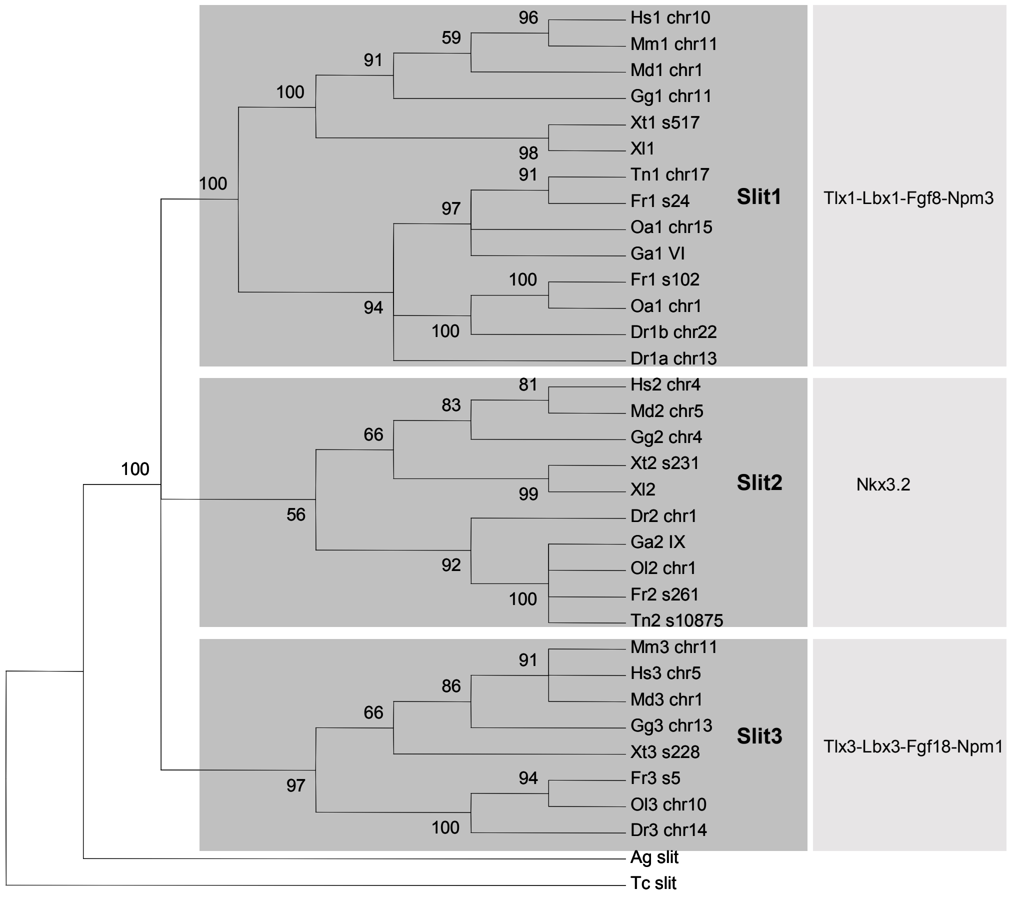

DOK: Docking downstream of tyrosine kinase

(*Homo sapiens* chr2: DOK1, chr8: DOK2, chr5: DOK3)

The DOK family of proteins interact with receptor tyrosine kinases and mediate particular biological responses [38]. DOK1 appears to be a negative regulator of the insulin signalling pathway [39]. DOK2 may modulate the cellular proliferation induced by IL-4, as well as IL-2 and IL-3 [40] and DOK3 is a negative regulator of JNK signalling in B-cells [41].

Three Dok genes were found linked to *Lbx* loci. Phylogenetic analyses placed the *Dok1* genes associated with the tetrapod *Lbx2* locus and the dispersed second teleost *Lbx2* locus into one group. The second group consisted of the *Tlx3*-associated *Dok3* genes. The *Dok2* genes associated with *Fgf17-Npm2* were rather divergent in sequence and did not group.

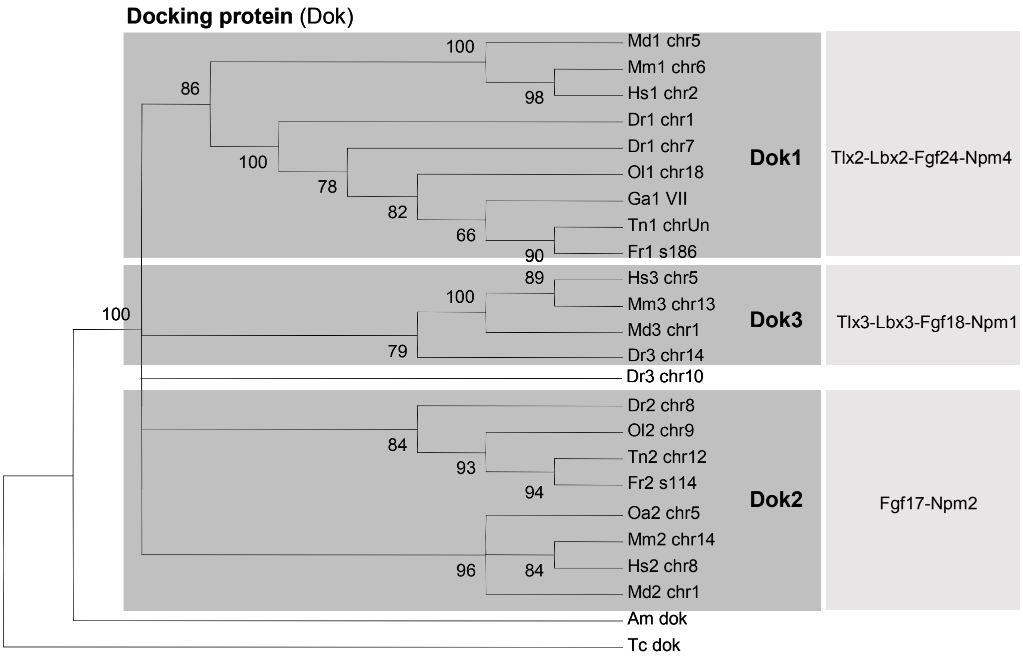

ADRA2: Alpha2A-adrenergic receptor

(*Homo sapiens* chr10: ADRA2A, chr2: ADRA2B. chr4: ADRA2C)

Alpha-2-adrenergic receptors are members of the G protein-coupled receptor superfamily involved in regulating neurotransmitter release from sympathetic nerves and from adrenergic neurons in the central nervous system. In humans, *ADRA* paralogs have been reported to reside on chromosome 2 (*LBX2* carrying) and chromosome 4 (*NKX3.2* carrying). This does not appear to support our model that the *Lbx2* locus was originally located in the *Nkx3.2* containing cluster and hence, needs to be investigated.

Phylogenetic analysis reveals 3 groups consisting of *Adra2a*, *Adra2b* and *Adra2c* sequences. In the genome, *Adra2a* genes associate with *Add3*, *Mxi* and *Smdc1* in all bony vertebrates investigated, and this gene group was invariantly found on *Lbx1* carrying chromosomes with the exception of *Danio rerio* (chromosome 22) and the frog (not present in the current assembly).

Adra2b genes associate with *Kncip3*, *Prom2* and *Dusp2* (individual genes lost from the set in some species), which in placental mammals are located on the *Lbx2*-carrying chromosome, but at a distance to this gene. In all other bony vertebrates studied, this gene set is linked with remnants of the *Lbx4/Tlx4* loci.

Tetrapod *Adra2c* genes reside in a gene group encompassing *Add1*, *Dok7*, *Lrpap* and *Hmx1*; this group is split in teleosts. In mammals, the genes are associated with the now *Lbx*-less, *Nkx3.2* carrying chromosome; the chicken and teleost sequences, however, are associated with chromosomes that carry both *Nkx3*.2 and *Lbx2*.

Taken together, phylogeny and genomic localisation of *Adra* genes suggests that *Adra2a* is associated with the *Lbx1* paralogon and *Adra2c* was ancestrally associated with the *Lbx2* paralogon. *Adra2b* was associated with the fourth *Lbx* paralogon with the *Adra2b* genes of placental mammals translocating onto the *Lbx2*-carrying chromosome; the same pattern of movement is seen for the *Prom* and *Kcnip* genes.

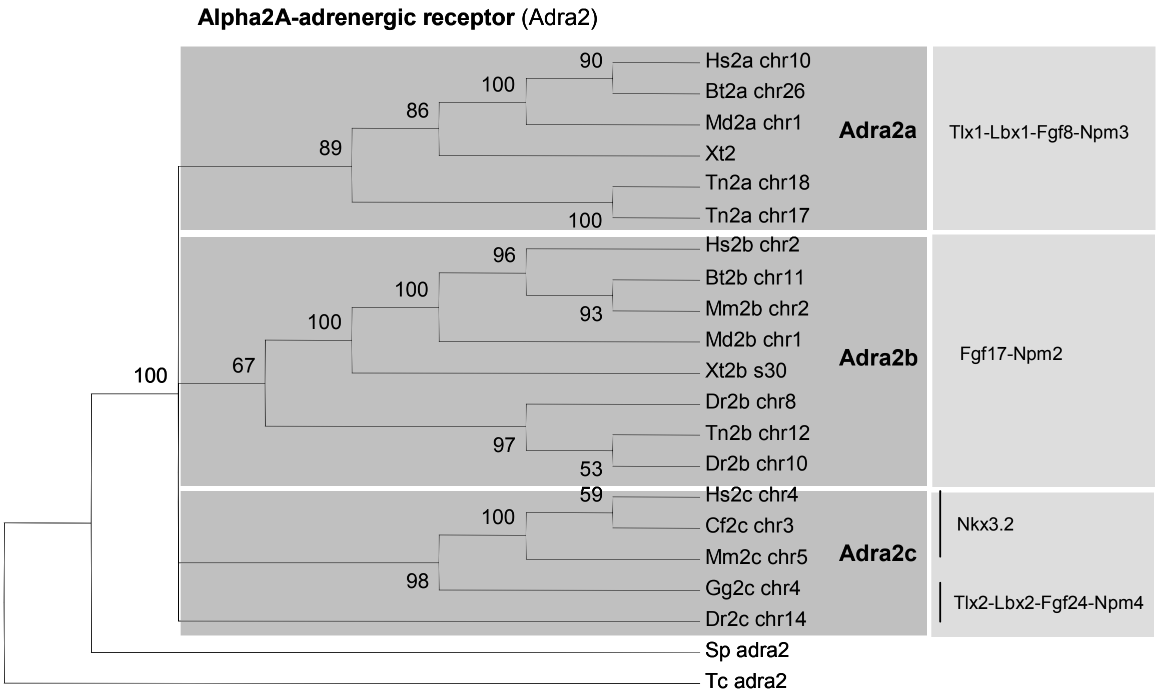

ADD: Adducin

(*Homo sapiens* chr4: ADD1, chr2: ADD2, chr10: ADD3.)

Adducins are a family of cytoskeleton proteins. Similar to *Adra* genes, paralogous *Add* genes were found on human chromosome 4 (*NKX3.2* carrying) and chromosome 2 (*LBX2* carrying). This does not appear to support our model that *Lbx2* loci were originally linked to *Nkx3.2* and hence, needs to be investigated.

Phylogenetic analysis reveals 3 groups of Add genes, *Add1*, *Add2* and *Add3*. *Add3* genes were found within the same gene set as *Adra2a* and, with the exception of zebrafish (chromosome 22) and the frog (not present in the current assembly) *Add3* genes are associated with *Lbx1* loci.

Add2 genes were found grouped with *C2orf42, Pcyox1, Fam136a, Tgfa* or *Mxd1* in tetrapods; the link to *Mxd1* was maintained in teleosts. In the marsupial *Monodelphis domestica*, the chicken and in teleosts, *Add2* genes reside on chromosomes carrying remnants of the fourth *Lbx* locus (dispersed 4th

paralogon in teleosts). In placental mammals, the localisation of *Add2* varies between chromosomes so far not associated with *Lbx* genes (dog chr 10) and sites distant (cattle chr 11) or quite close to (human chr2, mouse chr 6) *Lbx2*, suggesting that the gene set encompassing *Add2* has transposed to new sites a number of times.

Add1 genes were found associated with *Nol14* in tetrapods and in medaka and stickleback; in tetrapods the genes are embedded in a larger gene group that also encompassed *Adra2c*. Where the genomic information was sufficient to determine the chromosomal localisation, we found that tetrapod *Add1* is located on the chromosome today only carrying *Nkx3.2* (placental mammals), or carrying parts of the *Nkx3.2* site as well as the site for the *Lbx2* gene (opossum, chicken). In teleosts, the localisation of the gene is less conserved and includes zebrafish chromosome 21, so far not associated with *Lbx* loci.

Taken together, cumulative evidence suggests that *Add1* belonged to the cluster that originally contained *Nkx3.2* and *Lbx2*, while *Add2* belonged to the fourth Lbx paralogon. After *Nkx3.2* and *Lbx2* separated, *Add2* translocated to - distinct - sites on the *Lbx2* carrying chromosome in the lineage leading to humans, mouse and cattle. Notably, Add3 and Add2 sequences form a subgroup in the phylogenetic tree, the separation from Add1 is supported by high bootstrap values. A similar subgrouping of Lbx1-Lbx4 (or Lbx2-Lbx3) associated sequences has also been observed for Fgf, Npm and Tlx sequences.

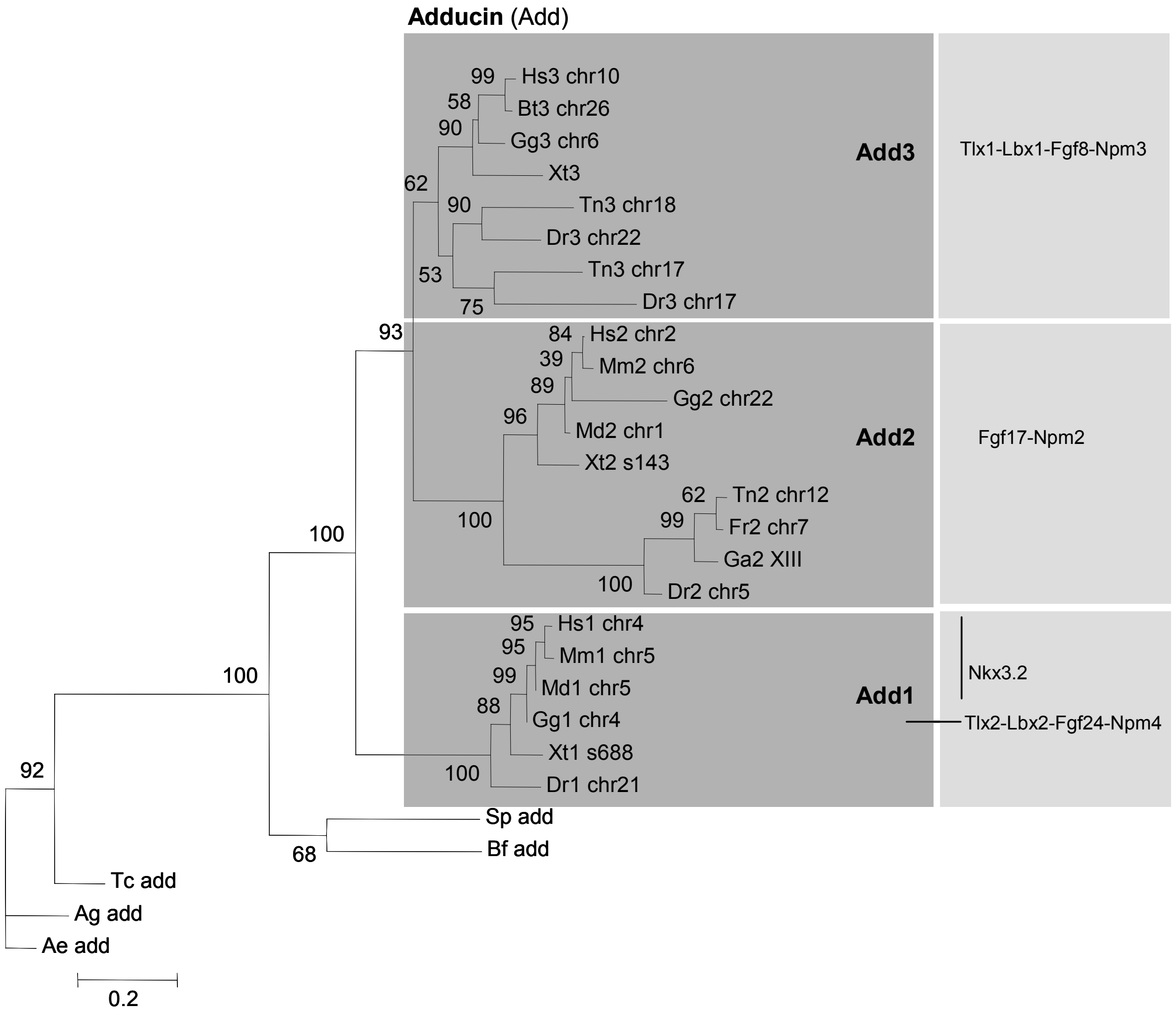
Adducin (Add)
Add3
Tlx1-Lbx1-Fgf8-Npm3
Hs3 chr10
Bt3 chr26
Gg3 chr6
Xt3
Tn3 chr18
Dr3 chr22
Tn3 chr17
Dr3 chr17
Add2
Fgf17-Npm2
Hs2 chr2
Mm2 chr6
Gg2 chr22
Md2 chr1
Xt2 s143
Tn2 chr12
Fr2 chr7
Ga2 XIII
Dr2 chr5
Add1
Nkx3.2
Tlx2-Lbx2-Fgf24-Npm4
Hs1 chr4
Mm1 chr5
Md1 chr5
Gg1 chr4
Xt1 s688
Dr1 chr21
Sp add
Bf add
Tc add
Ag add
Ae add
0.2

<u>Genes linked to four *Lbx* loci: *Fgf, Npm, Kcnip*</u>

Fgf: Fibroblast growth factor

(*Homo sapiens* chr10: FGF8, chr8: FGF17, chr5: FGF18)

Member of this family of signalling molecules are secreted and play multiple roles in the embryo and the adult, regulating cell proliferation, cell specification, axon guidance and developmental patterning [42]. Fgf8 is most famous for its role in limb and branchial arch development and the isthmic organiser [43]. It also stimulates cell growth in an autocrine manner and it has been shown to mediate hormonal action on the growth of cancer cells [44]. Fgf17 may be a signalling molecule in the induction and patterning of the embryonic brain, and Fgf18 has been shown to stimulate hepatic and intestinal proliferation [45, 46].

Genome searches and maximum likelihood analysis identified four vertebrate *Fgf* orthologues of the single *Ciona intestinalis fgf 8/17/24/18* gene. Notably, *Lbx1*-linked *Fgf8* is closely related to *Fgf17* with strong bootstrap support, while *Tlx3*-linked *Fgf18* is more closely related to *Lbx2*-linked *Fgf24*, although the support for this relationship is lower.

Fibroblast growth factors 8/17/24/18 (Fgf)

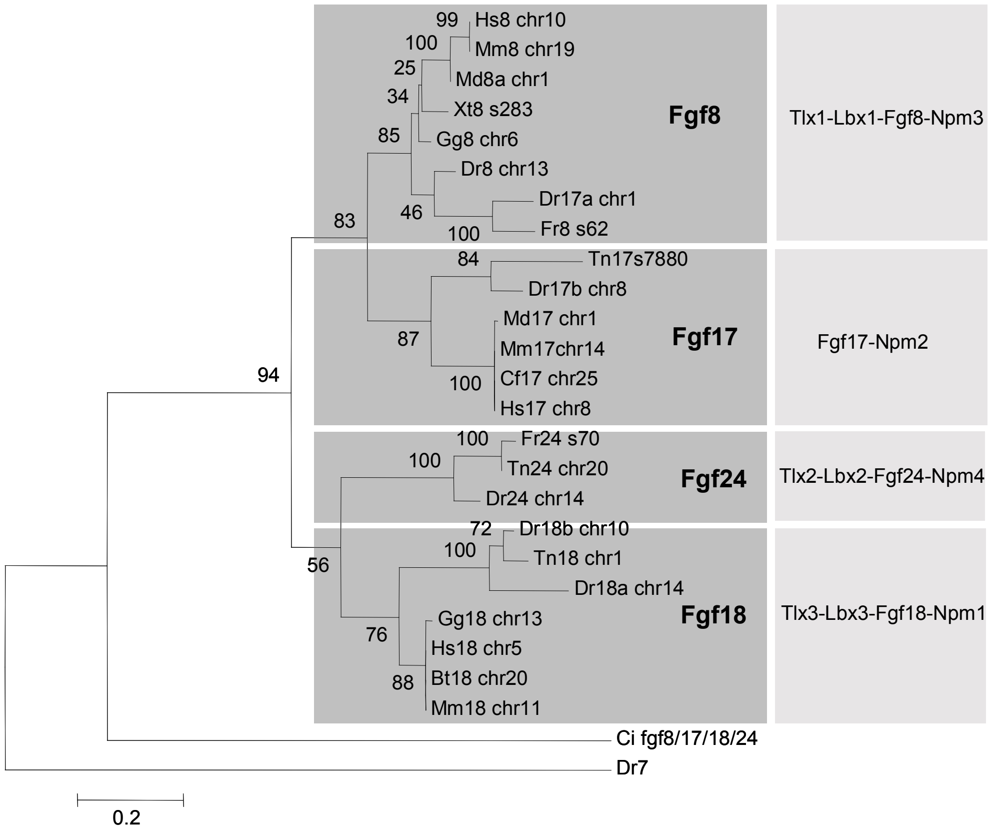

Npm: Nucleoplasmin/Nucleophosmin

(*Homo sapiens* chr10: NPM3, chr8: NPM2, chr5: NPM1)

Npm1 is a protein associated with nucleolar ribonucleoprotein structures which binds single-stranded nucleic acids. It may function in the assembly and/or transport of ribosomes. Human NPM1 has been implicated in various diseases, particularly in forms of leukemia [47]. Npm2 is thought to be involved in sperm DNA decondensation during fertilization. It is found in the oocyte nucleus before nuclear membrane breakdown, after which it is redistributed to the cytoplasm [48]. Npm3 has been less characterised, although available evidence suggests that it may act as a chaperone [49]. Our genome searches and maximum likelihood analyses identified a fourth *Npm* ortholog (*Npm4*) in vertebrate genomes.

Phylogenetic analyses placed *Npm* genes into four groups, with the *Lbx1*-associated *Npm3* genes forming the first, the *Fgf17*-associated *Npm2* genes the second, the *Npm4* genes associated with teleost *Lbx2-Fgf24* the third, and *Tlx3*-associated *Npm1* genes the fourth group. Notably, Npm3 and Npm2 sequences formed a subgroup, distinguished from Npm4 and Npm1 sequences forming another subgroup. The grouping of Npm4 and 1 is supported by high bootstrap values. This suggests that during the second vertebrate genome duplication, *Lbx2/Tlx2* and *Tlx3* loci arose from one ancestral locus, and by inference*, Lbx1/Tlx1* and the former *Lbx4/Tlx4* loci from the second ancestral locus.

Interestingly, we identified a Npm4 sequence from the chondrichthian *Leucoraja erinacea* (little skate) that groups with the teleost Npm4 sequences, suggesting that the duplications producing this gene, and by inference the *Lbx* loci, were complete before the divergence of chondrichthyan and osteichthyan lineages.

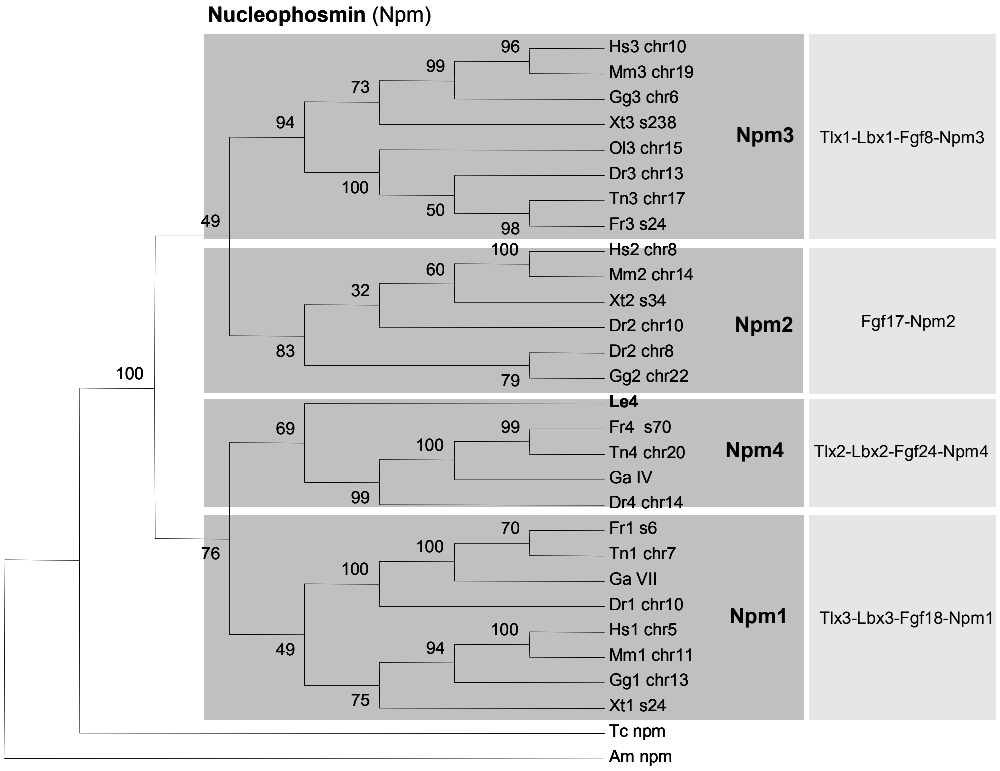

Kcnip: Kv channel-interacting protein

(*Homo sapiens* chr10: KCNIP2, chr2: KCNIP3, chr5: KCNIP1, chr4: KCNIP4)

Proteins of this family are characterised by four EF-hand domains. The are predominantly expressed in the brain where they form the regulatory subunits of the Kv4 family of K (+) channels, probably modulating channel density, inactivation kinetics and rate of recovery from inactivation in a calcium-dependent and isoform-specific manner [50]. Kcnip proteins have also been implicated in the trafficking of Kcnd2 and Kcnd3 proteins that generate the ion channels Kv4.2 and Kv4.3, respectively, to the cell surface [51].

Genome searches and maximum likelihood analysis identified four *Kcnip* orthologues in vertebrate genomes and one invertebrate *kcnip*. Phylogenetic analyses placed the *Lbx1*-associated *Kcnip2* genes into one group, the *Fgf17-Npm2-Loxl2* associated *Kcnip3* genes (note: linkage broken in humans, dog and cattle with *Kcnip3-Prom2* being translocated to the *Lbx2* carrying chromosome) into the second group, the *Tlx3-Npm1* associated *Kcnip1* genes into the third group, and the *Kcnip4* genes linked to

tetrapod *Lbx2* and the remnant of the second teleost *Lbx2* locus into the fourth. *Kcnip1* and *Kcnip4* genes formed a subgroup supported by high bootstrap values, reinforcing the notion that *Lbx2/Tlx2* and *Tlx3* loci are closely related and possibly arose from a common ancestor.

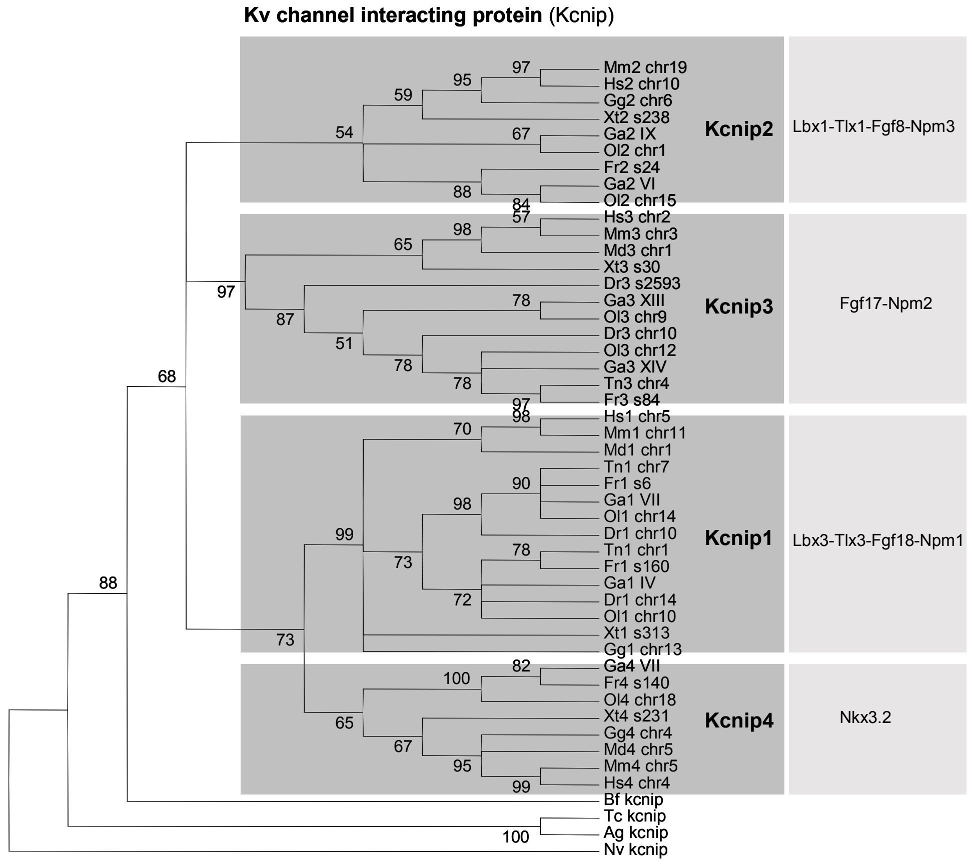

Sequence identification numbers and nomenclature

The following are tables giving accession numbers or other identifiers for the sequences used in these analyses. Where current nomenclature is available, a name is given in the appropriate column. Where absent, a proposed name has been given. Moreover, where the current nomenclature is misleading, a different proposed name has been suggested.

ADD				
**Abbreviation**	**Species**	**Current Nomenclature**	**Proposed Nomenclature**	**ID**
Ae add	Aedes aegypti	add		XP_001661417.1
Ag add	Anopheles gambiae		add	XP_001688561.1
Bf add	Branchiostoma floridae		add	Brafl1:126139
Bt3 chr26	Bos taurus	Add3		NP_001068662.1
Dr1 chr21	Danio rerio	Add1		NP_001073427.1
Dr2 chr5	Danio rerio		Add2	XP_698680.2
Dr3 chr17	Danio rerio		Add3	XP_683269.1
Dr3 chr22	Danio rerio	Add3		NP_955957.1
Fr2 chr7	Takifugu rubripes	Add2		ENSTRUT00000037799
Ga2 XIII	Gasterosteus aculeatus	Add2		ENSGACT00000017476
Gg1 chr4	Gallus gallus		Add1	CAH65186.1
Gg2 chr22	Gallus gallus		Add2	hmm31954
Gg3 chr6	Gallus gallus	Add3		NP_989434.1
Hs1 chr4	Homo sapiens	ADD1		NP_789771.1
Hs2 chr2	Homo sapiens	ADD2		AAH65525.1
Hs3 chr10	Homo sapiens	ADD3		BAD96990.1
Md1 chr5	Monodelphis domestica	Add1		XP_001365080.1
Md2 chr1	Monodelphis domestica	Add2		XP_001381767.1
Mm1 chr5	Mus musculus	Add1		NP_001095914.1
Mm2 chr6	Mus musculus		ADD2	BAC25943.1
Sp add	Strongylocentrotus purpuratus	add2	add	XP_780526.2
Tc add	Tribolium castaneum		add	XP_970946.1
Tn2 chr12	Tetraodon nigroviridis		Add2	CAF97138.1
Tn3 chr17	Tetraodon nigroviridis		Add3	GSTENP00024942001
Tn3 chr18	Tetraodon nigroviridis		Add3	GSTENP00020027001
Xt1 s688	Xenopus tropicalis	Add2	Add1	NP_001005674.1
Xt2 s143	Xenopus tropicalis		Add2	NP_001096433.1
Xt3	Xenopus tropicalis	Add3		AAH76963.1

ADRA2				
Abbreviation	Species	Current Nomenclature	Proposed Nomenclature	ID
Bt2a chr26	Bos taurus	Adra2a		NP_776924.1
Bt2b chr11	Bos taurus	Adra2b		XP_594575.2
Cf2c chr3	Canis familiaris	Adra2c		XP_545911.2
Dr2b chr10	Danio rerio	Adra2c	Adra2b	XP_001338337.1
Dr2b chr8	Danio rerio	Adra2b		NP_997521.1
Dr2c chr14	Danio rerio	Adra2d	Adra2c	NP_919345.2
Gg2c chr4	Gallus gallus	Adra2c		XP_426355.2
Hs2a chr10	Homo sapiens	ADRA2a		NP_000672.2
Hs2b chr2	Homo sapiens	ADRA2b		NP_000673.2
Hs2c chr4	Homo sapiens	ADRA2c		NP_000674.2
Md2a chr1	Monodelphis domestica	Adra2a		XP_001378051.1
Md2b chr1	Monodelphis domestica	Adra2b		XP_001382061.1
Mm2b chr2	Mus musculus	Adra2b		AAK56078.1
Mm2c chr5	Mus musculus	Adra2c		NP_031444.2
Sp adra2	Strongylocentrotus purpuratus		adra22	XP_001200819.1
Tc adra2	Tribolium castaneum		adra22	XP_970290.1
Tn2a chr17	Tetraodon nigroviridis		Adra2a	GIDT00016403001
Tn2a chr18	Tetraodon nigroviridis		Adra2a	GSTENP00020015001
Tn2b chr12	Tetraodon nigroviridis		Adra2b	GSTENP00028594001
Xt2	Xenopus tropicalis	Adra2a		NP_001072843.1
Xt2b s30	Xenopus tropicalis		Adra2b	Xentr4:449615

Aup1				
Abbreviation	Species	Current Nomenclature	Proposed Nomenclature	ID
Am aup1	Apis mellifera	aup1		XP_392941.2
Bf aup1	Branchiostoma floridae		aup1	estExt_gwp.C_2940024
Dr1 chr14	Danio rerio	Aup1		NP_955984.1
Gg1 chr4	Gallus gallus	Aup1		XP_001232437.1
Hs1 chr2	Homo sapiens	AUP1		NP_853553.1
Md1 chr5	Monodelphis domestica	Aup1		XP_001376691.1
Mm1 chr6	Mus musculus	Aup1		NP_031543.2
Nv aup1	Nematostella vectensis		aup1	XP_001624449.1
Slime mould aup1	Dictyostelium discoideum		aup1	XP_638792.1
Sp aup1	Strongylocentrotus purpuratus	aup1		XP_001189935.1

Aup1				
Abbreviation	Species	Current Nomenclature	Proposed Nomenclature	ID
Tc aup1	Tribolium castaneum	aup1		XP_966748.1
Xl1	Xenopus laevis		Aup1	AAI29706.1

Btrc				
Abbreviation	Species	Current Nomenclature	Proposed Nomenclature	ID
Bf fbxw11	Branchiostoma floridae		btrc	fgenesh2_pg.scaffold_690000004
Dr2 chr10	Danio rerio	Fbxw11	Btrc2	NP_958467.1
Dr2 chr14	Danio rerio	Fbxw11	Brtc2	NP_958467.1
Gg1 chr6	Gallus gallus	Btrc	Btrc1	XP_421723.2
Gg2 chr13	Gallus gallus	Fbxw11	Btrc2	NP_001034351.1
Hs1 chr10	Homo sapiens	BTRC	BTRC1	NP_378663.1
Hs2 chr5	Homo sapiens	FBXW11	BTRC2	NP_036432.2
Md11	Monodelphis domestica	Fbxw11	Btrc2	XP_001380485.1
Mm1 chr19	Mus musculus	Btrc	Btrc1	NP_001032847.1
Mm2 chr11	Mus musculus	Fbxw11	Btrc2	NP_598776.1
Sp btrc	Strongylocentrotus purpuratus		btrc	XP_784183.2
Tn2 chr1	Tetraodon nigroviridis		Btrc2	GSTENP00005198001
Xl1	Xenopus laevis	Btrc	Btrc1	NP_001081064.1
Xt1 s238	Xenopus tropicalis	Btrc	Btrc1	NP_001016386.1

Dok				
Abbreviation	Species	Current Nomenclature	Proposed Nomenclature	ID
Am dok	Apis mellifera	dok		XP_394099.3
Dr1 chr1	Danio rerio		Dok1	XP_687576.2
Dr1 chr7	Danio rerio	Dok1		NP_998253
Dr2 chr8	Danio rerio	Dok2		XP_001341514
Dr3 chr10	Danio rerio	Dok3		hmm26829
Dr3 chr14	Danio rerio	Dok3		XP_001339798.1
Fr1 s186	Fugu rubripes	Dok1		SINFRUG00000123411
Fr2 s114	Fugu rubripes	Dok2		SINFRUG00000126402
Ga1 VII	Gasterosteus aculeatus	Dok1		ENSGACG00000019876
Hs1 chr2	Homo sapiens	DOK1		NP_001372
Hs2 chr8	Homo sapiens	DOK2		NP_003965
Hs3 chr5	Homo sapiens	DOK3		NP_079148

Dok				
**Abbreviation**	**Species**	**Current Nomenclature**	**Proposed Nomenclature**	**ID**
Md1 chr5	Monodelphis domestica	Dok1		ENSMODG00000004738
Md2 chr1	Monodelphis domestica	Dok2		XP_001381967
Md3 chr1	Monodelphis domestica	Dok3		XP_001381063
Mm1 chr6	Mus musculus	Dok1		NP_034200
Mm2 chr14	Mus musculus	Dok2		NP_034201
Mm3 chr13	Mus musculus	Dok3		NP_038767
Oa2 chr5	Ornithorhynchus anatinus	Dok2		ENSOANG00000013185
Ol1 chr18	Oryzias latipes	Dok1		ENSORLG00000005897
Ol2 chr9	Oryzias latipes	Dok2		UTOLAPRE05100114776
Tc dok	Tribolium castaneum	dok		XP_971563.1
Tn1 chrUn	Tetraodon nigroviridis	Dok1		CAF89502
Tn2 chr12	Tetraodon nigroviridis	Dok2		CAF94982

Dpcd				
**Abbreviation**	**Species**	**Current Nomenclature**	**Proposed Nomenclature**	**ID**
Ag dpcd	Anopheles gambiae		dpcd	XP_307420.3
Bf dpcd	Branchiosto ma floridae		dpcd	estExt_fgenesh2_pm.C_1420 005
Dr chr11	Danio rerio		Dpcd	NP_001038842.1
Fr s62	Fugu rubripes		Dpcd	SINFRUP00000173098
Gg chr6	Gallus gallus	Dpcd		XP_421721.2
Hs chr10	Homo sapiens	DPCD		NP_056263.1
Md chr1	Monodelphi s domestica	Dpcd		XP_001369785.1
Mm chr10	Mus musculus	Dpcd		NP_766227.1
Tc dpcd	Tribolium castaneum		dpcd	XP_973930.1
Tn chr18	Tetraodon nigroviridis		Dpcd	CAG03352.1
Xt s283	Xenopus tropicalis	Dpcd		NP_001096275.1

Fbxw4

Abbreviation	Species	Current Nomenclature	Proposed Nomenclature	ID
Ag fbxw4	Anopheles gambiae		fbxw4	EAA11813.4
Dr4 chr13	Danio rerio	Fbxw4		NP_571596.1
Fr4 s52	Fugu rubripes		Fbxw4	SINFRUP00000165496
Gg4 chr6	Gallus gallus	Fbxw4		XP_001233793.1
Hs4 chr10	Homo sapiens	FBXW4		EAW49753.1
Hs9chr19	Homo sapiens	FBXW9		NP_115677.2
Mm4 chr19	Mus musculus	Fbxw4		NP_038935.1
Sp fbxw4	Strongylocentrotus purpuratus		fbxw4	XP_001196990.1
Xt4 s283	Xenopus tropicalis		Fbxw4	ENSXETG00000021190.2

Fbxw7				
Abbreviation	Species	Current Nomenclature	Proposed Nomenclature	ID
Ag fbxw7	Anopheles gambiae	fbxw7		XP_315369.4
Bf fbxw7	Branchiostoma floridae	fbxw7		107832
Dr7 chr1	Danio rerio	Fbxw7		XP_001341767
Fr7 s17	Fugu rubripes	Fbxw7		SINFRUP00000129810
Gg7 chr4	Gallus gallus	Fbxw7		XP_420447
Hs7 chr4	Homo sapiens	FBXW7		NP_361014
Md7 chr5	Monodelphis domestica	Fbxw7		ENSMODP00000001260
Mm1 chr11	Mus musculus	Fbxw1		CAI25520.1
Mm7 chr3	Mus musculus	Fbxw7		EDL15400
Oa7 uc539	Ornithorhynchus anatinus	Fbxw7		XP_001514172
Ol7 chr1	Oryzias latipes	Fbxw7		ENSORLP00000009720
Tn7 chr18	Tetraodon nigroviridis	Fbxw7		GSTENP00034549001

Fgf				
Abbreviation	Species	Current Nomenclature	Proposed Nomenclature	ID
Bt18	Bos taurus	Fgf18		NP_032031.1
Cf17	Canis familiaris	Fgf17		XP_849590.1
Ci fgf	Ciona intestinalis	fgf8/17/18	fgf8/17/24/18	NP_001027648.1
Dr17a chr1	Danio rerio	Fgf17a	Fgf8a	NP_878276.1
Dr17b chr8	Danio rerio	Fgf17		NP_999973.1
Dr18 chr10	Danio rerio	Fgf18		NP_001013282.1
Dr18a chr14	Danio rerio	Fgf18		NP_001012379.1
Dr24 chr14	Danio rerio	Fgf24		AAO38854.1
Dr7	Danio rerio	Fgf7		NP_001007762.1

Fgf				
**Abbreviation**	**Species**	**Current Nomenclature**	**Proposed Nomenclature**	**ID**
Dr8 chr13	Danio rerio	Fgf8	Fgf8b	AAC60303.1
Fr24 s70	Fugu rubripes	Fgf24		SINFRUP00000148211
Fr8 s62	Fugu rubripes	Fgf8		SINFRUP00000132521
Gg18 chr13	Gallus gallus	Fgf18		NP_990045.1
Gg8 chr6	Gallus gallus	Fgf8		CAG28796.1
Hs17 chr8	Homo sapiens	FGF17		NP_003858.1
Hs18 chr13	Homo sapiens	FGF18		NP_003853.1
Hs8 chr10	Homo sapiens	FGF8		NP_149353.1
Md17 chr1	Monodelphis domestica	Fgf17		XP_001373341.1
Md8 chr1	Monodelphis domestica	Fgf8		XP_001369748.1
Mm17 chr14	Mus musculus	Fgf17		NP_032030.1
Mm18 chr11	Mus musculus	Fgf18		NP_032031.1
Mm8 chr19	Mus musculus	Fgf8		AAH48734.1
Tn17 s7880	Tetraodon nigroviridis	Fgf17		CAF90913.1
Tn18 chr1	Tetraodon nigroviridis	Fgf18		CAF90784.1
Tn24 chr20	Tetraodon nigroviridis	Fgf17		CAG04671.1
Xt8 s283	Xenopus tropicalis	Fgf8		NP_001008163.1

Kazald				
**Abbreviation**	**Species**	**Current Nomenclature**	**Proposed Nomenclature**	**ID**
Bf kazald	Branchiostoma floridae		kazald	e_gw.1030.13.1
Dr chr14	Danio rerio		Kazald2	NP_001098594.1
Dr1 chr8	Danio rerio	Kazald1		XP_001336653.1
Frs114	Fugu rubripes		Kazald3	SINFRUP00000143064
Gg1 chr6	Gallus gallus		Kazald1	XP_421724.1
Ga s323	Gasterosteus aculeatus		Kazald3	ENSGACP00000001225
Hs1 chr10	Homo sapiens	KAZALD1		NP_112191.2
Md1 chr1	Monodelphis domestica	Kazald1		XP_001379416.1
Mm1 chr19	Mus musculus	Kazald1		NP_849260.1
Tc kazald	Tribolium castaneum		kazald	XP_968570.1
Xt1 s283	Xenopus tropicalis	Kazald1		NP_001093733.1

Kcnip

Abbreviation	Species	Current Nomenclature	Proposed Nomenclature	ID
Bf kcnip	Branchiostoma floridae	kcnip		e_gw.58.15.1
Dr1 chr10	Danio rerio		Kcnip1	NP_001008632.1
Dr1 chr14	Danio rerio	Kcnip1		ENSDARP00000046953
Dr3 chr10	Danio rerio	Kcnip3		AAI52213.1
Fr1 s160	Fugu rubripes		Kcnip1	SINFRUP00000131599
Fr1 s6	Fugu rubripes	Kcnip1		SINFRUP00000174294
Fr2 s24	Fugu rubripes	Kcnip2		SINFRUP00000146483
Fr3 s84	Fugu rubripes	Kcnip3		SINFRUP00000165597
Fr4 s140	Fugu rubripes	Kcnip4		SINFRUP00000127186
Ga1 IV	Gasterosteus aculeatus	Kcnip1		ENSGACP00000023624
Ga2 IX	Gasterosteus aculeatus	Kcnip2		ENSGACP00000024236
Ga2 VI	Gasterosteus aculeatus	Kcnip2		ENSGACP00000009775
Ga3 XIII	Gasterosteus aculeatus	Kcnip3		ENSGACP00000007549
Ga3 XIV	Gasterosteus aculeatus	Kcnip3		ENSGACP00000022594
Ga4 VII	Gasterosteus aculeatus	Kcnip4		ENSGACP00000025829
Gg1 chr13	Gallus gallus	Kcnip1		ENSGALP00000003327
Gg2 chr6	Gallus gallus	Kcnip2		NP_989581.1
Gg4 chr4	Gallus gallus	Kcnip4		NP_989886.1
Hs1 chr5	Homo sapiens	KCNIP1		NP_001030009.1
Hs2 chr10	Homo sapiens	KCNIP2		NP_055406.2
Hs3 chr2	Homo sapiens	KCNIP3		NP_038462.1
Hs4 chr4	Homo sapiens	KCNIP4		NP_671712.1
Md1 chr1	Monodelphis domestica	Kcnip1		XP_001370376.1
Md4 chr5	Monodelphis domestica	Kcnip4		XP_001369532.1
Mm1 chr11	Mus musculus	Kcnip1		Q9JJ57
Mm2 chr19	Mus musculus	Kcnip2		NP_663749.1
Mm3 chr3	Mus musculus	Kcnip3		CAM13446.1
Mm4 chr5	Mus musculus	Kcnip4		EDL37640.1
Nv kcnip	Nematostella vectensis	kcnip		XP_001634810.1
Ol1 chr10	Oryzias latipes	Kcnip1		ENSORLP00000004909
Ol1 chr14	Oryzias latipes	Kcnip1		ENSORLP00000012728
Ol2 chr1	Oryzias latipes	Kcnip2		ENSORLP00000015639
Ol2 chr15	Oryzias latipes	Kcnip2		ENSORLP00000010439
Ol3 chr12	Oryzias latipes	Kcnip3		ENSORLP00000011320
Ol3 chr9	Oryzias latipes	Kcnip3		ENSORLP00000003656

Kcnip				
**Abbreviation**	**Species**	**Current Nomenclature**	**Proposed Nomenclature**	**ID**
Ol4 chr18	Oryzias latipes	Kcnip4		ENSORLP00000005974
Tc kcnip	Tribolium castaneum	kcnip		XP_966445.1
Tn1 chr1	Tetraodon nigroviridis		Kcnip1	CAG01553.1
Tn3 chr4	Tetraodon nigroviridis	Kcnip3		GSTENP00017309001
Xt1 s313	Xenopus tropicalis	Kcnip1		ENSXETP00000039635
Xt4 s231	Xenopus tropicalis	Kcnip4		ENSXETP00000031644

Lbx				
**Abbreviation**	**Species**	**Current Nomenclature**	**Proposed Nomenclature**	**ID**
Bf lbx	Branchiostoma floridae	lbx		290614
Bt2 chr11	Bos taurus	Lbx2		XP_583559
Dr chr14	Danio rerio	Lbx1	Lbx2	AAH75912.1
Dr1 chr1	Danio rerio		Lbx1	XP_001333647
Dr1 chr13	Danio rerio		Lbx1	NP_001020703
Fr s52	Fugu rubripes		Lbx1	SINFRUP00000152944
Fr s70	Fugu rubripes		Lbx2	SINFRUT00000175418
Fr1 s62	Fugu rubripes		Lbx1	SINFRUP00000147148
GaIV	Gasterosteus aculeatus		Lbx1	ENSGACP00000022038
GaVI	Gasterosteus aculeatus		Lbx2	ENSGACP00000005054
GaXI	Gasterosteus aculeatus		Lbx1	ENSGACP00000024178
Gg1 chr6	Gallus gallus	Lbx1		EU339182
Gg3 chrUn	Gallus gallus	Lbx3	Lbx2	AAV32450
Hs1 chr10	Homo sapiens	LBX1		NM_006562
Hs2 chr2	Homo sapiens	LBX2		AAI50518.1
Mm1 chr19	Mus musculus	Lbx1		BAC75634.1
Mm2 chr6	Mus musculus	Lbx2		NP_034822
Olchr1	Oryzias latipes		Lbx1	UTOLAPRE05100115699
Ols1066	Oryzias latipes		Lbx2	UTOLAPRE05100119700
Sp lbx	Strongylocentrotus purpuratus	lbx		XP_791858.1
Tc lbx	Tribolium castaneum	lbx		XP_975006.1
Tn chr20	Tetraodon nigroviridis		Lbx2	GSTENT00024464001
Tn1 chr18	Tetraodon nigroviridis		Lbx1	GSTENT00035833001
Xt1	Xenopus tropicalis	Lbx1		NP_001072559.1

Ldb				
**Abbreviation**	**Species**	**Current Nomenclature**	**Proposed Nomenclature**	**ID**
Dr chr14	Danio rerio	Ldb2		NP_571389.1
Dr4 chr13	Danio rerio		Ldb1	NP_571391
Fr1 s24	Fugu rubripes	Ldb1		SINFRUG00000142748
Fr2 s70	Fugu rubripes	Ldb2		SINFRUG00000163364
Ga1 IX	Gasterosteus aculeatus	Ldb1		ENSGACG00000018336
Ga1 VI	Gasterosteus aculeatus	Ldb1		ENSGACG00000007317
Ga2 IV	Gasterosteus aculeatus	Ldb2		ENSGACG00000016642
Gg1 chr6	Gallus gallus	Ldb1		NP_990401
Gg2 chr4	Gallus gallus	Ldb2		NP_990160
Hs1 chr10	Homo sapiens	LDB1		NP_003884
Hs2 chr4	Homo sapiens	LDB2		NP_001281
Md1	Monodelphis domestica	Ldb1		XP_001369415
Md2 chr5	Monodelphis domestica	Ldb2		XP_001363147
Mm1 chr19	Mus musculus	Ldb1		NP_034827
Mm2 chr5	Mus musculus	Ldb2		NP_034828
Oa2 chr4	Oryzias latipes	Ldb2		ENSOANG00000004866
Ol1 chr1	Oryzias latipes	Ldb1		ENSORLG00000012373
Ol1 chr15	Oryzias latipes	Ldb1		ENSORLG00000008393
Sp ldb	Strongylocentrotus purpuratus	ldb2		XP_782747
Tc ldb	Tribolium castaneum	ldb2		XP_969024
Tn1 chrUn	Tetraodon nigroviridis	Ldb1		GSTENP00008527001
Xl2a	Xenopus laevis	Ldb2		BAE95407
Xl2b	Xenopus laevis	Ldb2		NP_001089184
Xt1 s640	Xenopus tropicalis	Ldb1		NP_998843

Loxl				
**Abbreviation**	**Species**	**Current Nomenclature**	**Proposed Nomenclature**	**ID**
Ag loxl	Anopheles gambiae		loxl	ENSANGP00000020312
Am loxl	Apis mellifera		loxl	XP_392090.2
Ci loxl	Ciona intestinalis		loxl	gw1.08q.592.1
Dr2a chr10	Danio rerio	Loxl2		NP_001092714
Dr2a chr10	Danio rerio	Loxl2		NP_001092714.1
Dr2b chr5	Danio rerio	Loxl2		ABM86968.1
Dr3 chr14	Danio rerio	Loxl3		XP_691519.2
Dr3 chr5	Danio rerio	Loxl3		XP_697287.2

Loxl				
**Abbreviation**	**Species**	**Current Nomenclature**	**Proposed Nomenclature**	**ID**
Dr4 chr13	Danio rerio	Loxl4		ENSDARG00000025089
Fr4s189	Fugu rubripes	Loxl4		SINFRUG00000135266
Ga2XIII	Gasterosteus aculeatus	Loxl2		ENSGACG00000005422
Ga2XVII	Gasterosteus aculeatus	Loxl2		ENSGACG00000008420
Ga3XV	Gasterosteus aculeatus	Loxl3		ENSGACG00000004902
Ga4VI	Gasterosteus aculeatus	Loxl4		ENSGALG00000013090
Gg2chr22	Gallus gallus	Loxl2		ENSGALG00000000402
Gg3 chr4	Gallus gallus	Loxl3		XP_423667.2
Hs2 chr8	Homo sapiens	LOXL2		EAW63626.1
Hs3 chr2	Homo sapiens	LOXL3		NP_115992.1
Hs4 chr10	Homo sapiens	LOXL4		NP_115587.6
Md3 chr6	Monodelphis domestica	Loxl3		XP_001376663.1
Md4 chr19	Monodelphis domestica		Loxl4	XP_001373392.1
Mm2	Mus musculus	Loxl2		P58022
Mm3 chr6	Mus musculus	Loxl3		NP_115992.1
Mm4	Mus musculus	Loxl4		EDL41893.1
Ol2chr5	Oryzias latipes	Loxl2		ENSORLG00000016809
Ol2chr9	Oryzias latipes	Loxl2		ENSORLG00000016197
Ol3chr22	Oryzias latipes	Loxl3		ENSORLG00000018185
Tn2chr12	Tetraodon nigroviridis	Loxl2		CAG12566
Tn2chrUn	Tetraodon nigroviridis	Loxl2		CAG01872
Tn4chr18	Tetraodon nigroviridis	Loxl4		CAG06063
Xla	Xenopus laevis		Loxl3	AAI29050.1
Xt4s212	Xenopus tropicalis	Loxl4		ENSXETG00000015828

Mgea				
**Abbreviation**	**Species**	**Current Nomenclature**	**Proposed Nomenclature**	**ID**
Am mgea	Apis mellifera		mgea	XP_395374.2
Bf mgea	Branchiosto ma floridae		mgea	estExt_fgenesh2_pg.C_15300 79
Dr chr13	Danio rerio	Mgea5		XP_700372.2
Fr2s186	Fugu rubripes		Mgea2	SINFRUP00000157555
Fr5b s24	Fugu	Mgea5		SINFRUP00000151553

Mgea				
Abbreviation	Species	Current Nomenclature	Proposed Nomenclature	ID
	rubripes			
Ga2 VII	Gasterosteus aculeatus		Mgea2	ENSGACP00000026279
Ga5 VI	Gasterosteus aculeatus			ENSGACP00000009681
Ga5b IX	Gasterosteus aculeatus	Mgea5		ENSGACP00000024250
Gg2 chr4	Gallus gallus	Mgea5	Mgea2	XP_427980.2
Gg5 chr6	Gallus gallus	Mgea5		NP_001034394.1
Hs5 chr10	Homo sapiens	MGEA5		NP_036347.1
Md5 chr1	Monodelphis domestica	Mgea5		XP_001369677.1
Tc mgea	Tribolium castaneum		mgea	XP_966927.1
Tn chr17	Tetraodon nigroviridis		Mgea5	CAG09361.1
Tn2 chrUn	Tetraodon nigroviridis		Mgea2	CAG10789.1
Xt s283	Xenopus tropicalis		Mgea5	estExt_fgenesh1_pg.C_2380009

Npm				
Abbreviation	Species	Current Nomenclature	Proposed Nomenclature	ID
Am npm	Apis mellifera	npm		XP_394187.2
Dr1 chr10	Danio rerio	Npm1		NP_955460.1
Dr2 chr10	Danio rerio	Npm2		AAI22198.1
Dr2 chr8	Danio rerio	Npm2		XP_001335394
Dr3 chr13	Danio rerio	Npm3		NP_001013502.1
Dr4 chr14	Danio rerio		Npm4	XP_692942.2
Fr1 s6	Fugu rubripes		Npm1	SINFRUP00000138903
Fr3 s24	Fugu rubripes	Npm3		SINFRUP00000175266
Fr4 s70	Fugu rubripes		Npm4	SINFRUP00000148214
Ga IV	Gasterosteus aculeatus		Npm4	ENSGACP00000022060
Ga VII	Gasterosteus aculeatus	Npm1		ENSGACP00000026846
Gg1 chr13	Gallus gallus	Npm1		NP_990598
Gg2 chr22	Gallus gallus	Npm2		XP_001233986
Gg3 chr6	Gallus gallus	Npm3		XP_001233764.1
Hs1 chr5	Homo sapiens	NPM1		NP_954654.1
Hs2 chr8	Homo sapiens	NPM2		NP_877724.1
Hs3 chr10	Homo sapiens	NPM3		NP_008924.1

Npm				
**Abbreviation**	**Species**	**Current Nomenclature**	**Proposed Nomenclature**	**ID**
Le4	Leucoraja erinacea		Npm4	EE991931
Le4	Leucoraja erinacea		Npm4	EE990805
Le4	Leucoraja erinacea		Npm4	EE991853
Le4	Leucoraja erinacea		Npm4	EE989832
Le4	Leucoraja erinacea		Npm4	EE991770
Le4	Leucoraja erinacea		Npm4	EE992470
Le4	Leucoraja erinacea		Npm4	EE992950
Mm1 chr11	Mus musculus	Npm1		CAI25151.1
Mm2 chr14	Mus musculus	Npm2		AAI04059.1
Mm3 chr19	Mus musculus	Npm3		XP_921243.2
Ol3 chr15	Oryzias latipes	Npm3		ENSORLP00000010662
Tc npm	Tribolium castaneum	npm		XP_973085.1
Tn1 chr7	Tetraodon nigroviridis		Npm1	GSTENT00020359001
Tn3 chr17	Tetraodon nigroviridis	Npm3		GSTENP00030609001
Tn4 chr20	Tetraodon nigroviridis		Npm4	GSTENT00024467001
Xt1 s24	Xenopus tropicalis	Npm1		NP_988883.1
Xt2 s32	Xenopus tropicalis	Npm2		NP_001016938.1
Xt3 s283	Xenopus tropicalis	Npm3		NP_001016456.1

Pcgf				
**Abbreviation**	**Species**	**Current Nomenclature**	**Proposed Nomenclature**	**ID**
Cf1 chr17	Canis familiaris	Pcgf1		XP_532995.2
Dr1 chr14	Danio rerio	Pcgf1		AAI34013.1
Fr1 s70	Fugu rubripes		Pcgf1	SINFRUP00000161523
Ga1 IV	Gasterosteus aculeatus	Pcgf1		ENSGACT00000022082
Hs1 chr2	Homo sapiens	PCGF1		NP_116062.2
Hs2 chr17	Homo sapiens	PCGF2		NP_009075.1
Hs3 chr6	Homo sapiens	PCGF3		NP_006306.2
Hs4 chr10	Homo sapiens	PCGF4		NP_005171
Hs5 chr10	Homo sapiens	PCGF5		NP_005171.4
Hs6 chr10	Homo sapiens	PCGF6		NP_001011663.1
Md1 chr5	Monodelphis domestica	Pcgf1		SINFRUP00000161523
Mm1 chr6	Mus musculus	Pcgf1		EDK99056.1
Nv pcgf1	Nematostella vectensis		pcgf1	XP_001629189.1
Sp pcgf1	Strongylocentrotus purpuratus		pcgf1	XP_001197188.1

Pcgf				
**Abbreviation**	**Species**	**Current Nomenclature**	**Proposed Nomenclature**	**ID**
Xt s47	Xenopus tropicalis	Pcgf1		NP_001016417.1

Poll				
**Abbreviation**	**Species**	**Current Nomenclature**	**Proposed Nomenclature**	**ID**
Dr chr1	Danio rerio	Poll		AAH55597.1
Fr s62	Fugu rubripes		Poll	SINFRUP00000134869
Gg chr6	Gallus gallus	Poll		XP_001232209.1
Hs chr10	Homo sapiens	POLL		NP_037406.1
HsPOLBchr8	Homo sapiens	POLB		NP_002681.1
Md chr1	Monodelphis domestica	Poll		XP_001369819.1
Mm chr19	Mus musculus	Poll		NP_064416.1
Nv poll	Nematostella vectensis		poll	XP_001627992.1
Sp poll	Strongylocentrotus purpuratus	poll		XP_001184516.1
Tn s13770	Tetraodon nigroviridis		Poll	CAG03351.1
Xt s283	Xenopus tropicalis	Poll		NP_001093716.1

Prom				
**Abbreviation**	**Species**	**Current Nomenclature**	**Proposed Nomenclature**	**ID**
Ag prom	Anopheles gambiae	prom		XP_315718.3
Bf prom	Branchiostoma floridae	prom		132160
Dr1 chr14	Danio rerio	Prom1	Prom1a	XP_700047
Dr2 chr13	Danio rerio		Prom3	XP_684527
Drl2 chr1	Danio rerio	Prom1	Prom1b	NP_932337
Fr1 s17	Fugu rubripes	Prom1	Prom1b	SINFRUG00000150818
Fr1 s70	Fugu rubripes	Prom1	Prom1a	SINFRUG00000134858
Fr2 s24	Danio rerio		Prom3	SINFRUG00000142746
Ga1 IV	Gasterosteus aculeatus	Prom1	Prom1a	ENSGACG00000016649
Ga1 IX	Gasterosteus aculeatus	Prom1	Prom1b	ENSGACG00000017039
Ga2 VI	Gasterosteus aculeatus		Prom3	ENSGACG00000007331
Gg1 chr4	Gallus gallus	Prom1		XP_001232165
Hs1 chr4	Homo sapiens	PROM1		NP_006008
Hs2 chr2	Homo sapiens	PROM2		NP_653308
Md1 chr5	Monodelphis domestica	Prom1		XP_001369554
Md2 chr1	Monodelphis domestica	Prom2		XP_001382066

Prom				
**Abbreviation**	**Species**	**Current Nomenclature**	**Proposed Nomenclature**	**ID**
Mm1 chr5	Mus musculus	Prom1		NP_032961
Mm2 chr2	Mus musculus	Prom2		NP_620089
Ol1 s115	Oryzias latipes	Prom1	Prom1a	ENSORLG00000020095
Ol2chr15	Oryzias latipes		Prom3	ENSORLG00000008367
Tc prom	Tribolium castaneum	prom		XP_973083.1
Tn2 s8962	Tetraodon nigroviridis		Prom3	GSTENG00006507001
Xt1 s393	Xenopus tropicalis	Prom1		ENSXETG00000020336
Xt2 s30	Xenopus tropicalis	Prom2		ENSXETG00000004342

Slc2a				
**Abbreviation**	**Species**	**Current Nomenclature**	**Proposed Nomenclature**	**ID**
Am slc2a	Apis mellifera		slc2a5/7/9/11/15	XP_393425.2
Bf slc2a	Branchiostoma floridae		slc2a5/7/9/11/15	97586
Dr chr1	Danio rerio		Slc2a15	NP_001018330.1
Dr chr13	Danio rerio		Slc2a15	XP_683562.2
Dr11 chr6	Danio rerio	Slc2a11		XP_688856.2
Fr s52	Fugu rubripes		Slc2a15	SINFRUP00000165501
Fr s62	Fugu rubripes		Slc2a15	SINFRUP00000132520
Gg chr6	Gallus gallus		Slc2a15	XP_426528.2
Gg11 chr15	Gallus gallus	Slc2a11		XP_425279.1
Gg5 chr21	Gallus gallus	Slc2a5		XP_417596.2
Gg9 chr4	Gallus gallus	Slc2a9		XP_420789.2
Hs11 chr22	Homo sapiens	SLC2A11		BAB83504.1
Hs5 chr1	Homo sapiens	SLC2A5		NP_003030.1
Hs7 chr1	Homo sapiens	SLC2A7		NP_997303.2
Hs9 chr4	Homo sapiens	SLC2A9		NP_064425.2
Md9	Monodelphis domestica	Slc2a9		XP_001371233.1
Mm5 chr4	Mus musculus	Slc2a5		NP_062715.2
Mm7 chr4	Mus musculus	Slc2a7		NP_001078998.1
Mm9 chr5	Mus musculus	Slc2a9		EDL37555.1
Tc slc2a	Tribolium castaneum		slc2a5/7/9/11/15	XP_973908.1
Tn chr18	Tetraodon nigroviridis		Slc2a15	GSTENT00035836001
Tn9 chr11	Tetraodon nigroviridis	Slc2a11		CAG02006.1
Xt s238	Xenopus tropicalis		Slc2a15	e_gw1.238.58.1
Xt5 s207	Xenopus tropicalis	Slc2a5		AAH82511.1

Slit				
**Abbreviation**	**Species**	**Current Nomenclature**	**Proposed Nomenclature**	**ID**
Ag slit	Anopheles gambiae	slit		XP_312121
Dr1a chr13	Danio rerio	Slit1		NP_001030140
Dr1b chr22	Danio rerio	Slit1		NP_001030147
Dr2 chr1	Danio rerio	Slit2		NP_571810
Dr3 chr14	Danio rerio	Slit3		NP_571811
Fr1 s102	Fugu rubripes	Slit1		SINFRUG00000162337
Fr1 s24	Fugu rubripes	Slit1		SINFRUG00000138133
Fr2 s261	Fugu rubripes	Slit2		SINFRUG00000159646
Fr3 s5	Fugu rubripes	Slit3		SINFRUG00000125467
Ga1 VI	Gasterosteus aculeatus	Slit1		ENSGACG00000007441
Ga2 IX	Gasterosteus aculeatus	Slit2		ENSGACG00000016196
Gg1 chr11	Gallus gallus	Slit1		XP_421715
Gg2 chr4	Gallus gallus	Slit2		XP_001232066
Gg3 chr13	Gallus gallus	Slit3		XP_414503
Hs1 Chr10	Homo sapiens	SLIT1		NP_003052
Hs2 chr4	Homo sapiens	SLIT2		NP_004778
Hs3 chr5	Homo sapiens	SLIT3		NP_003053
Md1 chr1	Monodelphis domestica	Slit1		ENSMODG00000004492
Md2 chr5	Monodelphis domestica	Slit2		XP_001368481
Md3 chr1	Monodelphis domestica	Slit3		XP_001380309
Mm1 chr11	Mus musculus	Slit1		NP_056563
Mm3 chr11	Mus musculus	Slit3		NP_035542
Oa1 chr1	Ornithorhynchus anatinus	Slit1		ENSORLG00000000055
Oa1 chr15	Ornithorhynchus anatinus	Slit1		ENSORLG00000007918
Ol2 chr1	Oryzias latipes	Slit2		ENSORLG00000005750
Ol3 chr10	Oryzias latipes	Slit3		ENSORLG00000000335
Tc slit	Tribolium castaneum	slit		XP_972265
Tn1 chr17	Tetraodon nigroviridis	Slit1		CAF94128
Tn2 s10875	Tetraodon nigroviridis	Slit2		CAF93511
Xl1	Xenopus laevis	Slit1		NP_001080578
Xl2	Xenopus laevis	Slit2		NP_001081137
Xt1 s517	Xenopus tropicalis	Slit1		NP_001090702
Xt2 s231	Xenopus tropicalis	Slit2		ENSXETP00000044252

Slit				
**Abbreviation**	**Species**	**Current Nomenclature**	**Proposed Nomenclature**	**ID**
Xt3 s228	Xenopus tropicalis	Slit3		NP_001072738

Tlx				
**Abbreviation**	**Species**	**Current Nomenclature**	**Proposed Nomenclature**	**ID**
Bf tlx	Branchiostoma floridae	tlx		290614
Bt1 chr26	Bos taurus	Tlx1		XP_581571
Bt2 chr11	Bos taurus	Tlx2		NW_001492905
Bt3 chr20	Bos taurus	Tlx3		NW_001493914
Cf1 chr28	Canis familiaris	Tlx1		XP_861821
Cf2 chr17	Canis familiaris	Tlx2		XP_855075
Cf3	Canis familiaris	Tlx3		XP_546241
Dr chr14	Danio rerio	Tlx3		ENSDARP00000008694
Dr1 chr13	Danio rerio	Tlx1		NP_739571
Dr3a chr14	Danio rerio	Tlx3a	Tlx2	NP_705937
Dr3b chr10	Danio rerio	Tlx3		XP_001331437
Fr3a s70	Fugu rubripes	Tlx3		SINFRUP00000159097
Gg1 chr6	Gallus gallus	Tlx1		NP_990346
Gg3 chr13	Gallus gallus	Tlx3		NP_990345
Hs1 chr10	Homo sapiens	TLX1		NP_005512
Hs2 chr2	Homo sapiens	TLX2		NP_057254
Hs3 chr5	Homo sapiens	TLX3		NP_066305.2
Md2 chr5	Monodelphis domestica	Tlx2		XP_001376731
Md3 chr1	Monodelphis domestica	Tlx3		NW_001581859
Mm1 chr19	Mus musculus	Tlx1		NP_068701
Mm2 chr6	Mus musculus	Tlx2		NP_033418
Mm3 chr11	Mus musculus	Tlx3		NP_064300.1
Sp tlx	Strongylocentrotus purpuratus	tlx		XP_001175911.1
Tc tlx	Tribolium castaneum	tlx		XP_974983.1
Xl1	Xenopus laevis	Tlx1		NP_001079216
Xt s313	Xenopus tropicalis	Tlx3		477366
Xt1 s238	Xenopus tropicalis	Tlx1		171787

References:

1. Burgers PM, Koonin EV, Bruford E, Blanco L, Burtis KC, Christman MF, Copeland WC, Friedberg EC, Hanaoka F, Hinkle DC, et al: **Eukaryotic DNA polymerases: proposal for a revised nomenclature.** *J Biol Chem* 2001, **276:**43487-43490.

2. Shevelev I, Blanca G, Villani G, Ramadan K, Spadari S, Hubscher U, Maga G: **Mutagenesis of human DNA polymerase lambda: essential roles of Tyr505 and Phe506 for both DNA polymerase and terminal transferase activities.** *Nucleic Acids Res* 2003, **31:**6916-6925.
3. Zariwala M, O'Neal WK, Noone PG, Leigh MW, Knowles MR, Ostrowski LE: **Investigation of the possible role of a novel gene, DPCD, in primary ciliary dyskinesia.** *Am J Respir Cell Mol Biol* 2004, **30:**428-434.
4. Ianakiev P, Kilpatrick MW, Dealy C, Kosher R, Korenberg JR, Chen XN, Tsipouras P: **A novel human gene encoding an F-box/WD40 containing protein maps in the SHFM3 critical region on 10q24.** *Biochem Biophys Res Commun* 1999, **261:**64-70.
5. Jin J, Cardozo T, Lovering RC, Elledge SJ, Pagano M, Harper JW: **Systematic analysis and nomenclature of mammalian F-box proteins.** *Genes Dev* 2004, **18:**2573-2580.
6. Hediger MA, Romero MF, Peng JB, Rolfs A, Takanaga H, Bruford EA: **The ABCs of solute carriers: physiological, pathological and therapeutic implications of human membrane transport proteinsIntroduction.** *Pflugers Arch* 2004, **447:**465-468.
7. Uldry M, Thorens B: **The SLC2 family of facilitated hexose and polyol transporters.** *Pflugers Arch* 2004, **447:**480-489.
8. Kato A, Kawamata N, Tamayose K, Egashira M, Miura R, Fujimura T, Murayama K, Oshimi K: **Ancient ubiquitous protein 1 binds to the conserved membrane-proximal sequence of the cytoplasmic tail of the integrin alpha subunits that plays a crucial role in the inside-out signaling of alpha IIbbeta 3.** *J Biol Chem* 2002, **277:**28934-28941.
9. Gearhart MD, Corcoran CM, Wamstad JA, Bardwell VJ: **Polycomb group and SCF ubiquitin ligases are found in a novel BCOR complex that is recruited to BCL6 targets.** *Mol Cell Biol* 2006, **26:**6880-6889.
10. Garcia-Fernandez J: **The genesis and evolution of homeobox gene clusters.** *Nat Rev Genet* 2005, **6:**881-892.
11. Muller T, Brohmann H, Pierani A, Heppenstall PA, Lewin GR, Jessell TM, Birchmeier C: **The homeodomain factor lbx1 distinguishes two major programs of neuronal differentiation in the dorsal spinal cord.** *Neuron* 2002, **34:**551-562.
12. Cheng L, Samad OA, Xu Y, Mizuguchi R, Luo P, Shirasawa S, Goulding M, Ma Q: **Lbx1 and Tlx3 are opposing switches in determining GABAergic versus glutamatergic transmitter phenotypes.** *Nat Neurosci* 2005, **8:**1510-1515.
13. Kruger M, Schafer K, Braun T: **The homeobox containing gene Lbx1 is required for correct dorsal-ventral patterning of the neural tube.** *J Neurochem* 2002, **82:**774-782.
14. Gross MK, Dottori M, Goulding M: **Lbx1 specifies somatosensory association interneurons in the dorsal spinal cord.** *Neuron* 2002, **34:**535-549.
15. Dietrich S, Schubert FR, Healy C, Sharpe PT, Lumsden A: **Specification of the hypaxial musculature.** *Development* 1998, **125:**2235-2249.
16. Brohmann H, Jagla K, Birchmeier C: **The role of Lbx1 in migration of muscle precursor cells.** *Development* 2000, **127:**437-445.
17. Schafer K, Braun T: **Early specification of limb muscle precursor cells by the homeobox gene Lbx1h.** *Nat Genet* 1999, **23:**213-216.
18. Gross MK, Moran-Rivard L, Velasquez T, Nakatsu MN, Jagla K, Goulding M: **Lbx1 is required for muscle precursor migration along a lateral pathway into the limb.** *Development* 2000, **127:**413-424.
19. Martin BL, Harland RM: **A novel role for lbx1 in Xenopus hypaxial myogenesis.** *Development* 2006, **133:**195-208.

20. Neyt C, Jagla K, Thisse C, Thisse B, Haines L, Currie PD: **Evolutionary origins of vertebrate appendicular muscle.** *Nature* 2000, **408:**82-86.
21. Wei K, Chen J, Akrami K, Sekhon R, Chen F: **Generation of mice deficient for Lbx2, a gene expressed in the urogenital system, nervous system, and Pax3 dependent tissues.** *Genesis* 2007, **45:**361-368.
22. Wu G, Xu G, Schulman BA, Jeffrey PD, Harper JW, Pavletich NP: **Structure of a beta-TrCP1-Skp1-beta-catenin complex: destruction motif binding and lysine specificity of the SCF(beta-TrCP1) ubiquitin ligase.** *Mol Cell* 2003, **11:**1445-1456.
23. Cameron EA, Martinez-Marignac VL, Chan A, Valladares A, Simmonds LV, Wacher N, Kumate J, McKeigue P, Shriver MD, Kittles R, et al: **MGEA5-14 polymorphism and type 2 diabetes in Mexico City.** *Am J Hum Biol* 2007, **19:**593-596.
24. Wells L, Gao Y, Mahoney JA, Vosseller K, Chen C, Rosen A, Hart GW: **Dynamic O-glycosylation of nuclear and cytosolic proteins: further characterization of the nucleocytoplasmic beta-N-acetylglucosaminidase, O-GlcNAcase.** *J Biol Chem* 2002, **277:**1755-1761.
25. Shibata Y, Tsukazaki T, Hirata K, Xin C, Yamaguchi A: **Role of a new member of IGFBP superfamily, IGFBP-rP10, in proliferation and differentiation of osteoblastic cells.** *Biochem Biophys Res Commun* 2004, **325:**1194-1200.
26. Matthews JM, Visvader JE: **LIM-domain-binding protein 1: a multifunctional cofactor that interacts with diverse proteins.** *EMBO Rep* 2003, **4:**1132-1137.
27. Miraglia S, Godfrey W, Yin AH, Atkins K, Warnke R, Holden JT, Bray RA, Waller EK, Buck DW: **A novel five-transmembrane hematopoietic stem cell antigen: isolation, characterization, and molecular cloning.** *Blood* 1997, **90:**5013-5021.
28. Florek M, Bauer N, Janich P, Wilsch-Braeuninger M, Fargeas CA, Marzesco AM, Ehninger G, Thiele C, Huttner WB, Corbeil D: **Prominin-2 is a cholesterol-binding protein associated with apical and basolateral plasmalemmal protrusions in polarized epithelial cells and released into urine.** *Cell Tissue Res* 2007, **328:**31-47.
29. Maw MA, Corbeil D, Koch J, Hellwig A, Wilson-Wheeler JC, Bridges RJ, Kumaramanickavel G, John S, Nancarrow D, Roper K, et al: **A frameshift mutation in prominin (mouse)-like 1 causes human retinal degeneration.** *Hum Mol Genet* 2000, **9:**27-34.
30. Dixon DN, Izon DJ, Dagger S, Callow MJ, Taplin RH, Kees UR, Greene WK: **TLX1/HOX11 transcription factor inhibits differentiation and promotes a non-haemopoietic phenotype in murine bone marrow cells.** *Br J Haematol* 2007, **138:**54-67.
31. Cheng L, Arata A, Mizuguchi R, Qian Y, Karunaratne A, Gray PA, Arata S, Shirasawa S, Bouchard M, Luo P, et al: **Tlx3 and Tlx1 are post-mitotic selector genes determining glutamatergic over GABAergic cell fates.** *Nat Neurosci* 2004, **7:**510-517.
32. Langenau DM, Palomero T, Kanki JP, Ferrando AA, Zhou Y, Zon LI, Look AT: **Molecular cloning and developmental expression of Tlx (Hox11) genes in zebrafish (Danio rerio).** *Mech Dev* 2002, **117:**243-248.
33. Jourdan-Le Saux C, Tronecker H, Bogic L, Bryant-Greenwood GD, Boyd CD, Csiszar K: **The LOXL2 gene encodes a new lysyl oxidase-like protein and is expressed at high levels in reproductive tissues.** *J Biol Chem* 1999, **274:**12939-12944.
34. Itoh A, Miyabayashi T, Ohno M, Sakano S: **Cloning and expressions of three mammalian homologues of Drosophila slit suggest possible roles for Slit in the formation and maintenance of the nervous system.** *Brain Res Mol Brain Res* 1998, **62:**175-186.
35. Wong K, Park HT, Wu JY, Rao Y: **Slit proteins: molecular guidance cues for cells ranging from neurons to leukocytes.** *Curr Opin Genet Dev* 2002, **12:**583-591.

36. Niclou SP, Jia L, Raper JA: **Slit2 is a repellent for retinal ganglion cell axons.** *J Neurosci* 2000, **20:**4962-4974.
37. Hohenester E, Hussain S, Howitt JA: **Interaction of the guidance molecule Slit with cellular receptors.** *Biochem Soc Trans* 2006, **34:**418-421.
38. Di Cristofano A, Carpino N, Dunant N, Friedland G, Kobayashi R, Strife A, Wisniewski D, Clarkson B, Pandolfi PP, Resh MD: **Molecular cloning and characterization of p56dok-2 defines a new family of RasGAP-binding proteins.** *J Biol Chem* 1998, **273:**4827-4830.
39. Wick MJ, Dong LQ, Hu D, Langlais P, Liu F: **Insulin receptor-mediated p62dok tyrosine phosphorylation at residues 362 and 398 plays distinct roles for binding GTPase-activating protein and Nck and is essential for inhibiting insulin-stimulated activation of Ras and Akt.** *J Biol Chem* 2001, **276:**42843-42850.
40. Niki M, Di Cristofano A, Zhao M, Honda H, Hirai H, Van Aelst L, Cordon-Cardo C, Pandolfi PP: **Role of Dok-1 and Dok-2 in leukemia suppression.** *J Exp Med* 2004, **200:**1689-1695.
41. Ng CH, Xu S, Lam KP: **Dok-3 plays a nonredundant role in negative regulation of B-cell activation.** *Blood* 2007, **110:**259-266.
42. Itoh N: **The Fgf families in humans, mice, and zebrafish: their evolutional processes and roles in development, metabolism, and disease.** *Biol Pharm Bull* 2007, **30:**1819-1825.
43. Crossley PH, Martinez S, Martin GR: **Midbrain development induced by FGF8 in the chick embryo.** *Nature* 1996, **380:**66-68.
44. Tanaka S, Ueo H, Mafune K, Mori M, Wands JR, Sugimachi K: **A novel isoform of human fibroblast growth factor 8 is induced by androgens and associated with progression of esophageal carcinoma.** *Dig Dis Sci* 2001, **46:**1016-1021.
45. Hoshikawa M, Ohbayashi N, Yonamine A, Konishi M, Ozaki K, Fukui S, Itoh N: **Structure and expression of a novel fibroblast growth factor, FGF-17, preferentially expressed in the embryonic brain.** *Biochem Biophys Res Commun* 1998, **244:**187-191.
46. Ohbayashi N, Hoshikawa M, Kimura S, Yamasaki M, Fukui S, Itoh N: **Structure and expression of the mRNA encoding a novel fibroblast growth factor, FGF-18.** *J Biol Chem* 1998, **273:**18161-18164.
47. Bolli N, Galimberti S, Martelli MP, Tabarrini A, Roti G, Mecucci C, Martelli MF, Petrini M, Falini B: **Cytoplasmic nucleophosmin in myeloid sarcoma occurring 20 years after diagnosis of acute myeloid leukaemia.** *Lancet Oncol* 2006, **7:**350-352.
48. Burns KH, Viveiros MM, Ren Y, Wang P, DeMayo FJ, Frail DE, Eppig JJ, Matzuk MM: **Roles of NPM2 in chromatin and nucleolar organization in oocytes and embryos.** *Science* 2003, **300:**633-636.
49. Shackleford GM, Ganguly A, MacArthur CA: **Cloning, expression and nuclear localization of human NPM3, a member of the nucleophosmin/nucleoplasmin family of nuclear chaperones.** *BMC Genomics* 2001, **2:**8.
50. Scannevin RH, Wang K, Jow F, Megules J, Kopsco DC, Edris W, Carroll KC, Lu Q, Xu W, Xu Z, et al: **Two N-terminal domains of Kv4 K(+) channels regulate binding to and modulation by KChIP1.** *Neuron* 2004, **41:**587-598.
51. Shibata R, Misonou H, Campomanes CR, Anderson AE, Schrader LA, Doliveira LC, Carroll KI, Sweatt JD, Rhodes KJ, Trimmer JS: **A fundamental role for KChIPs in determining the molecular properties and trafficking of Kv4.2 potassium channels.** *J Biol Chem* 2003, **278:**36445-36454.
